# Supplementary material for: Whole-genome sequence-based association analysis of African American individuals with bipolar disorder and schizophrenia
Source: HGG Adv. 2025 Aug 30;7(1):100499. doi: 10.1016/j.xhgg.2025.100499 (PMC12494821; doi:10.1016/j.xhgg.2025.100499)
Supplement: Document S1. Figures S1–S14, Tables S1–S10, and supplemental text [file mmc1.pdf]

HGGA, Volume 7

Supplemental information

Whole-genome sequence-based association analysis of African American individuals with bipolar disorder and schizophrenia

Runjia Li, Sarah A. Gagliano Taliun, Kevin Liao, Matthew Flickinger, Janet L. Sobell, Giulio Genovese, Adam E. Locke, Rebeca Rothwell Chiu, Jonathon LeFaive, Jiongming Wang, Taylor Martins, Sinéad Chapman, Anna Neumann, Robert E. Handsaker, Donna K. Arnett, Kathleen C. Barnes, Eric Boerwinkle, David Braff, Brian E. Cade, Myriam Fornage, Richard A. Gibbs, Karin F. Hoth, Lifang Hou, Charles Kooperberg, Ruth J.F. Loos, Ginger A. Metcalf, Courtney G. Montgomery, Alanna C. Morrison, Zhaohui S. Qin, Susan Redline, Alexander P. Reiner, Stephen S. Rich, Jerome I. Rotter, Kent D. Taylor, Karine A. Viaud-Martinez, National Heart, Lung, and Blood Institute Trans-Omics for Precision Medicine (TOPMed) Consortium, Genomic Psychiatry Cohort Investigators, Tim B. Bigdeli, Stacey Gabriel, Sebastian Zollner, Albert V. Smith, Goncalo Abecasis, Steve A. McCarroll, Michele T. Pato, Carlos N. Pato, Michael Boehnke, James Knowles, Hyun Min Kang, Roel A. Ophoff, Jason Ernst, and Laura J. Scott

Supplemental Information

Table of Contents

TOPMed Banner Authors 2

The Genomic Psychiatry Cohort: Partners in Discovery authorships 3

Supplemental Text 4

Supplemental Tables 7

Supplemental Figures 17

TOPMed Study Short Acknowledgements with dbGaP accession numbers 33

TOPMed Study-specific Acknowledgements 35

TOPMed Neurocognitive Working Group 40

Supplemental References 45

TOPMed Banner Authors

(Full list of authors can be found in Table S11.)

The Genomic Psychiatry Cohort: Partners in Discovery authorships

Michele T. Pato[1], Janet L. Sobell[1], Helena Medeiros[1], Colony Abbott[1], Brooke Skar[1], Peter F. Buckley[2], Evelyn J. Bromet[3], Michael A. Escamilla[4], Ayman H. Fanous[5], Douglas S. Lehrer[6], Fabio Macciardi[7], Dolores Malaspina[8], Steve A. McCarroll[9,10], Stephen R. Marder[11], Jennifer Moran[9], Christopher P. Morley[12], Humberto Nicolini[13], Diana O. Perkins[14], Shaun M. Purcell[15], Mark H. Rapaport[16], Pamela Sklar[17], Jordan W. Smoller[18], James A. Knowles[1], Genomic Psychiatry Cohort Consortium*, and Carlos N. Pato[1]

1 Department of Psychiatry and the Behavioral Sciences, University of Southern California, Los Angeles, CA, USA
2 Department of Psychiatry, Georgia Regents University, Augusta, GA, USA
3 Department of Psychiatry and Behavioral Science, State University of New York, Stony Brook, NY, USA
4 Department of Psychiatry, Texas Tech University Health Sciences Center, El Paso, TX, USA 5 Department of Psychiatry, Veterans Administration Medical Center, Washington, DC, USA
6 Department of Psychiatry, Wright State University, Dayton, OH, USA
7 Department of Psychiatry, University of California, Irvine, CA, USA
8 Department of Psychiatry, New York University, New York, NY, USA
9 Stanley Center for Psychiatric Research, Broad Institute of MIT and Harvard, Cambridge, MA, USA
10 Department of Genetics, Harvard Medical School, Boston, MA, USA
11 Department of Psychiatry, University of California, Los Angeles, CA, USA
12 Departments of Family Medicine, Public Health and Preventive Medicine, and Psychiatry and Behavioral Science, State University of New York, Upstate Medical University, Syracuse, NY, USA 13 Carracci Medical Group, Mexico City, MX
14 Department of Psychiatry, University of North Carolina, Chapel Hill, NC, USA
15 Center for Human Genome Research, Massachusetts General Hospital, Boston, MA, USA
16 Department of Psychiatry and Behavioral Sciences, Emory University, Atlanta, GA, USA
17 Department of Psychiatry, Mt. Sinai School of Medicine, New York, NY, USA
18 Department of Psychiatry, Harvard University, Boston, MA, USA

Supplemental Text

Follow-up analyses for the top window across all case-control test combinations

We conducted follow-up analyses for the top window across all six case-control test combinations, that is chr11:64,859,972-64,869,939 ($p=4.06 \times 10^{-8}$ with repeats and $p=1.12 \times 10^{-9}$ without repeats for InPSYght BD vs. InPSYght controls) (**Figure S13A, B**).

We first analyzed the extent to which the most significant variants could explain the overall association signal. To do this we first performed single-variant two-sided Fisher's exact test (Methods) on each of the 197 variants not overlapping repeats in the region for InPSYght BD cases versus controls. The 12 most significant variants in this region had p-values within range [1.5×10^{-4}, 1.5×10^{-2}] while no other individual variant was marginally significant (p-value <0.05). 11 out of these 12 variants are noncoding, with chr11:64859972G,A being a synonymous coding variant. When we restricted the burden test to only these 12 variants, we observed a p-value of 9.0×10^{-8} whereas performing the same test on all other variants in the window only yields a p-value of 4.8×10^{-3}, indicating a major contribution from the 12 nominally significant variants. These variants constitute two distinct haplotypes, both of which being abundant among samples African American individuals (**Figure S13C**).

The *EHD1* gene which overlaps the significant window was previously shown to play potential roles in neuropsychiatric disorders. Earlier studies established EHD1 as mediators of synaptic exo- and endocytosis[1–3]. A small-sample WES study for BD trios identified a *de novo* frame-shift mutation within the *EHD1* gene[3] and a follow-up knock-in mice study suggested that this mutation induces BD-like behavior in mouse models[4].

In addition, the significant window was notable in that it sits in a region of the genome that is dense for common variants based on the most recent PGC BD GWAS study of common variants[5]. Three distinct common variants associated within BD are within 2 Mb and five are within 6 Mb (**Table S9**). Relative to other 10kb windows in the genome this window was in the top 0.037% and 0.097% in terms of the maximum distance to the third and fifth closest variants respectively. The nearest lead variant at genome-wide significant loci for BD was 622kb away. Interestingly the window overlaps a near-significant loci (p-value ~7.0×10^{-8}) in the most recent PGC SZ GWAS study[6], while the closest genome-wide significant loci for SZ (p-value =1.8×10^{-9}) is 0.8 Mb away (**Figure S14B**).

We also investigated the chromatin state context of the window. We used chromatin state annotations of the genome for 127 cell and tissue types based on a 25-state ChromHMM model previously learned from 12 marks based on imputed data[7,8]. This region was notable in that the prevalence of the TxReg (transcribed and regulatory) state, which is associated with concurrently transcribed, promoter and enhancer chromatin marks, broadly across the 10kb window and across cell and tissue types (**Figure S14A, S14B**). On average 44.5% of bases in the 10kb window were assigned to the TxReg state in a cell or tissue type. The next most prevalent state in the window was TxEnh5' which covered 29.6% of bases on average and shared high levels of enhancer marks and some transcription marks with the TxReg state. To quantify how extreme the prevalence of these states are in the window relative to the rest of the genome, for each of the 10kb windows throughout the genome we computed the percentage of 200 base pair bins across all cell and tissue types that are assigned to

each state. The significant window ranked in the top 0.02% of all 10kb windows in the genome based on state TxReg and the top 0.08% based on TxEnh5' (**Figure S14C**). These results motivated us to inspect the most extreme locations in the genome in terms of the density of TxReg. Notably, the top 1st and 3rd ranking windows that represent unique loci are only less than 0.6 Mb away and overlap with the transcriptional sites of the long noncoding RNAs (lncRNAs) NEAT1 (chr11: 65,422,774-65,445,540) and MALAT1 (chr11: 65,497,688-65,506,516) respectively, both of which are distinguished among lncRNAs in their broad binding across the genome[9]. Notably, as indicated by GRID-seq (capturing of global RNA interactions with DNA by deep sequencing) data on the MM.1S cell line[9], the significant window resides in a broader region enriched for both *NEAT1* and *MALAT1* binding sites (**Figure S14B**). There has been emerging evidence linking lncRNAs to various brain disorders[10,11]. Specifically, studies have shown that NEAT1 is down-regulated in SZ[12] while *MALAT1* is down-regulated in BD[13].

Supplemental Tables

Table S1. InPSYght study samples by sub-cohort and case/control status

Study of origin	BD (N)	SZ (N)	Control (N)
GPC	1475	2855	2338
COGS	0	432	313
NIMH Repository: (STEP-BD, BIGS, Litmus)	123	8	0

Table S2. Source of TOPMed controls from 11 TOPMed studies

TOPMed study	Samples used as controls (N)	Sequencing Center
Cleveland Family Study (sleep apnea)	2	University of Washington (UW)
Barbados Asthma Genetics Study (BAGS)	997	Illumina
Genetic Epidemiology of COPD	3171	Broad
Multi-Ethnic Study of Atherosclerosis (MESA)	1207	Broad
African American Coronary Artery Calcification (AACAC); substudy of MESA	127	Broad
Atherosclerosis Risk in Communities (ARIC+VTE)	295	Baylor
Women's Health Initiative (WHI)	1455	Broad
Hypertension Genetic Epidemiology Network (HyperGen/GENOA)	2988	University of Washington (UW)
Sarcoidosis in African Americans	632	Baylor
BioMe Biobank	1360	Washington University (WashU)
CARDIA	1578	Baylor

Table S3. InPSYght sample demographic and sequencing wave characteristics

	SZ	BD	SZ + BD	Control	TOTAL
**N**	3295	1598	4893	2651	7544
**Females N (%)**	1036 (31%)	771 (48%)	1807 (34%)	1352 (51%)	3159 (42%)
**Age Years (SD) (N non-missing)**	43.7 (12.2) (3236)	43.1 (11.7) (1521)	43.5 (12.0) (4757)	40.6 (13.6) (2631)	42.5 (12.7) (7388)
**Sequencing Wave 0**	112	5	117	114	231
**1**	165	165	330	161	491
**2**	161	165	326	165	491
**3**	599	118	717	344	1061
**4**	1019	223	1242	516	1758
**5**	795	512	1307	744	2051
**6**	444	410	854	607	1461

Table S4. InPSYght sample quality control exclusions

Quality control	Individuals removed (N)
Duplicates	366
Sex mismatches	20
Non-XX/XY sex karyotypes	17
DNA contamination >5%	4
<98% of sites not at sequencing depth of 10	14
Unqualified controls	10

Table S5. Descriptions of the sequencing quality metrics and their loadings in the first four sequencing metadata PCs

Metric	PC1 Loading	PC2 Loading	PC3 Loading	PC4 Loading	Description
VB_DEPTH	0.70	0.72	0.02	0.03	Average depth estimated by verifyBamID2 (in array genotyped sites)
FREEMIX	0.00	0.00	0.00	-0.01	Contamination estimates by verifyBamID2
CONTAM_PC1	0.00	0.00	0.00	0.01	Estimated ancestry PC1,2,3,4 of contaminating samples estimated by verifyBamID2
CONTAM_PC2	0.00	0.00	0.01	-0.04	
CONTAM_PC3	0.00	0.00	0.00	0.00	
CONTAM_PC4	0.00	0.00	0.00	0.00	
FRAC_DP1	0.00	0.00	0.00	0.00	Fraction of bases covered by depths 1,5,10,15,20,30
FRAC_DP5	0.00	0.00	0.00	0.00	
FRAC_DP10	0.00	0.00	0.00	-0.01	
FRAC_DP15	0.00	0.00	0.00	-0.11	
FRAC_DP20	0.01	0.00	0.01	-0.52	
FRAC_DP30	0.03	0.01	0.03	-0.84	
RDP_X	0.00	-0.02	0.77	0.02	Relative depth of X (compared to autosomes)
RDP_Y	0.00	0.02	-0.64	-0.02	Relative depth of Y (compared to autosomes)
AUTO_DP	0.72	-0.70	-0.02	0.01	Autosome depth

Table S6. Summary of single-variant GWAS results. Significant threshold set to p<5E-9. Variant called on GRCh38.

Sample group 1	Sample group 2	Genomic inflation factor (λ)	Number of significant loci (variant(s)).	Notes
InPSYght controls	TOPMed controls	1.02	1 (chr13:79615934:CT:C)	Variant failed SVM filtering pipeline in TOPMed previous freeze; just passed filters in freeze9 (freeze used in current paper)
InPSYght BD + SZ	InPSYght + TOPMed controls	1.01	0 (0)	
	InPSYght controls	0.996	0 (0)	
InPSYght BD	InPSYght + TOPMed controls	1.02	1 (chr18:49738979:G:T; chr18:49741194:A:G; chr18:49744269:GGT:G)	No longer significant with inclusion of meta-PCs
	InPSYght controls	0.996	0 (0)	
InPSYght SZ	InPSYght + TOPMed controls	1.00	0 (0)	
	InPSYght controls	0.997	0 (0)	

Table S7. Non-reference allele frequencies of the 3 significant chromosome 18 variants (InPSYght bipolar versus InPSYght + TOMed controls single-variant association analysis) in cases, controls and the total TOPMed study *Obtained from files from the

chr:bp:ref:alt (GRCh38)	rsID	Alternate allele frequency						
		InPSYght			TOPMed	InPSYght +TOPMed	Total TOPMed	
		BD	SZ	Control s	Controls	Controls	AFR*	EUR*
Chr18: 49738979:G:T	rs796645723	0.0069	0.0014	0.00057	0.0012	0.0011	0.0033	n/a
Chr18: 49741194:A:G	rs566405039	0.0069	0.0014	0.00075	0.0012	0.0011	0.0033	n/a
Chr18: 49744269:GGT:G	rs796880023	0.0069	0.0014	0.00057	0.0012	0.0011	0.0031	n/a

download page of https://imputationserver.sph.umich.edu/rsq-browser/

Table S8: Top 10 genes from burden testing of PTV variants with MAF < 1% in SZ + BD cases vs controls. * denotes a pseudogene or RNA gene.

Gene ID	Gene Symbol	OR	P-Value
ENSG00000186106	*ANKRD46*	0.4334	3.261e-06
ENSG00000133243	*BTBD2*	1.8568	6.980e-06
ENSG00000032444	*PNPLA6*	0.3177	1.193e-05
ENSG00000105278	*ZFR2*	2.9034	2.176e-04
ENSG00000250100	*RP13-644M16.3**	69.1349	6.465e-04
ENSG00000004534	*RBM6*	0.5863	9.170e-04
ENSG00000197093	*GAL3ST4*	0.5332	1.023e-03
ENSG00000137501	*SYTL2*	1.7598	1.090e-03
ENSG00000167604	*NFKBID*	0.5842	1.405e-03
ENSG00000232347	*RP11-488L18.8**	0.3703	1.532e-03

Table S9. Comparison of singleton PTV burden test results from top ten implicated schizophrenia genes identified in SCHEMA.

Gene	InPSYght (P-value)	SCHEMA OR (95% CI)	SCHEMA (P-value)
SETD1A	0.86	20.1 (5.68-108)	2.00e-12
CUL1	*NA*	36.1 (5.01-1570)	2.01e-09
XPO7	0.86	52.2 (7.84-2190)	7.18e-09
TRIO	0.89	5.02 (2.47-10.4)	6.35e-08
CACNA1G	0.85	3.09 (1.21-7.63)	4.57e-07
SP4	*NA*	9.37 (3.38-29.7)	5.08e-07
GRIA3	*NA*	Inf (4.73-Inf)	5.98e-07
GRIN2A	*NA*	18.1 (3.74-172)	7.37e-07
HERC1	0.31	3.51 (2.04-6.03)	1.26e-06
RB1CC1	0.53	10 (2.89-43.9)	2.00e-06

Table S10. Distances from the significant chromosome 11 window (chr11:64,859,972-64,869,939) to the closest GWAS loci reported in a PGC BD GWAS study[5], and the percentage of all 10kb genomic windows with smaller distance to their closest GWAS loci.

Nth closest GWAS locus	Distance to GWAS locus (bp)	Percentage of 10kb genomic windows with smaller distance to its Nth closest GWAS loci
1	622,550	2.59%
2	1,216,310	0.47%
3	1,692,155	0.037%
4	3,013,821	0.046%
5	5,806,865	0.097%
6	14,516,526	0.40%

Supplemental Figures

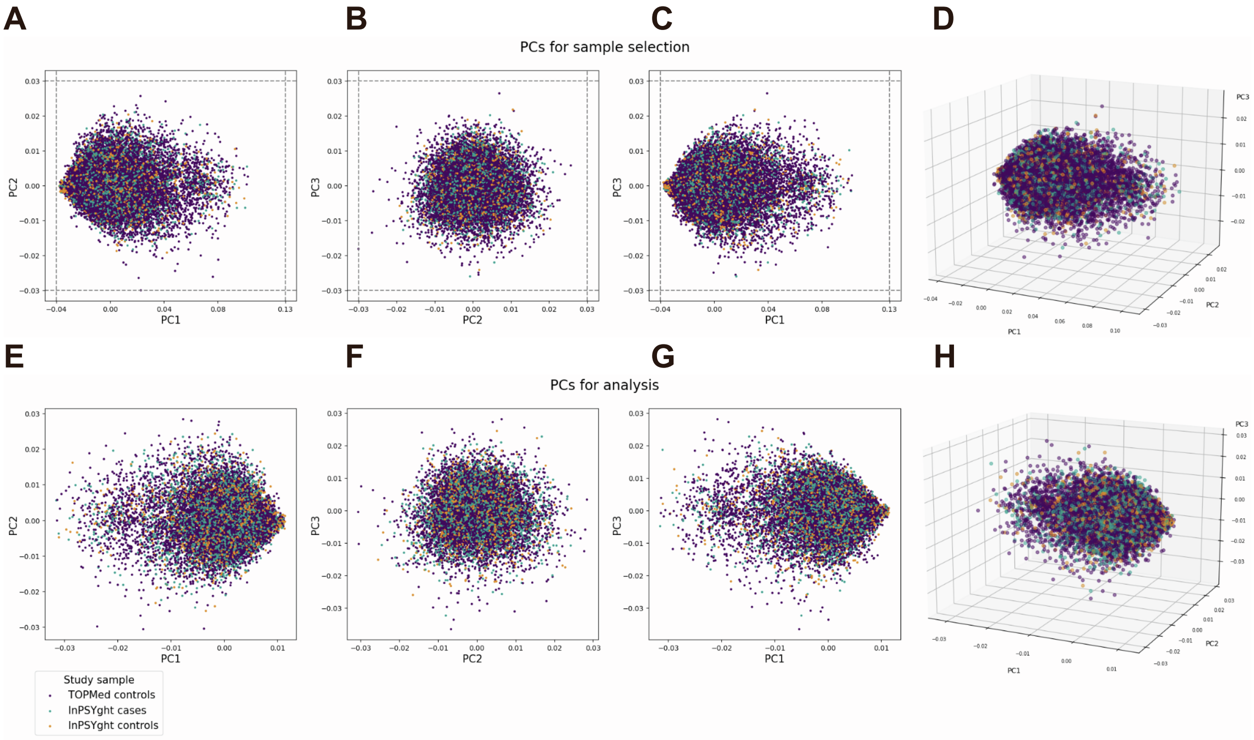

Figure S1. The first three genetic principal components (PCs) for the InPSYght and TOPMed samples. (A-D) PCs created using WGS'd samples, to further define WGS samples for inclusion. Only included samples are shown in the plots. Dashed lines indicate PC thresholds used for selection of included samples. (A) PC1 vs. PC2, (B) PC2 vs. PC3, (C) PC1 vs. PC3, and (D) PCs 1-3 in the 3D space. (E-H) PCs for association analysis created using WGS data from samples included in analysis. (E) PC1 vs. PC2, (F) PC2 vs. PC3, (G) PC1 vs. PC3 and (H) PCs 1-3 in the 3D space.

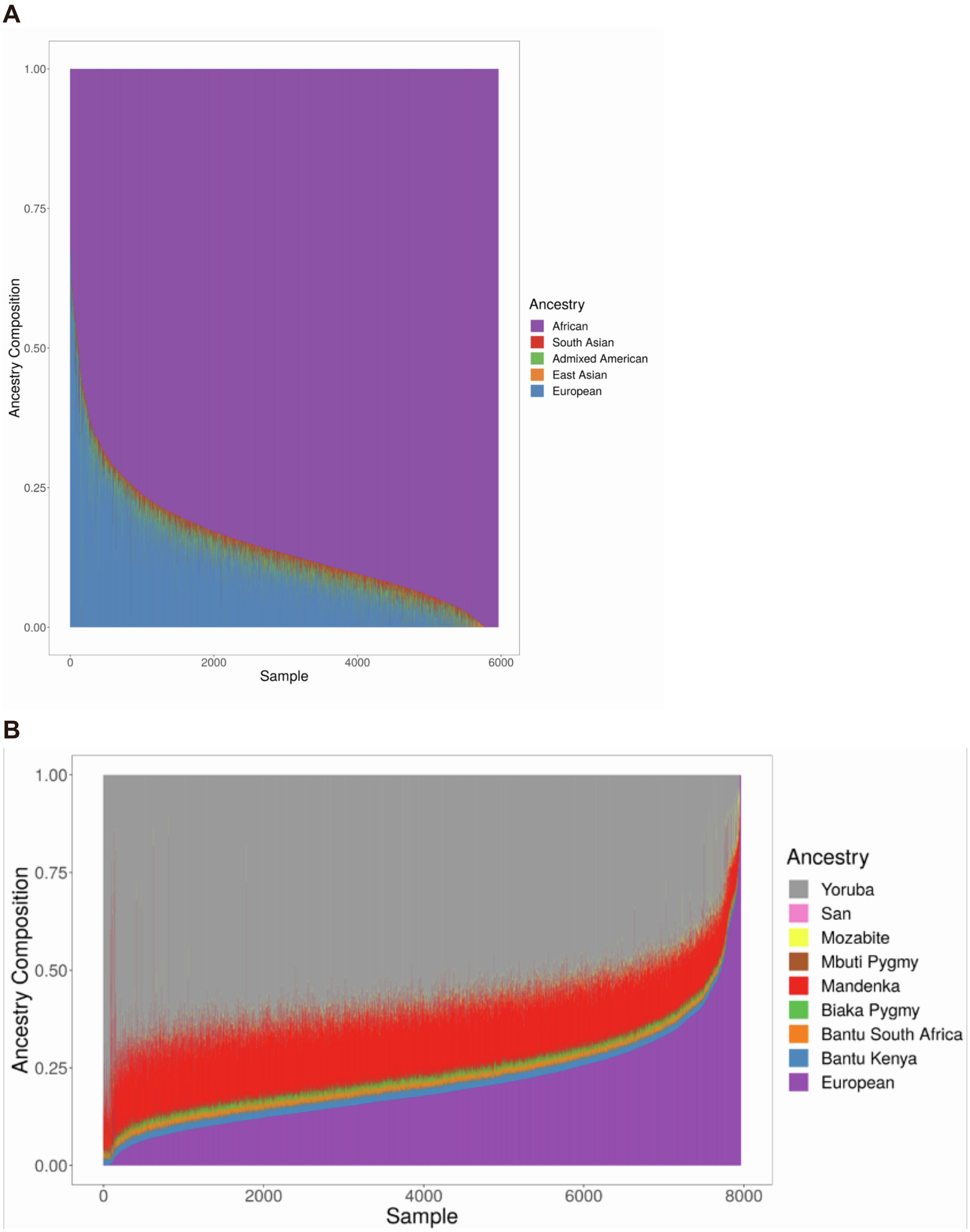

Figure S2. Estimated African and European ancestry composition of African American individuals within the InPSYght study. Global ancestry composition of African American individuals within the InPSYght study using 1000 Genomes super-populations (A) and

within African sub-populations (B) Reference populations include European as a super population and African sub populations from the HGDP reference panel. Note this ancestry analysis differs from the 1000 Genomes Project phase 3 ancestry super-populations ancestry estimation analysis from which proportion European ancestry was estimated for InPSYght study inclusion. The 1000 Genomes Project based analysis including Admixed Americans (AMR), South Asian (SAS), and East Asian (EAS) individuals.

A

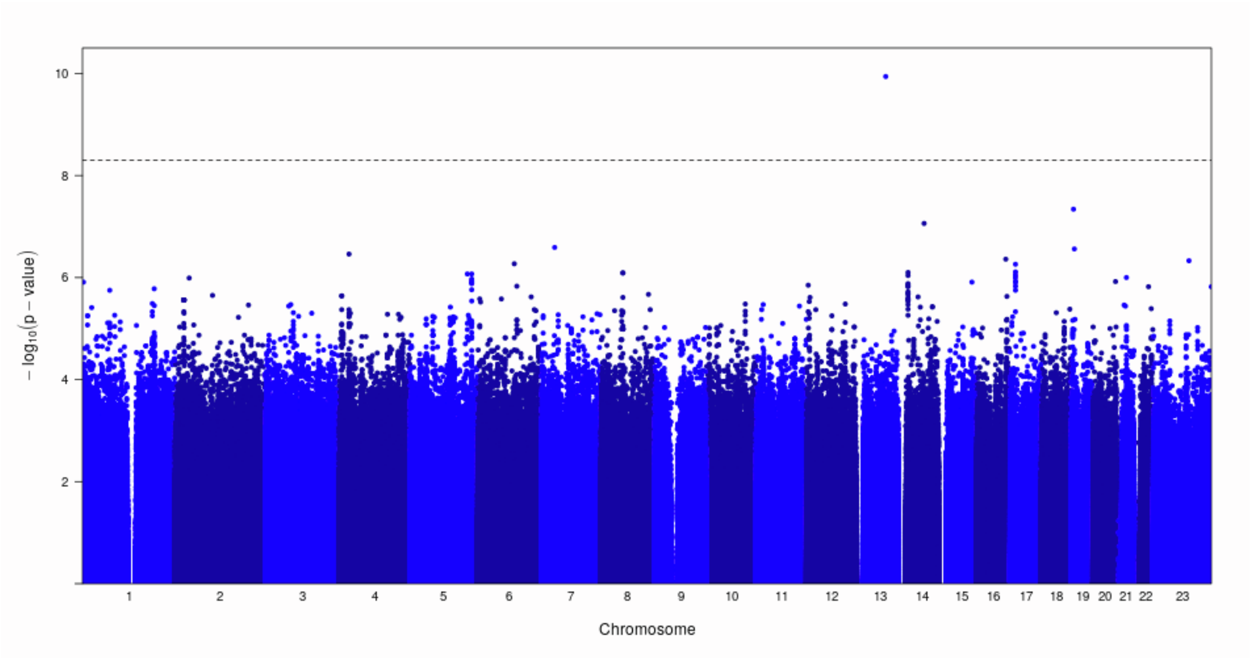

B

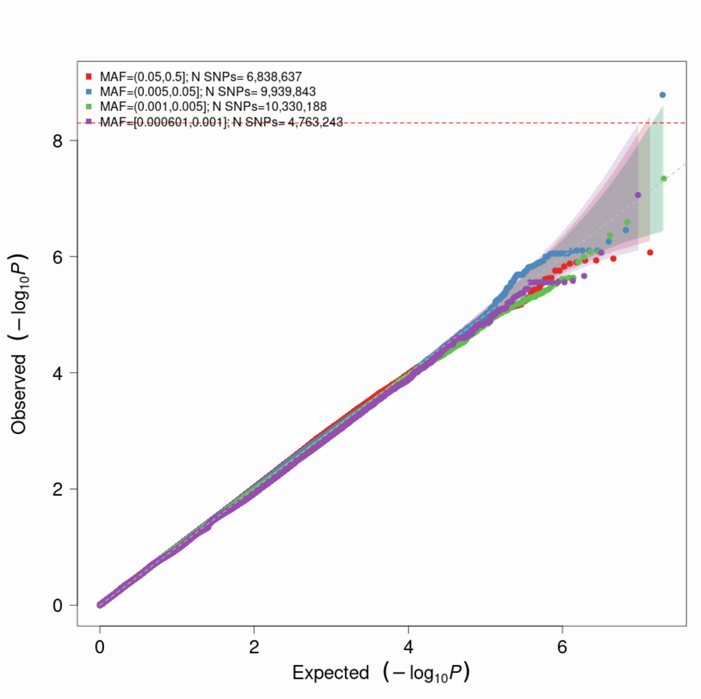

Figure S3. InPSYght controls versus TOPMed controls with sex and 10 PCs as covariates stratified by MAF of variants: Manhattan (A) and Quantile-Quantile Plots (B). Lambda GC: 1.02. Horizontal line shows genome-wide significance threshold of 5×10^{-9}.

A

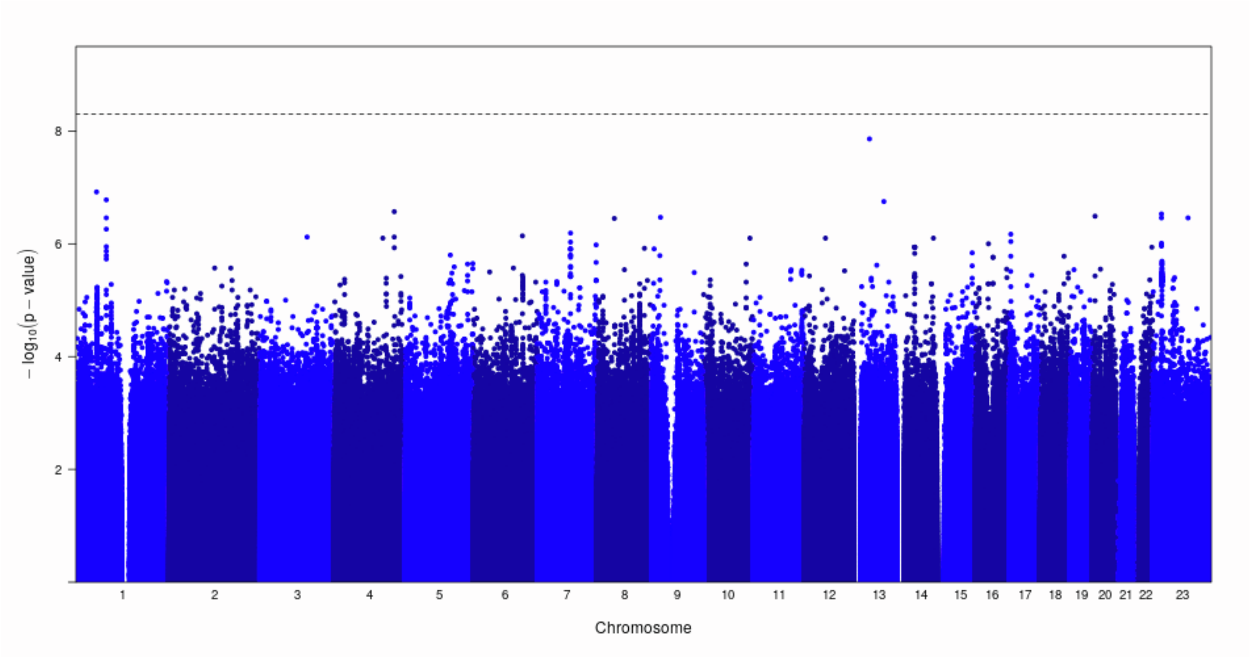

B

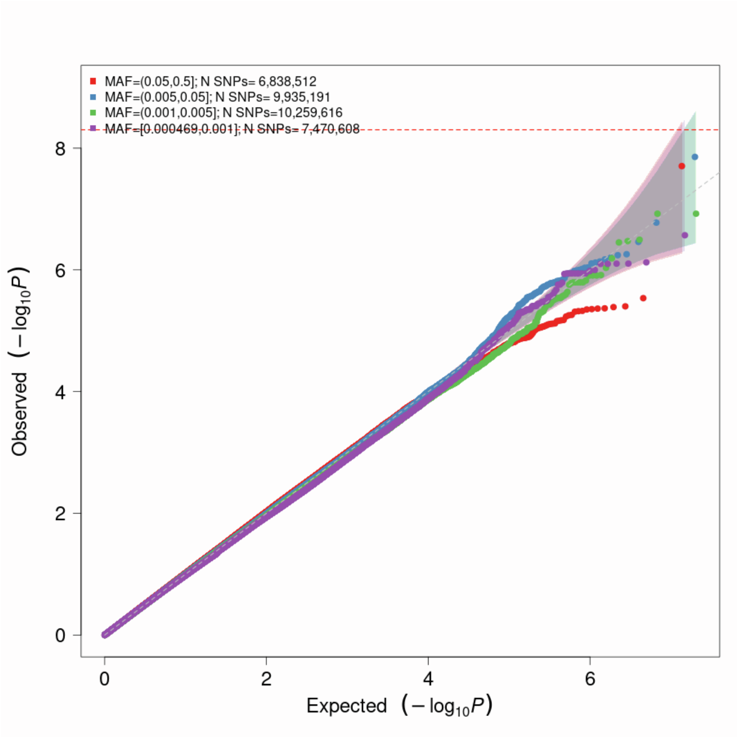

Figure S4. InPSYght cases with InPSYght + TOPMed controls with sex and 10 PCs as covariates stratified by MAF of variants: Manhattan (A) and Quantile-Quantile Plots (B). Lambda GC: 1.01. Horizontal line shows genome-wide significance threshold of 5 x 10^{-9}.

A

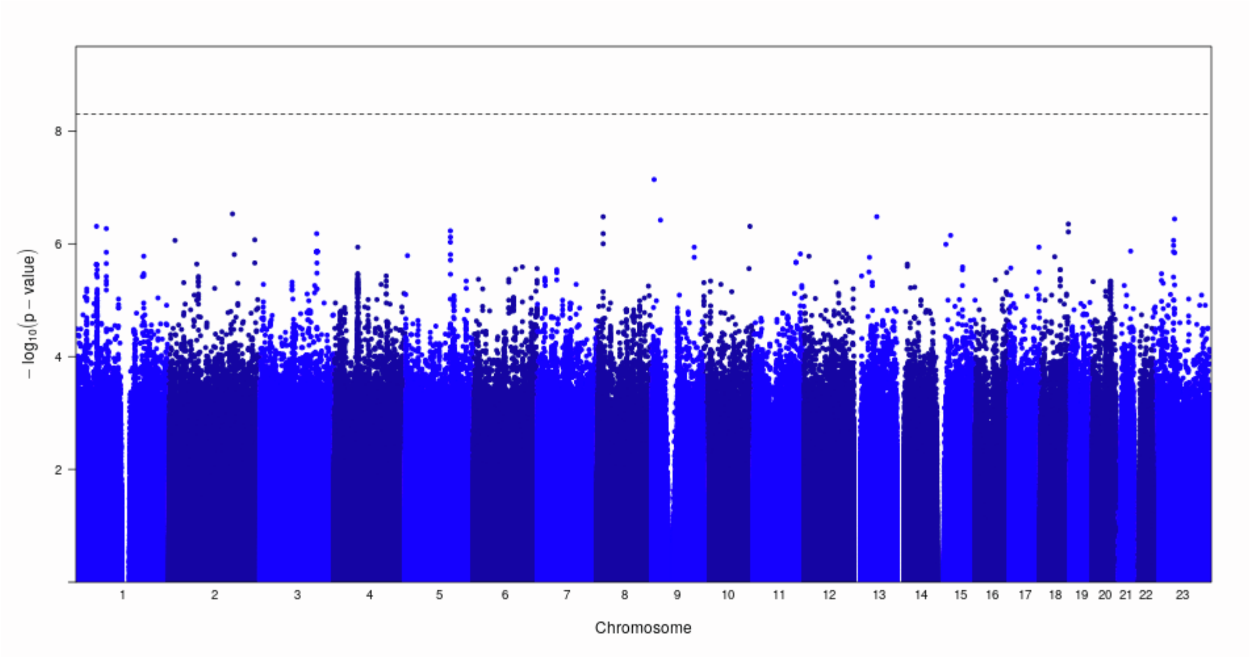

B

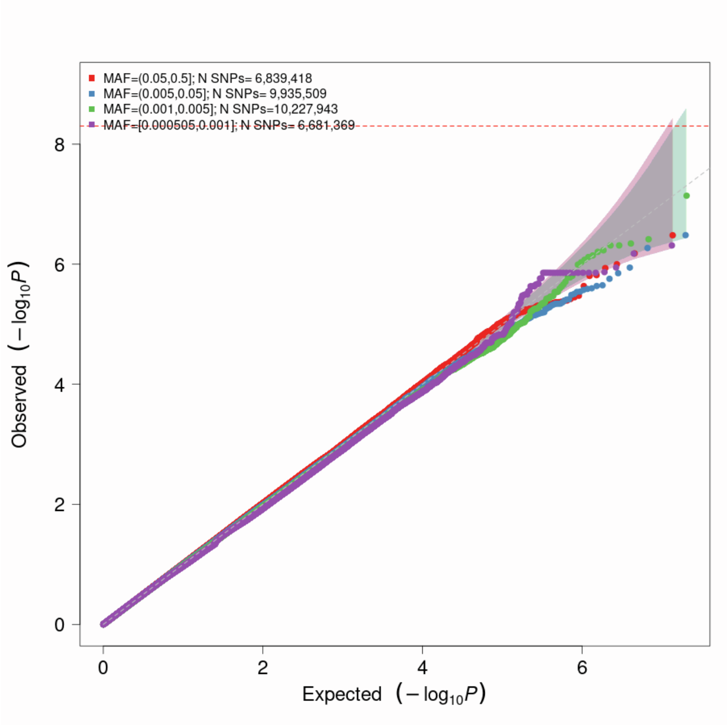

Figure S5. InPSYght schizophrenia cases versus InPSYght + TOPMed controls with sex and 10 PCs as covariates stratified by MAF of variants: Manhattan (A) and Quantile-Quantile Plots (B). Lambda GC: 1.00. Horizontal line shows genome-wide significance threshold of 5 x 10^{-9}.

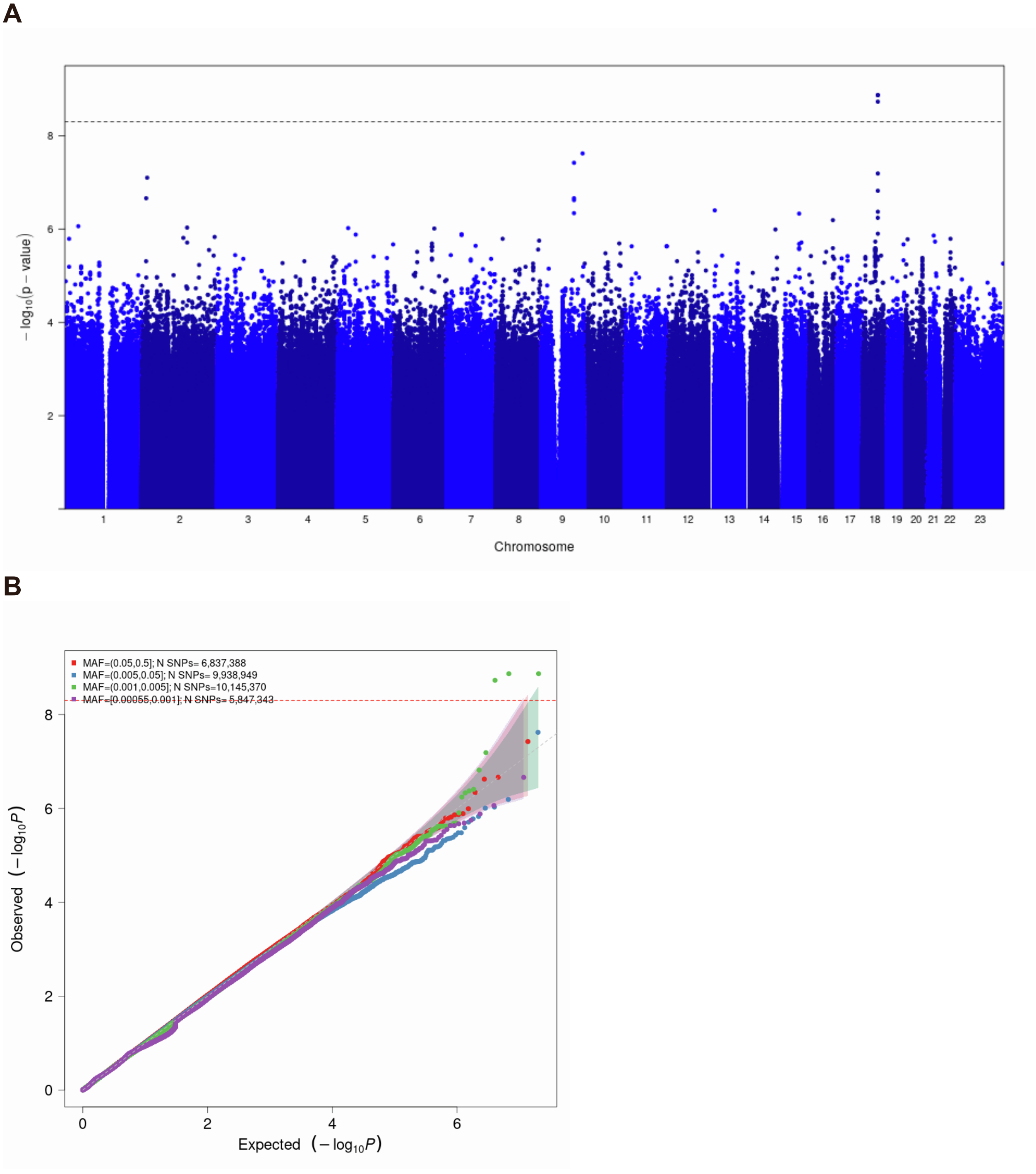

Figure S6. InPSYght bipolar cases versus InPSYght + TOPMed controls with sex and 10 PCs as covariates stratified by MAF of variants: Manhattan (A) and Quantile-Quantile Plots (B). Lambda GC: 1.02. Note: the Manhattan (Figure S6a is also presented as a main figure.) Horizontal line shows genome-wide significance threshold of 5 x 10^{-9}.

A

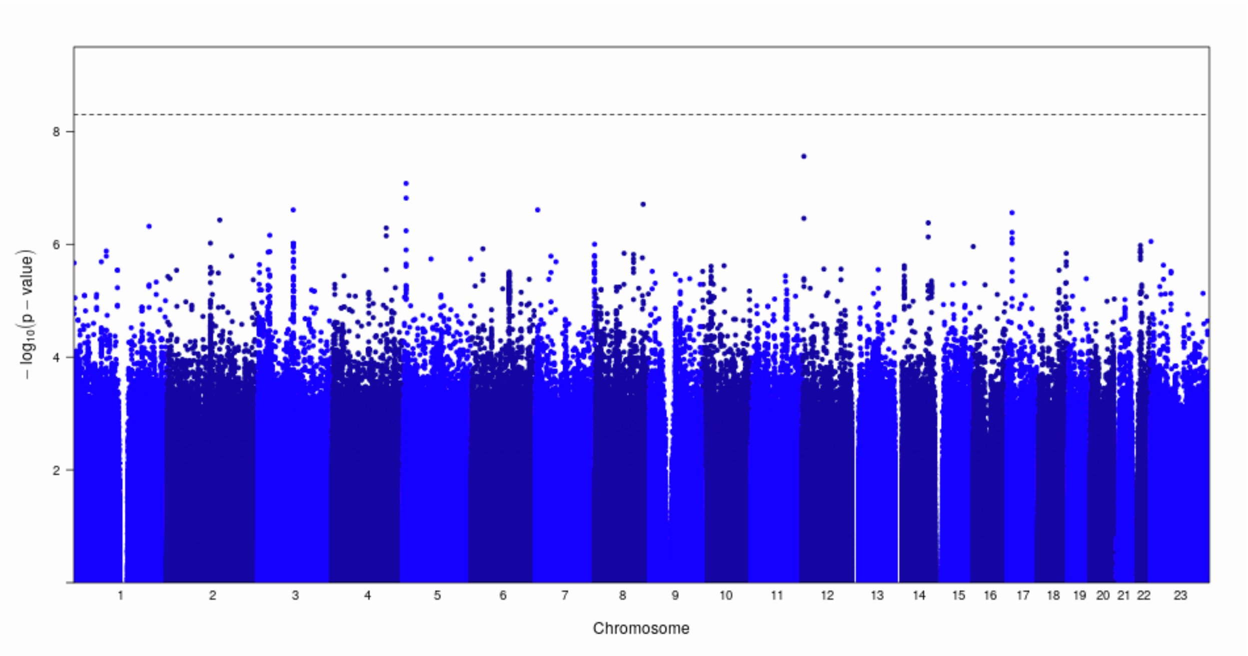

B

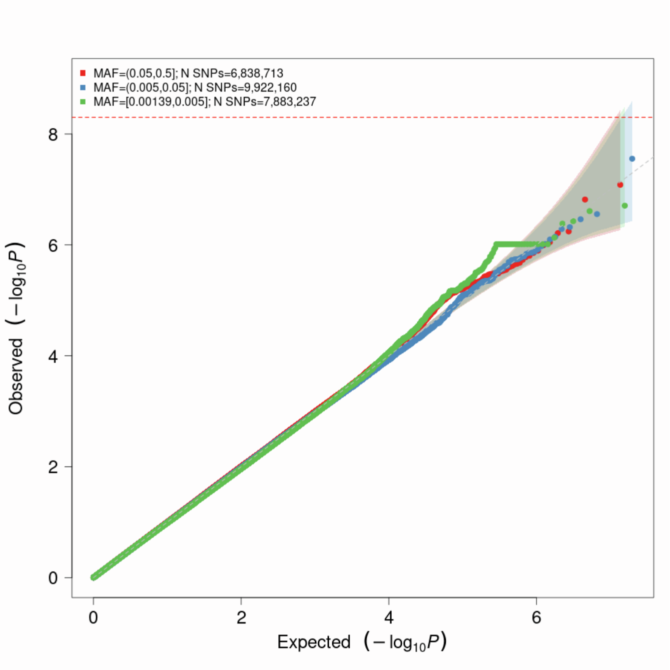

Figure S7. InPSYght-only cases versus controls with sex, batch and 10 PCs as covariates stratified by MAF of variants: Manhattan (A) and Quantile-Quantile Plots (B). Lamb GC: 1.00. Horizontal line shows genome-wide significance threshold of 5×10^{-9}.

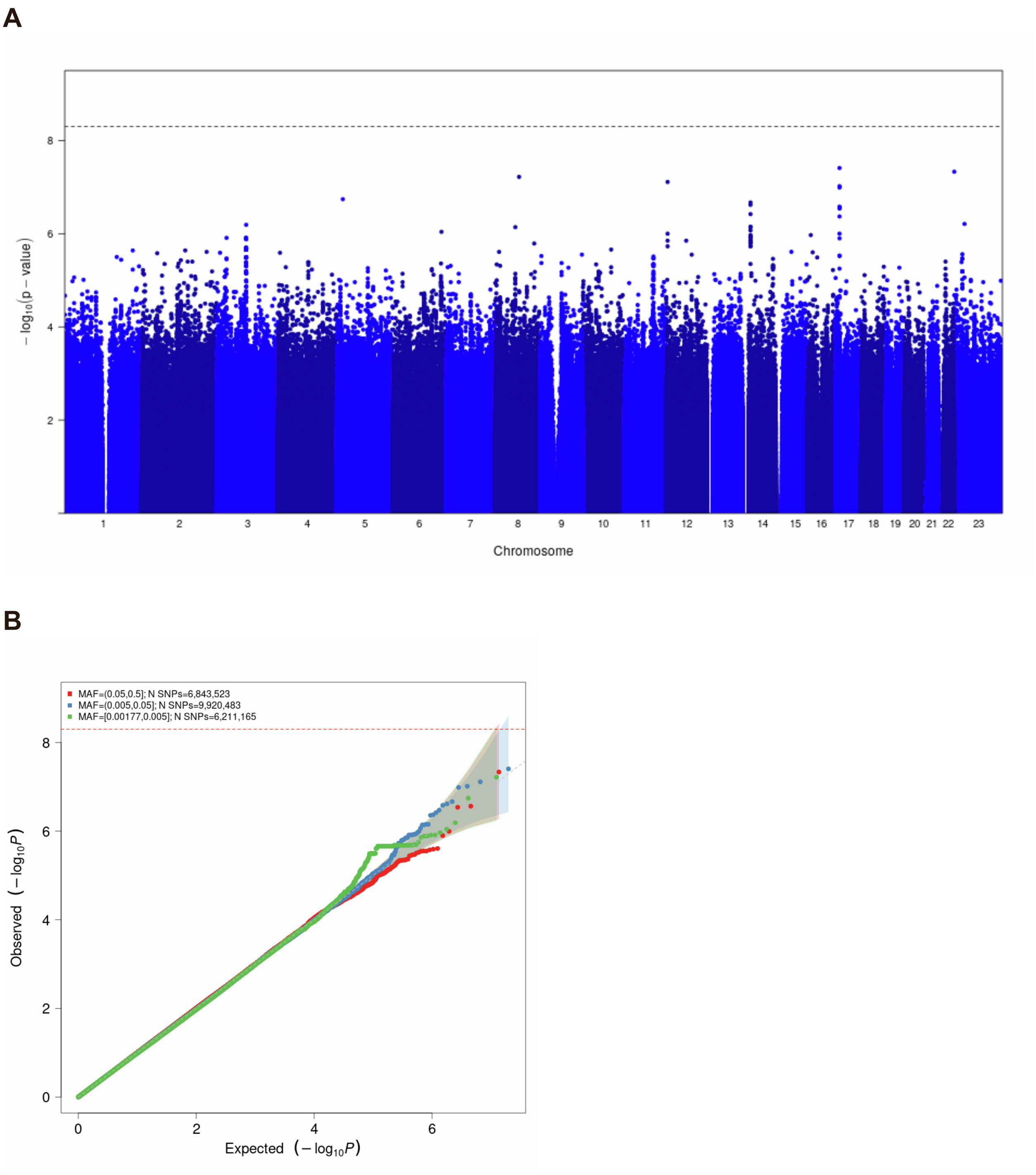

Figure S8. InPSYght-only schizophrenia cases versus controls with sex, batch and 10 PCs as covariates stratified by MAF of variants: Manhattan (A) and Quantile-Quantile Plots (B). Lambda GC: 1.00. Horizontal line shows genome-wide significance threshold of 5 x 10^{-9}.

A

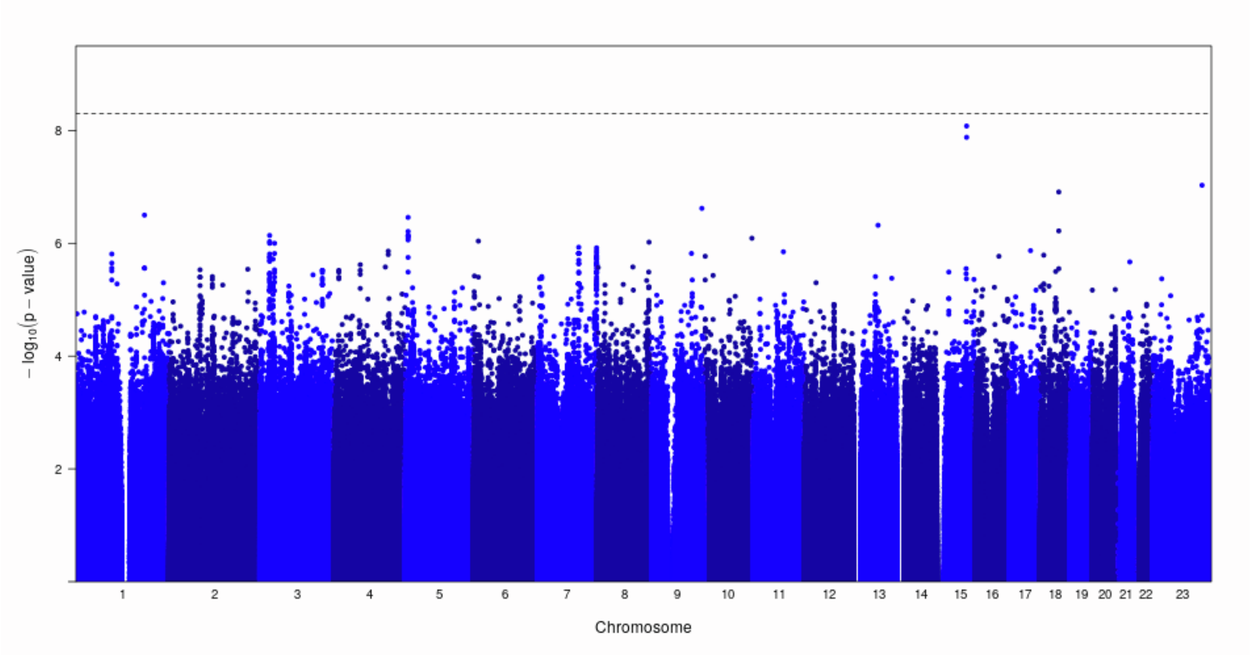

B

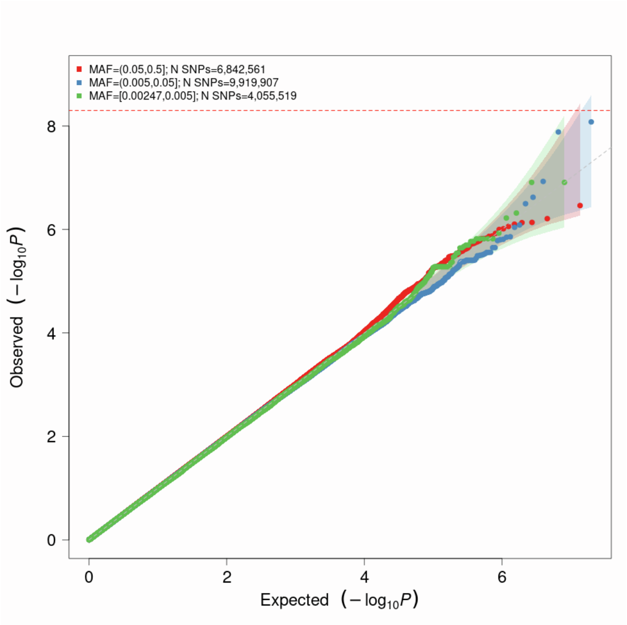

Figure S9. InPSYght-only bipolar cases versus controls with sex, batch and 10 PCs as covariates stratified by MAF of variants: Manhattan (A) and Quantile-Quantile Plots (B). Lambda GC: 1.00. Horizontal line shows genome-wide significance threshold of 5 x 10^{-9}.

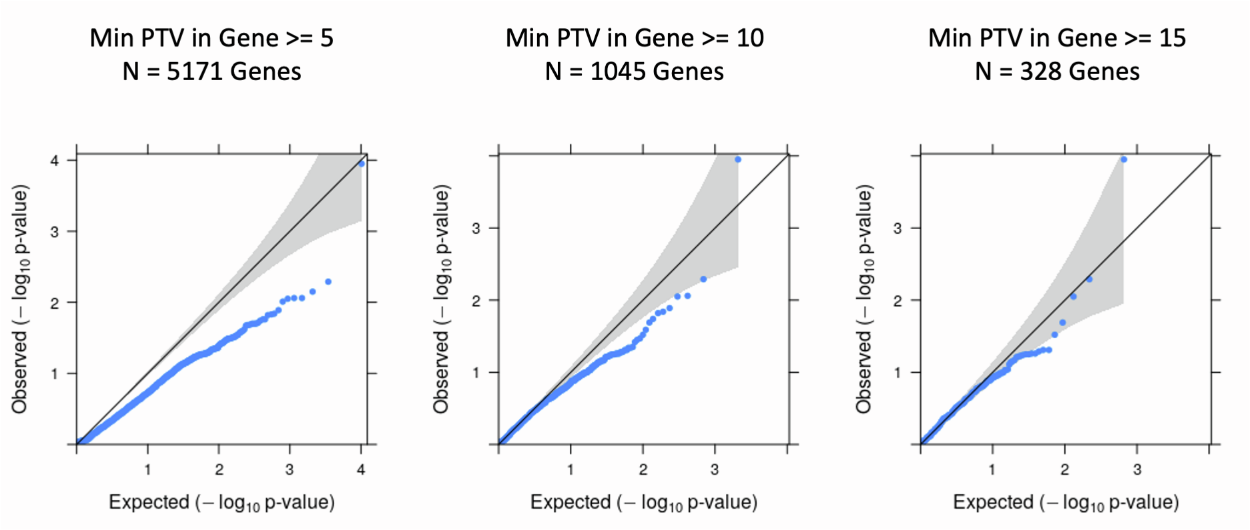

Figure S10. QQ plot of genome wide gene-level singleton PTV burden tests in unrelated individuals by minimum number of PTV criteria for gene inclusion. Covariates adjusted for include genetic PC1-10, sex, and total number of singletons.

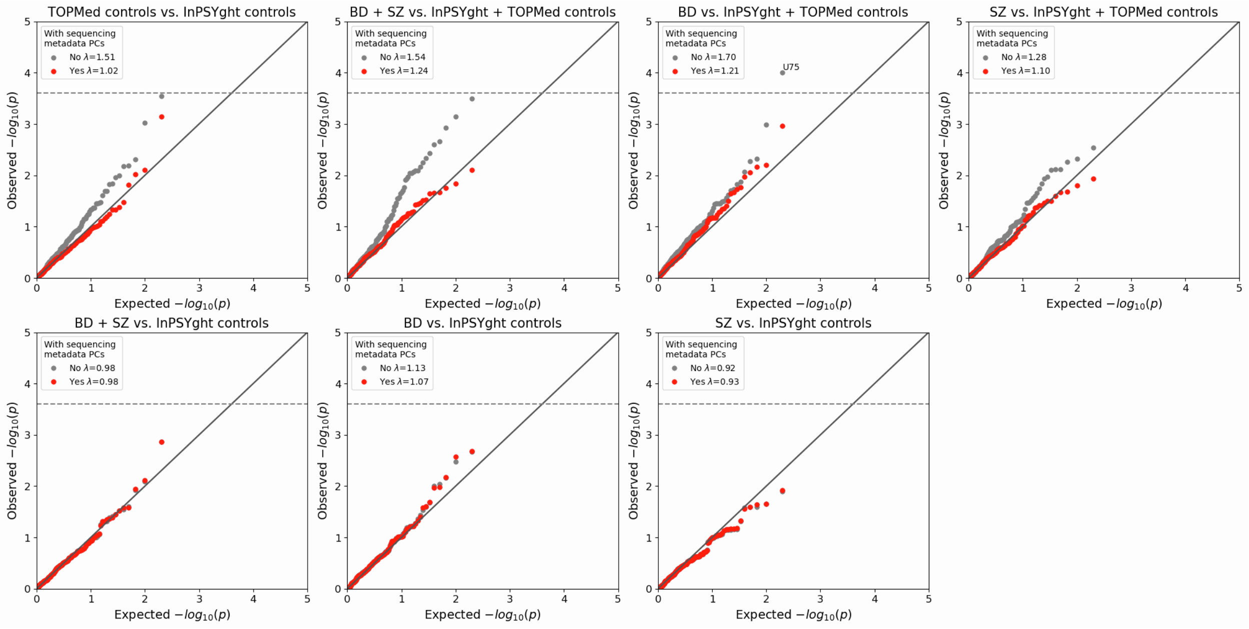

Figure S11. QQ-plots of chromatin and conservation state burden test results for different combinations of samples, showing their genomic inflation factors (λ) before and after including sequencing metadata PCs in the covariates. Horizontal lines show Bonferroni-based p-value thresholds (p=0.05/200). Diagonal lines show the unit slope.

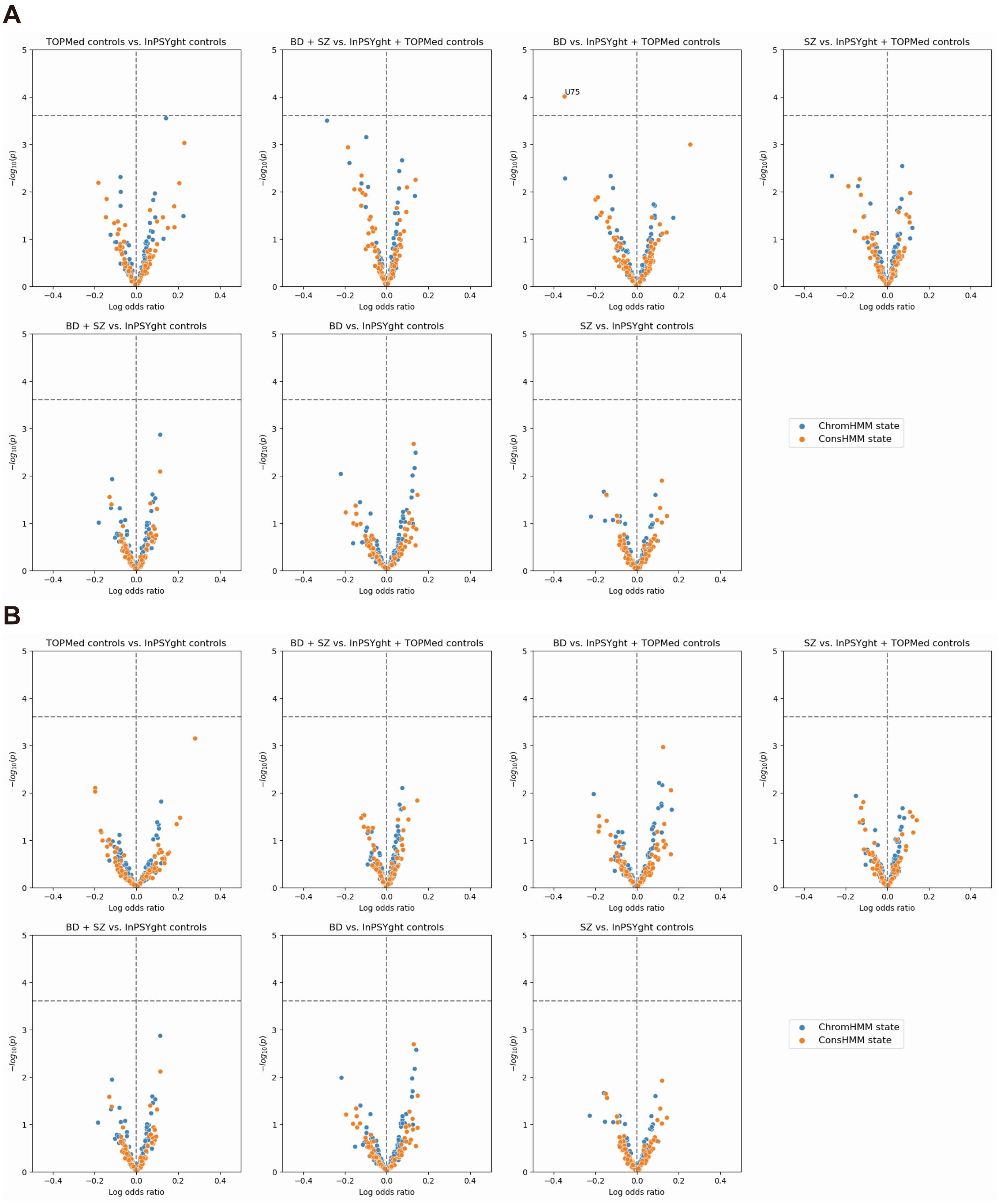

Figure S12. Volcano plots of chromatin and conservation state burden test results for different combinations of samples, before (A) and after (B) using sequencing metadata PCs as covariates, with log odds ratio on the x-axis and -log10 p-values on the y-axis. Vertical lines show x=0. Horizontal lines show Bonferroni-based p-value thresholds (p=0.05/200).

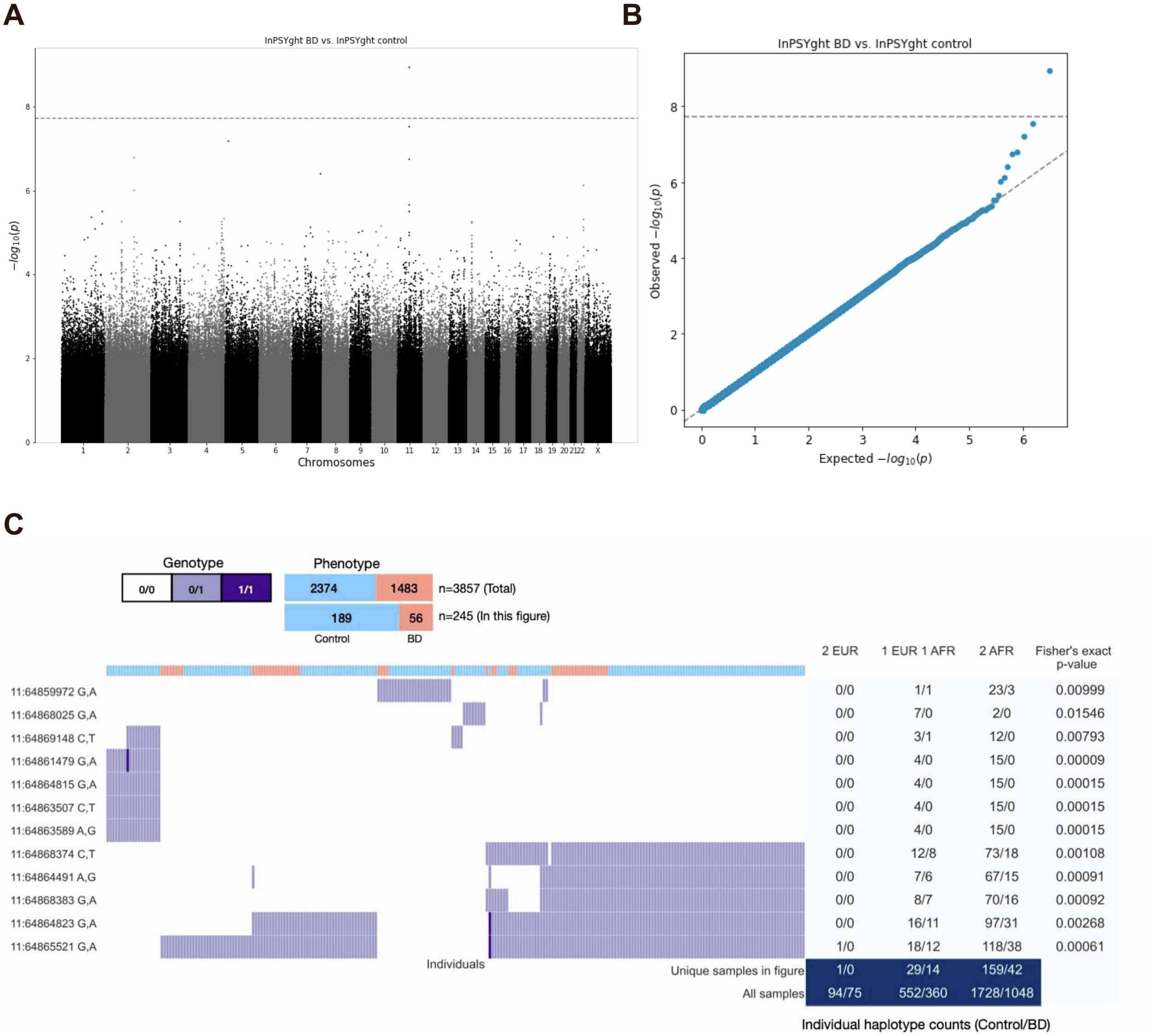

Figure S13. Summary of WGScan burden test results for InPSYght BD cases versus controls, with genomic repeat regions removed. BD: bipolar disorder. (**A**) Manhattan plot of all windows being tested; dashed line shows the Bonferroni-corrected p-value threshold based on WGScan's estimated number of effective tests (0.05 / 2,463,399 = 2.0×10^{-8}). (**B**) Quantile-quantile plot for burden p-values of all windows being tested. (**C**) Sample genotypes for the significant window chr11:64,859,972-64,859,939. The sites with nominally significant Fisher's exact p-values and the samples with at least 1 alternative allele observation among these sites are shown in the heatmap. The table on the right shows the ancestry haplotype distributions for samples having each variant and the Fisher's exact p-values for each variant.

A

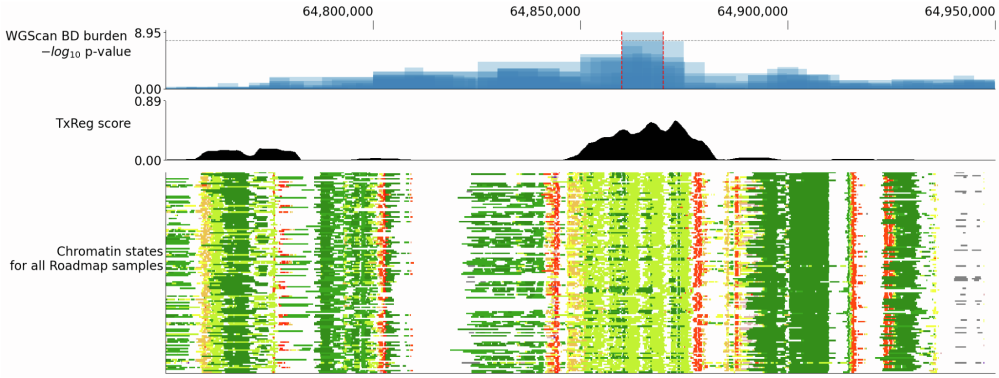

B

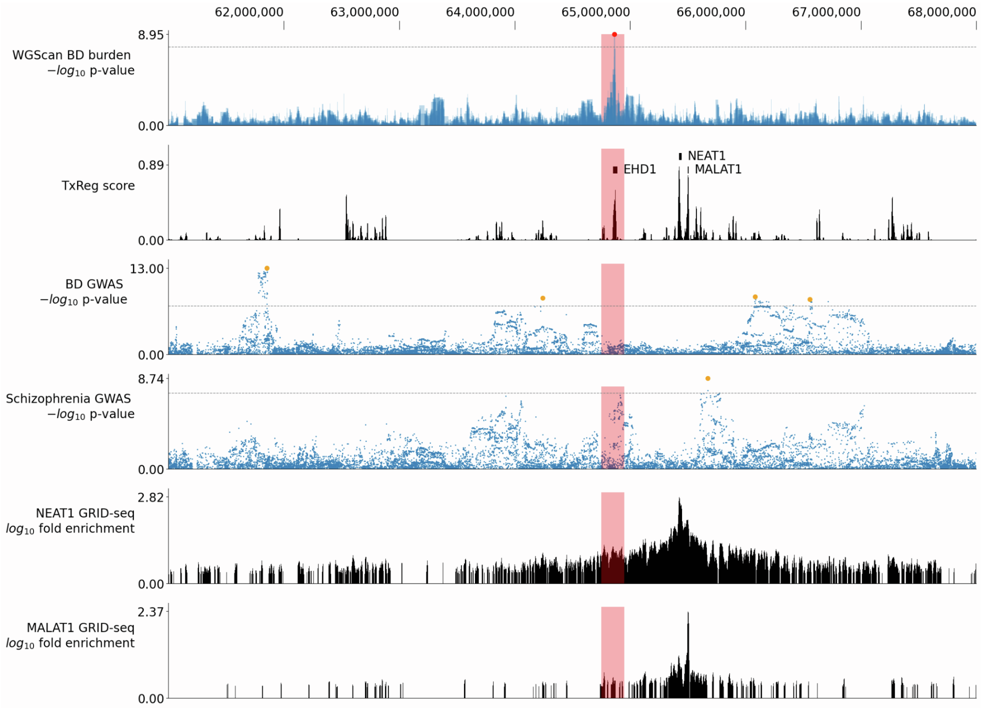

C

State	% of assignments in lead 10kb window	% 10kb windows >= frequency than lead
TxReg	**44.54**	**0.02**
TxEnh5'	29.61	0.08
TxEnhW	5.45	3.38
Tx5'	16.03	4.32
Tx	2.24	4.50
PromD2	0.09	6.89
Tx3'	0.13	17.40
EnhAF	0.02	23.17
TxWk	1.15	27.46
EnhAc	0.09	34.86
EnhA1	0.02	35.99
EnhA2	0.08	37.48
EnhWk2	0.19	50.73

Figure S14. Genomic region surrounding the significant window from WGScan BD burden analysis. (**A**) A smaller region surrounding the significant window. Top: windows tested by WGScan shown as bars with height corresponding to their -log_{10} p-values.

The significant window (chr11: 64,859,972-64,869,939) is indicated between the red dashed lines. Grey dashed line indicates the Bonferroni-corrected p-value threshold based on WGScan's estimated number of effective tests (0.05 / 2,463,399 = 2.0×10^{-8}). Middle: average TxReg score (computed as the percentage among all 127 Roadmap cell & tissue types that were assigned to the TxReg state) across 200bp bins of a 10kb window around the position. Bottom: chromatin state assignments using the 12-mark 25-state imputation-based ChromHMM annotations[8]. (**B**) A larger region surrounding the significant window (indicated as red boxes). From top to bottom: WGScan windows (same as in (**A**)), TxReg score (same as in (**A**)), GWAS summary statistics from the PGC BD study[5] and the PGC SZ study[6] with genome-wide significant loci shown as orange markers and genome-wide significance threshold shown as grey dashed lines, GRID-seq signal tracks for NEAT1 and MALAT1 on the MM.1S cell line[9]. (**C**) Percentages of 200bp bins assigned to each of the chromatin states (12-mark, 25-state ChromHMM model)[7,8] within the 10kb significant window, aggregated over 127 cell and tissue types, and the percentages of all 10kb windows throughout the genome with greater assignments.

TOPMed Study Short Acknowledgements with dbGaP accession numbers

Genome Sequencing for "NHLBI TOPMed: Cleveland Family Study - WGS Collaboration" (phs000954) was performed at NWGC (3R01HL098433-05S1).

Genome Sequencing for "NHLBI TOPMed: New Approaches for Empowering Studies of Asthma in Populations of African Descent - Barbados Asthma Genetics Study (BAGS)" (phs001143) was performed at Illumina (3R01HL104608-04S1).

Genome Sequencing for "NHLBI TOPMed: Genetic Epidemiology of COPD Study" (phs000951) was performed at NWGC and Broad Genomics (3R01HL089856-08S1, HHSN268201500014C, HHSN268201500014C).

Genome Sequencing for "NHLBI TOPMed: Multi-Ethnic Study of Atherosclerosis (MESA and MESA AA_CAC) "(phs001416.v1.p1) was performed at the Broad Institute of MIT and Harvard (3U54HG003067-13S1).

Genome Sequencing for "NHLBI TOPMed: Atherosclerosis Risk in Communities Study VTE cohort" (phs001211) was performed at Baylor (3U54HG003273-12S2 / HHSN268201500015C).

Genome Sequencing for "NHLBI TOPMed: Women's Health Initiative" (phs001237) was performed at Broad Genomics (HHSN268201500014C).

Genome Sequencing for "NHLBI TOPMed: Hypertension Genetic Epidemiology Network" (phs001293) was performed at NWGC (3R01HL055673-18S1).

Genome Sequencing for "NHLBI TOPMed: Sarcoidosis in African Americans" (phs001207) was performed at Baylor (3R01HL113326-04S1).

Genome Sequencing for "NHLBI TOPMed: Mount Sinai BioMe Biobank" (phs001644) was performed at Baylor (HHSN268201600033I).

Genome Sequencing for "NHLBI TOPMed: Coronary Artery Risk Development in Young Adults" (phs001612) was performed at Baylor (HHSN268201600033I).

TOPMed Study-specific Acknowledgements

Cleveland Family Study – WGS Collaboration (CFS)

The Cleveland Family Study has been supported in part by National Institutes of Health grants [R01-HL046380, KL2-RR024990, R35-HL135818, and R01-HL113338].

New Approaches for Empowering Studies of Asthma in Populations of African Descent - Barbados Asthma Genetics Study (BAGS)

We gratefully acknowledge the contributions of Pissamai and Trevor Maul, Paul Levett, Anselm Hennis, P. Michele Lashley, Raana Naidu, Malcolm Howitt and Timothy Roach, and the numerous health care providers, and community clinics and co-investigators who assisted in the phenotyping and collection of DNA samples, and the families and patients for generously donating DNA samples to the Barbados Asthma Genetics Study (BAGS). Funding for BAGS was provided by National Institutes of Health (NIH) R01HL104608, R01HL087699, and HL104608 S1.

Genetic Epidemiology of COPD Study (COPDGene)

The COPDGene study (NCT00608764) is supported by grants from the National Heart, Lung and Blood Institute (NHLBI; U01HL089897 and U01HL089856), by National Institutes of Health contract 75N92023D00011, and by the COPD Foundation through contributions made to an Industry Advisory Committee that has included AstraZeneca, Bayer Pharmaceuticals, Boehringer-Ingelheim, Genentech, GlaxoSmithKline, Novartis, Pfizer and Sunovion. A full listing of COPDGene investigators can be found at: http://www.copdgene.org/directory

Multi-Ethnic Study of Atherosclerosis (MESA & MESA AA_CAC)

Whole genome sequencing (WGS) for the Trans-Omics in Precision Medicine (TOPMed) program was supported by the National Heart, Lung and Blood Institute (NHLBI). WGS for "NHLBI TOPMed: Multi-Ethnic Study of Atherosclerosis (MESA)" (phs001416.v3.p1) was performed at the Broad Institute of MIT and Harvard (3U54HG003067-13S1). The MESA projects are conducted and supported by the National Heart, Lung, and Blood Institute (NHLBI) in collaboration with MESA investigators. Support for the Multi-Ethnic Study of Atherosclerosis (MESA) projects are conducted and supported by the National Heart, Lung, and Blood Institute (NHLBI) in collaboration with MESA investigators. Support for MESA is provided by contracts 75N92020D00001, HHSN268201500003I, N01-HC-95159, 75N92020D00005, N01-HC-95160, 75N92020D00002, N01-HC-95161, 75N92020D00003, N01-HC-95162, 75N92020D00006, N01-HC-95163, 75N92020D00004, N01-HC-95164, 75N92020D00007, N01-HC-95165, N01-HC-95166, N01-HC-95167, N01-HC-95168, N01-HC-95169, UL1-TR-000040, UL1-TR-001079, UL1-TR-001420, UL1TR001881, DK063491, and R01HL105756. The authors thank the other investigators, the staff, and the participants of the MESA study for their valuable contributions. A full list of participating MESA investigators and institutes can be found at http://www.mesa-nhlbi.org.

Atherosclerosis Risk in Communities study (ARIC VTE)

The Atherosclerosis Risk in Communities study has been funded in whole or in part with Federal funds from the National Heart, Lung, and Blood Institute, National Institutes of Health, Department of Health and Human Services, under Contract nos. (75N92022D00001, 75N92022D00002, 75N92022D00003, 75N92022D00004, 75N92022D00005). The authors thank the staff and participants of the ARIC study for their important contributions.

Whole genome sequencing (WGS) for the Trans-Omics in Precision Medicine (TOPMed) program was supported by the National Heart, Lung and Blood Institute (NHLBI). WGS for "NHLBI TOPMed: Atherosclerosis Risk in Communities (ARIC)" (phs001211) was performed at the Baylor College of Medicine Human Genome Sequencing Center (HHSN268201500015C and 3U54HG003273-12S2) and the Broad Institute for MIT and Harvard (3R01HL092577- 06S1).

Women's Health Initiative (WHI)

The WHI program is funded by the National Heart, Lung, and Blood Institute, National Institutes of Health, U.S. Department of Health and Human Services through contracts 75N92021D00001, 75N92021D00002, 75N92021D00003, 75N92021D00004, 75N92021D00005.

Hypertension Genetic Epidemiology Network (HyperGen)

The HyperGEN Study is part of the National Heart, Lung, and Blood Institute (NHLBI)

Family Blood Pressure Program; collection of the data represented here was supported by grants U01 HL054472 (MN Lab), U01 HL054473 (DCC), U01 HL054495 (AL FC), and U01 HL054509 (NC FC). The HyperGEN: Genetics of Left Ventricular Hypertrophy Study was supported by NHLBI grant R01 HL055673 with whole-genome sequencing made possible by supplement -18S1.

Genetics of Sarcoidosis in African Americans (Sarcoidosis)

National Institutes of Health (R01HL113326, P30 GM110766-01)

Mount Sinai BioMe Biobank (BioMe)

The Mount Sinai BioMe Biobank has been supported by The Andrea and Charles Bronfman Philanthropies and in part by Federal funds from the NHLBI and NHGRI (U01HG00638001; U01HG007417; X01HL134588). We thank all participants in the Mount Sinai Biobank. We also thank all our recruiters who have assisted and continue to assist in data collection and management and are grateful for the computational resources and staff expertise provided by Scientific Computing at the Icahn School of Medicine at Mount Sinai.

Coronary Artery Risk Development in Young Adults (CARDIA)

The Coronary Artery Risk Development in Young Adults Study (CARDIA) is conducted and supported by the National Heart, Lung, and Blood Institute (NHLBI) in collaboration with the University of Alabama at Birmingham (75N92023D00002 & 75N92023D00005), Northwestern University (75N92023D00004), University of Minnesota

(75N92023D00006), and Kaiser Foundation Research Institute (75N92023D00003). CARDIA was also partially supported by the Intramural Research Program of the National Institute on Aging (NIA) and an intra-agency agreement between NIA and NHLBI (AG0005).

TOPMed Neurocognitive Working Group

Last Name	First Name	Affiliation
Almeida	Marcio	University of Texas Rio Grande Valley School of Medicine
Ament	Seth	University of Maryland
Ammous	Farah	University of Michigan
Arnett	Donna	University of South Carolina
Becker	Diane	Johns Hopkins University
Bis	Joshua	University of Washington
Blue	Elizabeth	University of Washington
Boerwinkle	Eric	University of Texas Health at Houston
Bressler	Jan	University of Texas Health at Houston
Cammann	Davis	University of Nevada Las Vegas
Chaar	Dima	University of Michigan
Chen	Jingchun	University of Nevada Las Vegas
Clarkson-Townsend	Danielle	Brigham & Women's Hospital
Coresh	Josef	Johns Hopkins University

DeStefano	Anita	Boston University
Deters	Kacie	University of California, Los Angeles
Ding	Jingzhong	Wake Forest Baptist Health
Fardo	David	University of Kentucky
Fitzpatrick	Annette	University of Washington
Fornage	Myriam	University of Texas Health at Houston
French	Jennifer	University of Maryland
Glahn	David	Boston Children's Hospital, Harvard Medical School
Gonzalez	Hector	University of California, San Diego
Granot-Hershkovitz	Einat	Brigham & Women's Hospital
Hanly	Patrick	University of Calgary
Hayden	Kathleen	Wake Forest Baptist Health
Heckbert	Susan	University of Washington
Horvath	Steve	University of California, Los Angeles
Hoth	Karin	University of Iowa
Hughes	Timothy	Wake Forest Baptist Health

Jaiswal	Sidd	Stanford University
Jian	Xueqiu	University of Texas Health at San Antonio
Katsumata	Yuriko	University of Kentucky
Kho	Minjung	University of Michigan
Kooperberg	Charles	Fred Hutchinson Cancer Research Center
Launer	Lenore	National Institute on Aging
Lin	Honghuang	Boston University
Litkowski	Elizabeth	University of Colorado Anschutz Medical Campus
Longstreth	Will	University of Washington
Mayeux	Richard	Columbia University
Mikulla	Julie	National Heart, Lung, and Blood Institute
Misra	Biswapriya	Wake Forest Baptist Health
Mosley	Thomas	University of Mississippi
Nyquist	Paul	Johns Hopkins University
O'Connell	Jeff	University of Maryland
Olivier	Michael	Wake Forest Baptist Health
Panyard	Daniel	Stanford University

Peloso	Gina	Boston University
Perry	James	University of Maryland
Psaty	Bruce	University of Washington
Purcell	Shaun	Mass General Brigham
Raffield	Laura	University of North Carolina
Reiner	Alex	Fred Hutchinson Cancer Research Center, University of Washington
Rotter	Jerome	Lundquist Institute
Sargurupremraj	Muralidharan	University of Texas Health at San Antonio
Sarnowski	Chloé	Boston University, University of Texas Health at Houston
Satizabal	Claudia	Boston University
Schellenberg	Gerard	University of Pennsylvania
Seo	Jungkyun	University of North Carolina
Seshadri	Sudha	University of Texas Health at San Antonio
Shade	Lincoln	University of Kentucky
Short	Meghan	Boston University
Simino	Jeannette	University of Mississippi

Smith	Jennifer	University of Michigan
Smoller	Sylvia	Albert Einstein College of Medicine
Snively	Beverly	Wake Forest Baptist Health
Soemedi	Rachel	Brigham & Women's Hospital
Sokolow	Sophie	University of California, Los Angeles
Thornton	Timothy	University of Washington
Vivek	Sithara	University of Minnesota
Wood	Alexis	Baylor College of Medicine
Yanek	Lisa	Johns Hopkins University
Yang	Qiong	Boston University
Yu	Miao	University of Michigan
Zare	Habil	University of Texas Health at San Antonio
Zhao	Wei	University of Michigan

Supplemental References

1. Wei, S., Xu, Y., Shi, H., Wong, S.-H., Han, W., Talbot, K., Hong, W., and Ong, W.-Y. (2010). EHD1 is a synaptic protein that modulates exocytosis through binding to snapin. Mol. Cell. Neurosci. *45*, 418–429. https://doi.org/10.1016/j.mcn.2010.07.014.
2. Yap, C.C.C., Lasiecka, Z.M., Caplan, S., and Winckler, B. (2010). Alterations of EHD1/EHD4 Protein Levels Interfere with L1/NgCAM Endocytosis in Neurons and Disrupt Axonal Targeting. J. Neurosci. *30*, 6646–6657. https://doi.org/10.1523/JNEUROSCI.5428-09.2010.
3. Kataoka, M., Matoba, N., Sawada, T., Kazuno, A.-A., Ishiwata, M., Fujii, K., Matsuo, K., Takata, A., and Kato, T. (2016). Exome sequencing for bipolar disorder points to roles of de novo loss-of-function and protein-altering mutations. Mol. Psychiatry *21*, 885–893. https://doi.org/10.1038/mp.2016.69.
4. Nakamura, T., Nakajima, K., Kobayashi, Y., Itohara, S., Kasahara, T., Tsuboi, T., and Kato, T. (2021). Functional and behavioral effects of de novo mutations in calcium-related genes in patients with bipolar disorder. Hum. Mol. Genet. *30*, 1851–1862. https://doi.org/10.1093/hmg/ddab152.
5. Mullins, N., Forstner, A.J., O'Connell, K.S., Coombes, B., Coleman, J.R.I., Qiao, Z., Als, T.D., Bigdeli, T.B., Børte, S., Bryois, J., et al. (2021). Genome-wide association study of more than 40,000 bipolar disorder cases provides new insights into the underlying biology. Nat. Genet. *53*, 817–829. https://doi.org/10.1038/s41588-021-00857-4.
6. Trubetskoy, V., Pardiñas, A.F., Qi, T., Panagiotaropoulou, G., Awasthi, S., Bigdeli, T.B., Bryois, J., Chen, C.-Y., Dennison, C.A., Hall, L.S., et al. (2022). Mapping genomic loci implicates genes and synaptic biology in schizophrenia. Nature *604*, 502–508. https://doi.org/10.1038/s41586-022-04434-5.
7. Ernst, J., and Kellis, M. (2012). ChromHMM: automating chromatin-state discovery and characterization. Nat. Methods *9*, 215–216. https://doi.org/10.1038/nmeth.1906.
8. Ernst, J., and Kellis, M. (2015). Large-scale imputation of epigenomic datasets for systematic annotation of diverse human tissues. Nat. Biotechnol. *33*, 364–376. https://doi.org/10.1038/nbt.3157.
9. Li, X., Zhou, B., Chen, L., Gou, L.-T., Li, H., and Fu, X.-D. (2017). GRID-seq reveals the global RNA–chromatin interactome. Nat. Biotechnol. *35*, 940–950. https://doi.org/10.1038/nbt.3968.
10. Yang, S., Lim, K.-H., Kim, S.-H., and Joo, J.-Y. (2021). Molecular landscape of long noncoding RNAs in brain disorders. Mol. Psychiatry *26*, 1060–1074. https://doi.org/10.1038/s41380-020-00947-5.
11. Srinivas, T., Mathias, C., Oliveira-Mateos, C., and Guil, S. (2023). Roles of lncRNAs in brain development and pathogenesis: Emerging therapeutic opportunities. Mol. Ther. *31*, 1550–1561. https://doi.org/10.1016/j.ymthe.2023.02.008.
12. Katsel, P., Roussos, P., Fam, P., Khan, S., Tan, W., Hirose, T., Nakagawa, S., Pletnikov, M.V., and Haroutunian, V. (2019). The expression of long noncoding RNA NEAT1 is reduced in schizophrenia and modulates oligodendrocytes transcription. Npj Schizophr. *5*, 3. https://doi.org/10.1038/s41537-019-0071-2.
13. Shirvani Farsani, Z., Zahirodin, A., Ghaderian, S.M.H., Shams, J., and Naghavi Gargari, B. (2020). The role of long non-coding RNA MALAT1 in patients with bipolar disorder. Metab. Brain Dis. *35*, 1077–1083. https://doi.org/10.1007/s11011-020-00580-9.
