# Supplementary material for: Whole-genome sequence-based association analysis of African American individuals with bipolar disorder and schizophrenia
Source: HGG Adv. 2025 Aug 30;7(1):100499. doi: 10.1016/j.xhgg.2025.100499 (PMC12494821; doi:10.1016/j.xhgg.2025.100499)
Supplement: Document S2. Article plus supplemental information [file mmc3.pdf]

HGG
Advances

ARTICLE

Whole-genome sequence-based association analysis of African American individuals with bipolar disorder and schizophrenia

Runjia Li,[1,2,41] Sarah A. Gagliano Taliun,[3,4,5,6,41,42] Kevin Liao,[3,7] Matthew Flickinger,[3] Janet L. Sobell,[8] Giulio Genovese,[9] Adam E. Locke,[3,10] Rebeca Rothwell Chiu,[3] Jonathon LeFaive,[3] Jiongming Wang,[3] Taylor Martins,[3,11] Sinéad Chapman,[9] Anna Neumann,[9] Robert E. Handsaker,[9,12] Donna K. Arnett,[13] Kathleen C. Barnes,[14] Eric Boerwinkle,[15] David Braff,[16,17] Brian E. Cade,[18] Myriam Fornage,[19] Richard A. Gibbs,[20] Karin F. Hoth,[21] Lifang Hou,[22] Charles Kooperberg,[23] Ruth J.F. Loos,[24]

(Author list continued on next page)

Summary

In studies of individuals of primarily European genetic ancestry, common and low-frequency variants and rare coding variants have been found to be associated with the risk of bipolar disorder (BD) and schizophrenia (SZ). However, less is known for individuals of other genetic ancestries or the role of rare non-coding variants in BD and SZ risk. We performed whole-genome sequencing (~27X) of African American individuals: 1,598 with BD, 3,295 with SZ, and 2,651 unaffected controls (InPSYght study). We increased power by incorporating 14,812 jointly called psychiatrically unscreened ancestry-matched controls from the Trans-Omics for Precision Medicine (TOPMed) Program for a total of 17,463 controls (~37X). To identify variants and sets of variants associated with BD and/or SZ, we performed single-variant tests, gene-based tests for singleton protein truncating variants, and rare and low-frequency variant annotation-based tests with conservation and universal chromatin states and sliding windows. We found suggestive evidence of the association of BD with single variants on chromosome 18 and of lower BD risk associated with rare and low-frequency variants on chromosome 11 in a region with multiple BD genome-wide association study loci, using a sliding window approach. We also found that chromatin and conservation state tests can be used to detect differential calling of variants in controls sequenced at different centers and to assess the effectiveness of sequencing metric covariate adjustments. Our findings reinforce the need for continued whole-genome sequencing in additional samples of African American individuals and more comprehensive functional annotation of non-coding variants.

Introduction

Severe mental illnesses, including bipolar disorder (BD) and schizophrenia (SZ), are debilitating disorders that affect millions of people worldwide. BD and SZ encompass a wide range of shared symptoms, including recurrent episodes of psychosis, large mood swings, depression, and cognitive impairment. Both disorders are significantly associated with risk of suicide and increased all-cause mortality rate.[1] Heritability estimates from family studies

[1]Bioinformatics Interdepartmental Program, University of California, Los Angeles, Los Angeles, CA, USA; [2]Department of Biological Chemistry, University of California, Los Angeles, Los Angeles, CA, USA; [3]Department of Biostatistics and Center for Statistical Genetics, University of Michigan, Ann Arbor, MI, USA; [4]Department of Neurosciences, Université de Montréal, Montreal, QC, Canada; [5]Department of Medicine, Université de Montréal, Montreal, QC, Canada; [6]Montreal Heart Institute Research Centre, Montreal, QC, Canada; [7]Genomics plc, Oxford, UK; [8]Department of Psychiatry and Behavioral Sciences, Keck School of Medicine, University of Southern California, Los Angeles, CA, USA; [9]Stanley Center for Psychiatric Research, Broad Institute of MIT and Harvard, Cambridge, MA, USA; [10]Regeneron Genetics Center, Tarrytown, NY, USA; [11]Arizona Department of Health Services, Phoenix, AZ, USA; [12]Department of Genetics, Harvard Medical School, Boston, MA, USA; [13]Department of Epidemiology and Biostatistics, Arnold School of Public Health, University of South Carolina, Columbia, SC, USA; [14]Departments of Medicine and Epidemiology, University of Colorado Anschutz Medical Campus, Aurora, CO, USA; [15]Department of Epidemiology, University of Texas Health Science Center at Houston School of Public Health, Houston, TX, USA; [16]Department of Psychiatry, University of California, San Diego, San Diego, CA, USA; [17]VA San Diego Healthcare System, San Diego, CA, USA; [18]Department of Medicine, Brigham and Women's Hospital, Boston, MA, USA; [19]Brown Foundation Institute of Molecular Medicine, McGovern Medical School, University of Texas Health Science Center at Houston, Houston, TX, USA; [20]Baylor College of Medicine Human Genome Sequencing Center, Department of Molecular and Human Genetics, Houston, TX, USA; [21]Department of Psychiatry, University of Iowa, Iowa City, IA, USA; [22]Department of Preventive Medicine, Northwestern University, Chicago, IL, USA; [23]Division of Public Health Sciences, Fred Hutchinson Cancer Center, Seattle, WA, USA; [24]Charles Bronfman Institute for Personalized Medicine, Icahn School of Medicine at Mount Sinai, New York, NY, USA; [25]Oklahoma Medical Research Foundation, Oklahoma City, OK, USA; [26]Department of Biostatistics and Bioinformatics, Emory University, Atlanta, GA, USA; [27]Department of Epidemiology, University of Washington, Seattle, WA, USA; [28]Department of Genome Sciences, University of Virginia, Charlottesville, VA, USA; [29]Institute for Translational Genomics and Population Sciences, Department of Pediatrics, Lundquist Institute for Biomedical Innovation at Harbor-UCLA Medical Center, Torrance, CA, USA; [30]Illumina Laboratory Services, Illumina Inc., 5200 Illumina Way, San Diego, CA, USA; [31]Institute for Genomics in Health (IGH), SUNY Downstate Health Sciences University, Brooklyn, NY, USA; [32]Department of Psychiatry and Behavioral Sciences, SUNY Downstate Health Sciences University, Brooklyn, NY, USA; [33]VA New York Harbor Healthcare System, Brooklyn, NY, USA; [34]Genomics Platform, Broad Institute of MIT and Harvard, Cambridge, MA, USA; [35]Department of Genetics, Harvard Medical School, 77 Avenue Louis Pasteur, NRB 260, Boston, MA, USA; [36]Department of Psychiatry, Rutgers

(Affiliations continued on next page)

© 2025 The Author(s). Published by Elsevier Inc. on behalf of American Society of Human Genetics.
This is an open access article under the CC BY license (http://creativecommons.org/licenses/by/4.0/).

Ginger A. Metcalf,[20] Courtney G. Montgomery,[25] Alanna C. Morrison,[15] Zhaohui S. Qin,[26] Susan Redline,[18] Alexander P. Reiner,[27] Stephen S. Rich,[28] Jerome I. Rotter,[29] Kent D. Taylor,[29] Karine A. Viaud-Martinez,[30] National Heart, Lung, and Blood Institute Trans-Omics for Precision Medicine (TOPMed) Consortium,[43], Genomic Psychiatry Cohort Investigators[43] Tim B. Bigdeli,[31,32,33] Stacey Gabriel,[34] Sebastian Zollner,[3] Albert V. Smith,[3] Goncalo Abecasis,[3,10] Steve A. McCarroll,[9,35] Michele T. Pato,[36] Carlos N. Pato,[36] Michael Boehnke,[3] James Knowles,[37] Hyun Min Kang,[3] Roel A. Ophoff,[38,42] Jason Ernst,[2,39,40,42,*] and Laura J. Scott[3,42,44,*]

range from 60% to 85% for BD[2] and 60% to 80% for SZ.[3] Notably, there is considerable overlap in the underlying genetics of BD and SZ,[4] and genetic correlations are estimated to be as high as 0.68 at the common variant level.[5] Uncovering the genetic factors contributing to these disorders could lead to a deeper understanding of disease etiology and improved treatment options.

Hundreds of independent susceptibility loci for BD and SZ have been identified through large-scale genome-wide association studies (GWASs) by focusing on common and low-frequency alleles.[6–13] An SZ case-control GWAS of European and east Asian ancestry individuals (Psychiatric Genomics Consortium, 76,755 individuals with SZ and 243,649 controls) identified 287 distinct loci, implicating genes associated with neurodevelopmental disorders and with brain-specific expression.[13] A BD GWAS of European ancestry (41,917 BD cases and 371,549 controls) identified 64 distinct loci and significant enrichment of association signals within genes belonging to neuronal and synaptic pathways and targets for existing BD medications.[4] Of the BD-associated variants, 17 were also associated with SZ. For both the BD and SZ GWASs, measured common variants are estimated to account for a modest portion of disease heritability (18.6% for BD[4] and 24% for SZ[14]).

Whole-exome sequencing (WES) detects coding variants across the allele frequency spectrum. The Schizophrenia Exome Sequencing Meta-Analysis (SCHEMA) Consortium produced an SZ case-control WES study (SCHEMA) of multi-ancestry individuals (24,248 SZ cases and 97,322 controls), which identified enrichment of ultra-rare coding variants in 10 genes in individuals with SZ[15]; 2 of the genes were also implicated by common-variant GWAS.[13] A WES BD case-control study (BipEx) of European ancestry individuals (13,933 BD cases and 14,422 controls) found that compared to controls, individuals with BD were enriched for ultra-rare protein truncating variants (PTVs) in constrained genes (probability of being loss-of-function intolerant [pLI] $\geq$ 0.9). When the BipEx and SCHEMA results were combined, *AKAP11* was identified as a risk gene.[16] These results identified ultra-rare coding variants as contributing to BD and SZ risk and suggested overlap between BD and SZ risk at both the rare and common variant levels.

Compared to common and exonic variants, less is known about the role of rare non-coding variants in SZ and BD. Whole-genome sequencing (WGS) allows detection of coding and non-coding variants across the allele frequency spectrum. Studies using WGS to investigate SZ or BD have generally been limited for non-coding variant analysis by sample sizes of no more than a few hundred individuals, often in family-based designs.[17–23] One SZ WGS case-control study of Swedish samples (1,162 SZ cases and 936 controls) found association between SZ and structural variants at topologically associated domain boundaries but did not find significant differential burden of non-coding single-nucleotide variants (SNVs) and insertion or deletion polymorphisms (indels) between SZ cases and controls across a variety of biological groupings.[24]

To date, genomic studies of psychiatric disorders and many complex human diseases and traits have overwhelmingly been composed of individuals of European genetic ancestry.[25,26] Although there has been progress in increasing the representation of ancestral backgrounds of individuals included in GWASs, notably in east Asians, the available data do not comprehensively represent individuals in the United States or the world.[27] This impedes the discovery of genes and mechanisms that might be uncovered from the broader spectrum of variations across different ancestries. The use of European ancestry BD polygenic risk scores (PRSs) to predict disease risk across ancestries also has the potential to create health inequities. For example, PRSs from European ancestry GWASs predict a much smaller proportion of disease in east Asian and African American ancestry samples than in European ancestry samples.[4] Increasingly, efforts are under way to assess the influence of genetic variation on complex traits in individuals of non-European ancestry.[28–30] Genetic studies of mental health disorders that include WGS for

University, New Brunswick, NJ, USA; [37]Human Genetics Institute of New Jersey, Rutgers University, New Brunswick, NJ, USA; [38]Center for Neurobehavioral Genetics, Semel Institute for Neuroscience and Human Behavior, University of California, Los Angeles, Los Angeles, CA, USA; [39]Department of Computational Medicine, University of California, Los Angeles, Los Angeles, CA, USA; [40]Computer Science Department, University of California, Los Angeles, Los Angeles, CA, USA
[41]These authors contributed equally
[42]Senior author
[43]Further details can be found in the supplemental information
[44]Lead contact
*Correspondence: jason.ernst@ucla.edu (J.E.), ljst@umich.edu (L.J.S.)
https://doi.org/10.1016/j.xhgg.2025.100499.

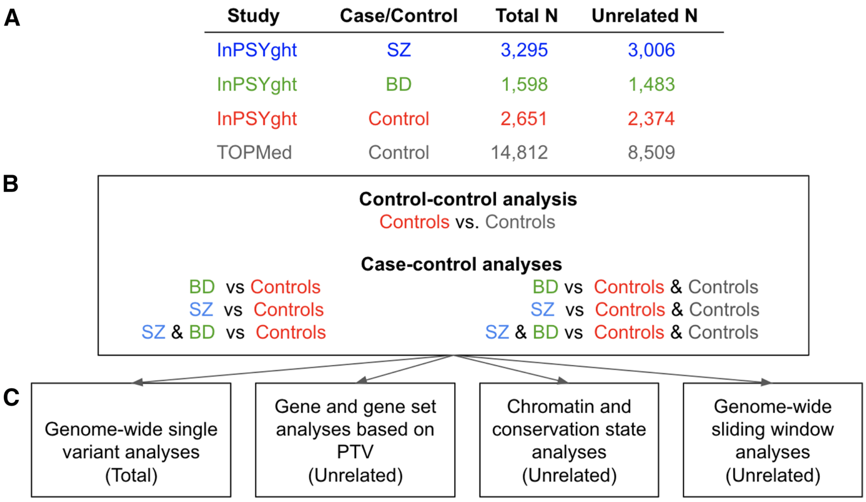

Figure 1. Study overview
(A) Number of total and unrelated study participants for each case or control group.
(B) Seven analysis groups: control-control and case-control.
(C) Four analysis types (total or unrelated samples used in analysis). PTV, protein truncating variant.

individuals of diverse genetic ancestries will allow us to better address the disparities in diagnosis and treatment.

We examined the role of SNVs and short indels in BD and SZ susceptibility in African American individuals in a sample of 7,544 individuals (with 1,598 BD cases, 3,295 SZ cases, and 2,651 controls without SZ or BD) and an additional 14,812 phenotypically unscreened ancestry-matched individuals from the National Heart, Lung, and Blood Institute (NHLBI) Trans-Omics for Precision Medicine (TOPMed) program as external controls.[30] Overall, we found suggestive evidence of single-variant BD association on chromosome 18. We observed that chromatin and conservation state burden tests were a sensitive way to assess the comparability of sequencing/calling between two WGS sample sets. We also found suggestive evidence of association of a chromosome 11 region sliding window located among multiple previously reported BD GWAS loci.[4]

Subjects and methods

Figure 1 contains an overview of the study design, samples, and analytical approaches.

InPSYght study sample

We selected individuals for deep whole-genome sequencing (WGS) from US-based case-control studies of African American individuals as part of the Whole Genome Sequence for Psychiatric Disorders (WGSPD) Consortium.[31] We refer to this study as the InPSYght study. We use the term "African American" to denote individuals who self-identified as African American on study forms; this term (which may have been one of a limited number of choices to describe African ancestry) can include individuals who, among others, are descendants of enslaved individuals or are more recent immigrants to the United States from African and other countries. The InPSYght study is composed of participants from the Genomic Psychiatry Cohort (GPC),[32,33] the Consortium on the Genetics of SZ (COGS),[34] the Bipolar Genome Study (BIGS),[35] Lithium Treatment Moderate Dose Use Study (LiTMUS),[36] and the Systematic Treatment Enhancement Program for Bipolar Disorder (STEP-BD) studies.[37] All DNA samples were obtained from the National Institute of Mental Health (NIMH) Repository and Genomics Resource (Table S1). The array-based genotypes were obtained from the NIMH Repository or from the originating study. From these studies, we selected individuals with self-reported African/African American ancestry, with the goal of sequencing individuals who were more likely to have primarily European and African ancestry admixture. We used ADMIXTURE[38] to estimate the percentage of African genetic ancestry in each individual using study sample genotype array data and the 1000 Genomes[39] ancestry super-populations Admixed Americans (AMR), African (AFR), European (EUR), South Asian (SAS), and East Asian (EAS) as reference populations. We used a cutpoint of >25% estimated percentage global African genetic ancestry for sample inclusion in sequencing. All individuals designated as cases fulfilled the *Diagnostic and Statistical Manual of Mental Disorders*-IV criteria for SZ or BD. InPSYght controls (all from the GPC study) were included if they had no personal or family history of SZ or BD and if they did not have unipolar depression (from screening questions). Details of the recruitment strategies and instruments used for diagnosis are provided in the referenced publications for each cohort. All participants provided informed consent. The IRB of the University of Michigan reviewed the details of the study and determined it was "not regulated".

TOPMed external controls

To increase statistical power to detect variants associated with BD and SZ, we selected as external controls previously whole-genome sequenced self-identified African American individuals from the NHLBI TOPMed project (hereafter referred to as TOPMed controls). TOPMed studies (case-control or family-based studies) are focused on heart, lung, blood and sleep disorders, and inclusion was not predicated on information about mental health.[30] Details of the IRB approval for the studies in TOPMed are available elsewhere (See Section 4 in Taliun et al.[30]). We considered for inclusion individuals from 11 studies who agreed to the use of their samples as controls (Table S2). We considered for inclusion individuals with general research use (GRU) consent or consent for health/medical/biomedical purposes. Further inclusion/exclusion criteria are described below.

WGS of InPSYght and TOPMed individuals

InPSYght: Individuals were whole-genome sequenced (mean ± SD depth 26.8 ± 5.5) in seven batches at the Broad Institute on an Illumina HiSeq X10 instrument. The first WGS batch ($n = 231$ samples) was performed with PCR-based library preparation; the remaining batches were completed PCR-free. All

sequencing was paired end with a 151-nt read length. Individuals with SZ, with BD, and control individuals were included in each sequencing batch, except for the PCR-amplified batch, which did not include individuals with BD (Table S3). Case and internal control samples were intermixed within each batch to help avoid sub-batch effects.

TOPMed individuals were whole-genome sequenced at five sequencing centers as previously described[30] (Table S2), using paired-end sequencing and read length of 150 bp. The 14,812 samples used as controls had a mean sequencing depth of 36.8 ± 4.7.

Joint InPSYght and TOPMed variant discovery, genotype calling, and quality control

We used jointly called genotypes on human genome build version GRCh38 for bi-allelic SNVs and short indels from TOPMed Freeze 9[30] using the GotCloud/vt pipeline.[38] We called genotypes for each individual on the autosomes and chromosome X on the across-individuals union of all variant sites. Genotypes for variants on the non-pseudo-autosomal region of the X chromosome were coded as 0 or 2 alleles for males, whereas those in the two PARs (PAR1 and PAR2, respectively) were coded 0, 1, or 2 alleles combining X and Y for males. We filtered variants based on outputs from a support vector machine (SVM) classifier based on inferred pedigree of related and duplicated individuals to calculate Mendelian consistency statistics and other features.[30,39]

Initial exclusions of individuals from analysis

For the InPSYght study, we excluded individuals with sex mismatches (self-reported sex disagreed with genetic sex) ($n = 20$), non-XX or XY sex karyotypes ($n = 17$), estimated DNA contamination >5% using verifyBamID2[40] ($n = 4$), or for whom <98% of sites were at a sequencing depth of ≥ 10 ($n = 14$) (see Table S4). For TOPMed, we excluded individuals with sex mismatches, non-XX or XY sex karyotypes, or with estimated DNA contamination >5%.[30]

Construction of PCs for inclusion of sequenced samples

To compute principal components (PCs) of genetic ancestry, we removed variants in high long-range linkage disequilibrium (LD) regions[41] and then pruned variants in PLINK/1.9[42] using –indep-pairwise flag and the following parameters: 500000 5 0.2. We computed PCs using the --pca flag within PLINK[42] and restricted variant selection to those present in the Human Genome Diversity Project (HGDP) reference panel.[43] These criteria resulted in 160,517 common (minor allele frequency [MAF] >5% in the InPSYght case-control + TOPMed control dataset) autosomal bi-allelic SNVs (from the WGS data). We retained InPSYght individuals who visually clustered in the first three PCs. Then, we retained TOPMed controls who visually clustered in the space occupied by the InPSYght individuals along the first three PCs (Figures S1A–S1D).

Identification of duplicated and related individuals

We estimated pairwise kinship among all individuals (InPSYght and TOPMed controls) using KING (kinship-based inference for GWASs).[44] We excluded 1 person per pair of monozygotic/duplicate genotyped individuals (17 duplicates within InPSYght, 140 duplicates within TOPMed, and 52 within InPSYght/TOPMed). For InPSYght/TOPMed pairs, the InPSYght sample was retained. To obtain unrelated individuals for variant aggregation tests, we randomly retained an individual from related pairs or groups (defined by individuals being related with at least one other person in the group) with kinship >0.0409 (third-degree relationship or higher), resulting in 4,489 cases (3,006 SZ and 1,483 BD), 2,374 InPSYght controls, and 8,509 TOPMed controls.

Construction of PCs for association analysis

We computed genetic PCs for use in analysis as described in the section *Construction of PCs for inclusion of sequenced samples* by using only the selected TOPMed and InPSYght individuals (Figures S1E–S1H).

Genetic ancestry estimation in InPSYght using WGS data

We re-estimated the global genetic ancestries for InPSYght samples using WGS data to use the same genotype source across studies. We used the supervised learning approach implemented in the software ADMIXTURE[45] trained on the 1000 Genomes Project phase 3[46] ancestry super-populations AMR, AFR, EUR, SAS, and EAS. To estimate sample ancestry, we used the same SNVs as for the PC analysis.

We further inferred finer grain genetic ancestry using as a reference individuals from the HGDP reference panel and using RFMix to estimate ancestry (run by chromosome and summed across chromosomes).[47] HGDP was chosen here in an attempt to characterize known high-levels of genetic diversity across African populations.[48] From the HGDP reference, we used 156 European individuals as a single group, and as separate groups we used between 8 and 27 individuals per population from the following 8 African populations: Bantu from South Africa, Bantu from Kenya, Biaka Pygmy, Mandenka, Mbuti Pygmy, Mozabite, San, and Yoruba.[49]

Genetic ancestry estimation in TOPMed using WGS data

Estimated genetic ancestry of TOPMed samples has been described previously.[30] In summary, local ancestry was inferred using RFMix version 2[47] with the following option: –node-size = 5. For reference haplotypes used in local ancestry inference, we obtained the HGDP reference panel[50] and processed the data according to Wang et al.,[51] giving 938 individuals and 639,958 autosomal SNVs. We then condensed the 53 populations in HGDP into 7 super-populations: (1) Sub-Saharan Africa ($n = 104$), (2) Central/South Asia ($n = 200$), (3) East Asia ($n = 229$), (4) Europe ($n = 154$), (5) Native America ($n = 63$), (6) Oceania ($n = 28$), and (7) Middle East ($n = 160$). After running RFMix, we summed up inferred local ancestry across all genetic windows of each individual to calculate global ancestry proportions, corresponding to the seven super-populations. Almost all selected TOPMed controls (n = 14,804, 99.9%) had >25% African estimated global ancestry (range: 26%–100%), except 8 (0.05%) selected controls with range of estimated African ancestry: 8%–24%.

Power calculations to detect single-variant associations

We calculated the odds ratio (OR) that would yield 80% power to detect variants associated with BD, SZ, or SZ + BD, using InPSYght + TOPMed samples as controls. We assumed a disease prevalence of 1% for BD and 1% for SZ and 2% for SZ + BD. We conservatively used approximate numbers of unrelated cases ($n = 1{,}500$ BD and $n = 3{,}000$ SZ) and controls ($n = 11{,}000$) present

in the case-control comparison group of interest in our calculations. As we were interested in the power to detect lower frequency variants, we assumed risk allele frequencies of 0.01 and 0.05, using standard assumptions of a multiplicative disease model on the OR scale, population-based controls, and genome-wide significance level of 5×10^{-9} to account for the testing of variants with MAF <0.05, using the Genetic Power Calculator.[52]

Genome-wide single-variant case-control association analysis

We tested for association of SZ and/or BD with each SNV or indel (minor allele count [MAC] >20 in the tested individuals) using SAIGE (version 0.42), which employs a mixed model to account for related individuals and uses saddlepoint approximation to account for case-control imbalance in estimation of significance (the estimations are stable down to an MAC of 20).[53] We used the reference genome allele as the reference allele in calculation of ORs. We performed a total of six case-control association analyses (Figure 1). We used as cases the SZ-only, BD-only, or combined SZ or BD InPSYght samples. We used as controls either only the InPSYght controls or the InPSYght + TOPMed controls. We chose to combine the SZ and BD cases based on previous evidence of substantial genetic correlation between the two disorders (based on common variants in European-ancestry populations).[54] We included as covariates genetic sex and the first 10 genetic PCs. We also included the sequencing batch as a covariate for InPSYght sample-only analyses. To assess potential differences between the two sets of controls (InPSYght and TOPMed controls), we performed an association analysis of InPSYght controls versus TOPMed controls. We controlled for multiple testing within an analysis group using $p < 5 \times 10^{-9}$ for genome-wide significance; we used $p < 5 \times 10^{-9}/7$ comparisons $= 7.1 \times 10^{-10}$ for a conservative genome-wide significance.

PTV singletons-based burden tests

We performed burden tests for protein truncating SNV or indels (PTV) singletons at both the gene level and the gene-set level. We restricted our analysis to KING-estimated unrelated samples (less than third-degree relationships, see above) consisting of 4,489 InPSYght BD and SZ cases, 2,374 InPSYght controls, and 8,509 TOPMed controls. We defined singleton variants based on the unrelated samples to avoid excluding variants that occurred multiple times in a single family. We annotated 61,732 singleton variants as PTVs using the following Ensembl Variant Effect Predictor categories: frameshift, stop gained, splice acceptor, and splice donor. We performed gene-level burden tests of SZ + BD cases versus InPSYght + TOPMed controls on the aggregated singleton PTVs within each gene, testing for association of PTV count with case status using RVTESTS.[55] To maximize power, we restricted testing to the genes with >10 PTV singletons (1,045 of 22,178 genes) and only tested SZ + BD cases versus InPSYght + TOPMed controls given the limited number of PTVs available per gene for testing. We included as covariates the first 10 genetic PCs, sex, and an individual's total number of singleton alleles. We applied a Bonferroni-corrected significance threshold of 0.05/1,045 (of genes with ≥10 PTV singletons) $= 4.8 \times 10^{-5}$. As a sensitivity analysis, we expanded singletons to include PTVs up to 1% MAF and repeated the gene-based testing. At a 1% MAF threshold, we tested all 22,178 genes and used a corrected significance threshold of 0.05/22,178 $= 2.3 \times 10^{-6}$).

In addition, among the 10 previously reported SZ associated genes from the SCHEMA Consortium study,[15] we tested the 6 genes with at least 1 PTV singleton in our study; all 6 genes had <10 PTV singletons. We note that the SCHEMA Consortium study includes the InPSYght SZ and control samples; thus, this is not an independent test but a test to see the contribution of the African American samples.

We performed gene set-level tests for the enrichment of PTV singletons within sets of genes previously associated with SZ. Given the higher singleton counts in gene set tests compared to individual genes, in addition to testing SZ + BD versus InPSYght + TOPMed controls as for individual genes, we tested SZ versus InPSYght + TOPMed controls. We tested three gene sets previously associated with SZ in more than one study: 1,423 postsynaptic density genes,[56,57] 784 FMR1 protein-associated (formerly named FMRP) genes,[58,59] and 3,063 constrained (pLI >0.90) genes.[59] In addition, we tested a gene set we constructed containing the 10 SCHEMA SZ-associated genes,[15] which as noted above has some overlapping samples with the samples for this study. For each gene set test, for each person we summed the PTV counts over all genes in the gene set and used RVTESTS[55] to test for association with case status as described for single-gene tests. We used a single analysis Bonferroni significance threshold of 0.05/3 = 0.017; in analysis where a result passed the single analysis threshold, we further evaluated the result using more stringent threshold of 0.05/(3 × 2 comparisons) = 0.0085 to account for multiple analyses.

Construction of sequencing metadata PCs

To control for potential sequencing batch effects within and across InPSYght and TOPMed, we constructed PCs based on a shared set of sample-level sequencing quality control metrics, including per-sample average depths and sample contamination levels (Table S5). We used the first four of these sequencing metadata PCs in the chromatin and conservation states analysis as they cumulatively explain 99.99% of the variance of the sequencing metadata PCs.

Test for case-control and InPSYght control-TOPMed control enrichment of rare and low-frequency variants in chromatin states and conservation states

We tested whether cases and controls exhibit differential enrichment of rare and low-frequency SNVs (MAF <0.05) for any class of genomic region defined based on chromatin or conservation states. Specifically, for the chromatin states, we used the universal ChromHMM[60] 100-chromatin state annotation of the human genome, which captures combinatorial and spatial patterns of chromatin marks over 1,000 epigenomic datasets from more than 100 cell and tissue types. The version of the annotations we used had been previously lifted over to human hg38 assembly from hg19.[60] For the conservation states, we used a ConsHMM[61] 100-conservation state annotation of the human genome defined directly in hg38, which captures combinatorial and spatial patterns of individual nucleotides aligning to and matching the human reference genome within a 100-way vertebrate sequence alignment.[61,62]

For the same set of unrelated samples as in the gene-based tests, we used SNVs with MAF <0.05, excluding variants overlapping ENCODE-excluded regions.[63] We annotated each variant with the ChromHMM and ConsHMM annotations described above. For each of the six case-control and InPSYght control/TOPMed

control comparisons described in the single-variant test section, we used logistic regression to test for association between the non-reference allele count (predictor) and case-control or control study status (outcome). We upweighted rarer variants with the beta function *beta*(MAF, 1, 25) (mirroring the default choice of WGScan[64]). We included as covariates the first 10 genetic PCs, sex, sequencing batch (for tests involving only InPSYght samples), and the weighted total count of rare and low-frequency variants for each sample as covariates. We repeated the analysis, including the first four sequencing metadata PCs as covariates. We controlled for multiple testing with a Bonferroni correction for 200 tested states with significance p value threshold calculated as $0.05/200 = 0.00025$; we used a more stringent p value threshold of $0.05/(200 \times 7 \text{ comparisons}) = 3.6 \times 10^{-5}$ to account for multiple analyses.

Test for InPSYght control-TOPMed control enrichment of rare and low-frequency variants in various genomic repeat categories

To investigate the potential effects of the sequencing technical differences between the InPSYght and TOPMed studies on the number of non-reference alleles detected in genomic repeat regions, we annotated each analyzed variant for its presence in a repeat region. We defined repeat regions using (1) repeat regions identified by RepeatMasker 3.0.1 obtained from the UCSC Genome Browser[65] and (2) simple repeats defined by Tandem Repeats Finder.[66] The repeat regions were tested as a class and were further divided into 21 repeat categories, for a total of 22 categories. We tested for differential enrichment of SNVs in each of the repeat categories, without inclusion of the sequencing metadata PCs as described for the ChromHMM and ConsHMM state tests. We controlled for multiple testing with a Bonferroni correction for 22 tested repeat categories with the significance p value threshold calculated as $0.05/22 = 0.0023$; we used a more stringent p value threshold of $0.05/(22 \times 7 \text{ comparisons}) = 3.3 \times 10^{-4}$ to account for multiple analyses.

Genome-wide rare and low-frequency and rare variant sliding window burden tests for case-control and InPSYght control-TOPMed control comparisons

To identify local enrichments of disease-associated rare and low-frequency alleles, we performed sliding window burden tests using WGScan[64] in the same six unrelated-samples case-control sets and one unrelated control-control sample set as in the chromatin and conservation states analysis. We also used the same variants as in the chromatin and conservation states analysis. Following the default parameters of WGScan, we tested variants in window sizes of 5, 10, 15, 20, 25, and 50 kb (including coding and non-coding regions), and upweighting rarer variants with beta function *beta* (MAF, 1, 25) weights. We included the same set of covariates as in the chromatin and conservation states analysis (with and without the first four sequencing metadata PCs). We used WGScan's permutational approach with default parameters (including 5,000 permutation replicates) to estimate the effective number of tests (n) for each comparison group.[64] We controlled for multiple testing with a Bonferroni-type correction, with significance thresholds calculated as $0.05/n$ (2.15×10^{-8} to 2.19×10^{-8}); we used a more stringent p value threshold of $0.05/\sum_1^i n_i = 3.1 \times 10^{-9}$ to account for multiple analyses sets (i), where $\sum_1^i n = 16{,}207{,}362$ is the total number of effective tests across all comparisons.

Secondary analysis for the most strongly associated window across all case-control test combinations

We conducted secondary analyses for the most strongly associated sliding window across all six case-control test combinations: the chr11:64,859,972–64,869,939 association observed in InPSYght BD versus InPSYght controls. We removed variants in the repeat regions (defined above) and performed a WGScan-based burden test of InPSYght BD versus InPSYght controls on this window using the approach described above. We also tested the InPSYght BD cases versus InPSYght controls single-variant association in the non-repeat region of chr11:64,859,972–64,869,939 using a two-sided Fisher's exact test implemented in PLINK 1.9[43] (as many variants had an MAC lower than the SAIGE threshold [MAC <20]). We then performed two additional WGScan-based burden tests for this chr11:64,859,972–64,869,939 region: variants with nominally significant Fisher's exact p values ($p < 0.05$) only and variants in the window that were not nominally significant.

Results

Genome-wide single-variant case-control association analysis

The InPSYght study sample consists of 7,544 African American individuals (estimated African ancestry >25%): 1,598 with BD, 3,295 with SZ, and 2,651 without known BD or SZ or unipolar depression (Table S1). Of all the participants, 42% were female, and participants had an average age of 42.5 ± 12.7 years (Table S3). We generated WGS data for InPSYght samples at an average depth of 26.8 ± 5.5. We estimated the ancestral sources of African ancestry in the samples using the HGDP reference panel and found that almost all InPSYght individuals were genetically most similar to the West African populations represented by Yoruba and Mandenka samples (Figure S2).

To increase power to detect BD- and SZ-variant associations, we included as controls 14,812 African American individuals from the TOPMed study (99.9% of which had >25% African ancestry; average sequencing depth 36.8 ± 4.7). The TOPMed samples came from studies focused on diseases of the heart, lung, and blood and sleep disorders, and inclusion was not predicated on information about mental health (Table S2). To minimize differences between InPSYght and TOPMed samples used in our analyses, we jointly called the samples, and we selected TOPMed samples to have a genetic PC composition similar to that of the InPSYght samples (subjects and methods; Figure S1). The selected TOPMed samples were 62% female. In the jointly called InPSYght and TOPMed external control dataset, we identified 226,434,324 variants (210,210,658 SNVs and 16,223,666 short indels) on the autosomes and chromosome X, 220,310,579 variants of which have MAF <0.05 (204,467,345 SNVs and 15,843,234 short indels).

To identify SNV and short indels associated with BD and SZ, we performed GWAS single-variant tests of association (MAC >20 in the tested group). We adjusted for the first 10

Table 1. λ_{GC} results for each analysis type

		Single-variant GWAS	Gene exon burden	Chromatin/conservation burden		Sliding window burden	
Sample group 1	Sample group 2	Base	Base	Base	+ metadata PCs	Base	+ metadata PCs
InPSYght controls	TOPMed controls	1.02 (S)	[a]	1.51	1.02	1.00	1.01 (S)
InPSYght BD + SZ	InPSYght + TOPMed controls	1.01	0.92	1.54	1.24	1.02	1.01
	InPSYght controls	1.00	[a]	0.98	0.98	0.98	0.98
InPSYght BD	InPSYght + TOPMed controls	1.02 (S)	[a]	1.70 (S)	1.21	1.00	0.99
	InPSYght controls	1.00	[a]	1.13	1.07	0.98	0.98
InPSYght SZ	InPSYght + TOPMed controls	1.00	[a]	1.28	1.10	1.02	1.01
	InPSYght controls	1.00	[a]	0.92	0.93	0.99	0.98

(S) represents at least one case/control genome-wide or category-wide significant association test for one comparison or seven comparisons. BD, bipolar disorder; SZ, schizophrenia.
[a]Comparison was not tested in this study. Base refers to the association test with base covariates (see subjects and methods) without the inclusion of metadata PCs.

genetic PCs, sex, and sequencing batches (only in InPSYght sample analysis) and accounted for relatedness using a mixed model. First, to determine whether differences in genetic ancestry or sequencing between InPSYght and TOPMed samples might cause artifactual associations in the BD and SZ association analysis, we performed a GWAS of InPSYght controls versus TOPMed controls. There was no evidence of inflation of genomic control ($\lambda_{GC} = 1.02$). There was one common genome-wide significant variant, an indel on chromosome 13 (rs11350613) (OR = 0.79 [95% confidence interval {CI} 0.74–0.85], $p = 1.2 \times 10^{-10}$, MAF of 0.63 versus 0.68 in InPSYght controls versus TOPMed controls, respectively) (Figure S3). However, variant rs11350613 is not in LD with other variants in our data, and it just barely passed the SVM-based QC filter in our study (−0.497, threshold for retention SVM >−0.5) and failed QC in the subsequent TOPMed 10 data freeze (https://bravo.sph.umich.edu/variant.html?chrom=13&pos=79615934&ref=C&alt=CT). Second, we estimated the power to detect case-control associations for each case group versus the InPSYght + TOPMed control group. We used $p < 5 \times 10^{-9}$ for genome-wide significance and $p < 5 \times 10^{-9}/7 = 7.1 \times 10^{-10}$ for conservative multiple groups testing genome-wide significance to account for the seven groups of samples being tested. For tests of BD, SZ, and SZ + BD association with InPSYght + TOPMed controls, we have approximately 80% power for $p < 5 \times 10^{-9}$ and $p < 7.1 \times 10^{-10}$ to detect ORs of 3.2, 2.4, and 2.1 and 3.4, 2.5, and 2.3, respectively, for an MAF of 0.01 and for ORs of 1.76, 1.53, and 1.43 and 1.78, 1.55, and 1.44, respectively, for an MAF of 0.05. We performed single-variant association analyses of BD, SZ, or SZ + BD versus InPSYght + TOPMed controls or versus InPSYght controls (Manhattan and quantile-quantile [Q-Q] plots; Figures S3–S10; Table S6). Estimation of population stratification and deviation of test statistics observed from that expected (λ_{GC}) ranged from 1.00 to 1.02 for the various case-control combinations tested, consistent with minimal stratification bias or p value inflation (Table 1). We observed one genome-wide significant before multiple group testing correction (between $p < 5 \times 10^{-9}$ and $p < 5 \times 10^{-9}/7$ comparisons = 7.1×10^{-10}), locus on chromosome 18 (2 SNVs and 1 indel) in the BD versus InPSYght + TOPMed control analysis (Figure S6): lead SNV chr18:49738979:G:T, OR (95% CI) = 30.7 (1.35×10^{-9}), $p = 1.3 \times 10^{-9}$, MAF of 0.0069 (BD) versus 0.0011 (InPSYght + TOPMed controls). The three variants are within 600 bp of one another and are in strong LD ($r^2 > 0.9$) (Figure 2). The locus zoom plots display the 1000G AFR LD, and there appears to be a variant in $r^2 > 0.80$ with our associated variants; however, in our data, this variant has $r^2 = 0.65$ with the most strongly associated variants and $p = 0.0014$. We observed less significant association results in the smaller BD versus InPSYght control analysis (chr18:49738979:G:T, OR [95% CI] = 8.51 [3.85–18.8], $p = 1.22 \times 10^{-7}$, MAF of 0.0069 [BD] versus 0.00056 (InPSYght controls) (Table S7). These variants had no obvious quality control issues. The nearest genes in the region are *ACAA2*, *LIPG*, and *MYO5B*.

We found that in chromatin and conservation state tests (see below), sequencing metric-based PCs may help control for sample sequencing differences. We repeated our analysis of this region, including four sequencing metric metadata PCs. We found slightly attenuated non-genome-wide significant results (chr18:49738979:G:T, OR [95% CI] = 15.0 [5.78–39.1], p value 2.7×10^{-8}).

Gene-based and gene set tests

The SCHEMA Consortium exome-sequencing meta-analysis of 24,248 SZ cases and 97,322 controls of predominantly European ancestry individuals tested functional variant annotation groupings for association with SZ; they found that extremely rare PTVs had the strongest SZ associations.[15] The SCHEMA analysis contained InPSYght SZ and control individuals, but did not separately report the results for InPSYght African American individuals. To specifically assess gene-based PTV associations in African American individuals, we used 4,489 InPSYght

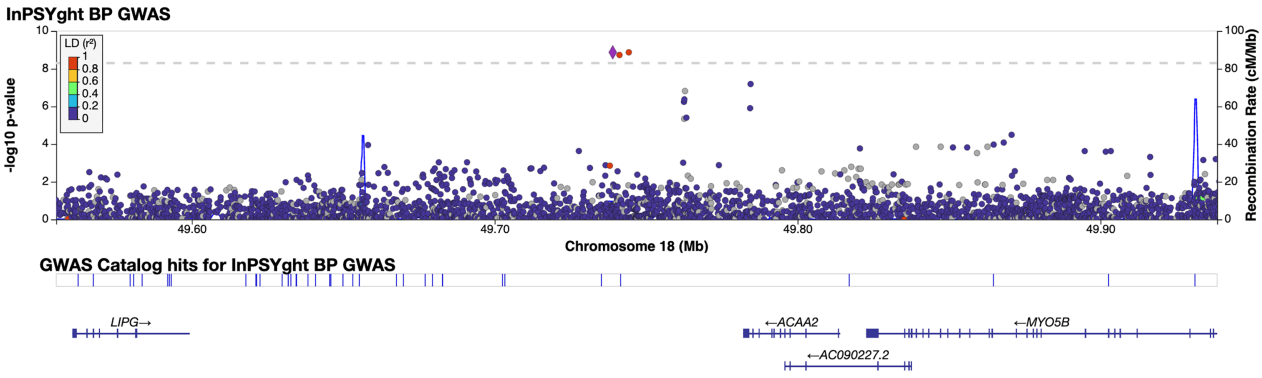

Figure 2. Regional view of chromosome 18 locus showing evidence of association in the InPSYght BD versus InPSYght +TOPMed controls
Horizontal line shows a genome-wide significance threshold of 5×10.

SZ + BD cases and 10,883 InPSYght + TOPMed controls (all unrelated individuals) to test for PTV burden in 1,045 genes with singleton PTV count >10 (see subjects and methods). We included the BD cases to increase the number of alleles per gene given similarities in the underlying genetic architecture.[54] We did not detect evidence of inflation of association statistics ($\lambda_{GC} = 0.92$) and found no SZ + BD-associated genes (Table 1; Figure S10). In a sensitivity analysis expanding the singleton criteria to PTV variants up to 1% MAF, we again found no significant SZ + BD genes (Table S8). Of SCHEMA's 10 most strongly associated SZ genes, 4 genes had no singleton PTVs in the InPSYght + TOPMed sample and 6 had singleton PTVs <10; all had $p > 0.05$ (Table S9). Considering SCHEMA's top 10 genes as a single gene set, we observed directionally consistent, although non-significant, enrichments of PTVs in SZ (OR = 1.65, $p = 0.42$) and SZ + BD cases (OR = 2.07, $p = 0.15$) compared to controls.

We tested three previously identified SZ-associated gene sets for PTV gene set enrichment in SZ or SZ + BD versus InPSYght + TOPMed controls. For the most strongly enriched SCHEMA[15] gene set—3,063 constrained (pLI >0.90) genes[15,59]—we found significant association for both SZ (OR = 1.11, $p = 8.2 \times 10^{-3}$) and SZ + BD (OR = 1.13, $p = 2.9 \times 10^{-3}$) (samples contained within SCHEMA). For two SZ-associated gene sets identified in multiple papers—1,423 post-synaptic density genes[56,57] and 784 FMR1 protein-associated genes[58,59]—we found directionally consistent ORs but no significant associations (Table 2).

Case-control enrichment of rare and low-frequency SNVs in chromatin states and conservation states

We next investigated whether chromatin- or conservation state-based sets of rare and low-frequency SNVs (MAF <0.05) are differentially enriched between the InPSYght and TOPMed controls and between SZ and/or BD cases and InPSYght and/or TOPMed controls (Figure 1, unrelated individuals). Our goal with this analysis was to test a diverse set of systematically defined genomic regions that could potentially capture both technical artifacts and biological associations. For this, we annotated the SNVs using a set of 100-ChromHMM universal chromatin states[60] and a set of 100-ConsHMM conservation states,[61,62] for a total of 200 states (see subjects and methods). For each state, we performed a logistic regression to test whether two groups have a differential variant burden, with upweighting of variants with lower MAF (see subjects and methods).

We tested for state differences in variant burden in InPSYght controls versus TOPMed controls and found that the p value distribution was substantially inflated compared to the expected distribution ($\lambda_{GC} = 1.51$; Figures 3A and 3B; Table 1). Likewise, in case-control analyses that included TOPMed controls, we found substantial p value inflation ($\lambda_{GC} = 1.28$–1.70; Figures 3C, 3D, and S11; Table 1). One state (ConsHMM state 75) had lower variant burden in BD cases compared to InPSYght + TOPMed controls (OR = 0.71, $p = 9.9 \times 10^{-5}$, significant at a per-case-control group level).

To test whether these findings could be due to differences in technical sequencing factors between InPSYght and TOPMed, we constructed a set of sequencing metadata PCs from sequencing quality control metrics (see subjects and methods) and included them as covariates. These sequencing metadata PCs summarize various quality control metrics, including various sequencing depth-related metrics that almost exclusively drove the PCs (Table S5). After inclusion of sequencing metadata PCs, the TOPMed control versus InPSYght controls state-based burden test had a non-inflated $\lambda_{GC} = 1.02$, and the TOPMed control-containing case-control analyses had lower λ_{GC}s (1.10–1.24), with no visible inflation at more significant p values (Figures 3B, 3D, and S11; Table 1). Across all the case-control comparisons, no state reached a Bonferroni-based significance threshold of 0.05/200 chromatin states = 2.5×10^{-4} (the number of total states tested between the two models) after the inclusion of metadata PCs (Figure S12). Thus, we did not find evidence for enrichment in BD and/or SZ cases of rare and low-frequency variants in particular chromatin or conservation states.

Table 2. InPSYght singleton PTV burden test results for previously published SZ gene sets

Gene set	No. of genes	InPSYght study of BD and SZ	InPSYght study of SZ	Genovese et al.[57] (SZ)	Singh et al. (SCHEMA)[15,a] (SZ)
		Case $n = 4{,}489$; control $n = 10{,}883$	Case $n = 3{,}006$; control $n = 10{,}883$	Case $n = 4{,}877$; control $n = 6{,}203$	Case $n = 24{,}248$; control $n = 97{,}322$
		African American ancestry	African American ancestry	European ancestry	Multi-ethnic ancestry
		OR (p value)	OR (p value)	OR (p value)	OR (p value)
Constrained (pLI > 0.90)	3,063	1.13 (0.0030)[b]	1.13 (0.0082)[b]	1.17 (1.7×10^{-8})	1.26 (7.6×10^{-35})
PSD	1,423	1.09 (0.052)	1.11 (0.039)	[c]	1.20 (1.0×10^{-6})
FMRP	784	1.10 (0.058)	1.10 (0.099)	1.23 (8.2×10^{-9})	1.25 (2.2×10^{-17})

[a]Overlap in study sample with InPSYght.
[b]Significant test, $p < 0.0085$
[c]Gene set was not tested in the listed study

Control-control enrichment of rare and low-frequency variants for repeat classes

We next sought to better understand why the inflated λ_{GC} values for chromatin and conservation states in the control-control enrichment analysis had non-inflated λ_{GC} when including sequencing metadata PCs. Given that repeat regions often present technical challenges in sequencing, we hypothesized that repeat regions as a whole or of particular classes might show specific differences in enrichment by control study. This hypothesis would also be consistent with strong enrichments shown for various categories of genomic repeat elements in specific ChromHMM[60] and ConsHMM[61] states. To test this hypothesis, we annotated rare and low-frequency SNVs with a total of 21 different categories of repeat regions defined by RepeatMasker and Tandem Repeats Finder.[66] Using the same test as for state-based analysis, we asked whether the TOPMed and InPSYght controls were differentially enriched for SNV over all repeats and in specific repeat categories. We did not see significant enrichment of SNVs in the overall repeat category (OR = 2.13, $p = 4.1 \times 10^{-2}$, overlapping 53% of variants on average). However, we found significant enrichment of rare and low-frequency variants in TOPMed controls compared to InPSYght controls in the RepeatMasker-defined short interspersed nuclear elements (SINE) (OR = 2.04, $p = 3.0 \times 10^{-9}$, overlapping 16% of variants on average) and simple repeat regions (OR = 1.31, $p = 3.2 \times 10^{-6}$, overlapping 1% of variants on average) (Figures 4A and 4B). After including sequencing metadata PCs as additional covariates, only simple repeat regions remain significant (at a single-analysis set level), with a greatly reduced significance level (OR = 1.35, $p = 2.0 \times 10^{-3}$) (Figures 4C and 4D). Overall, these results suggest that TOPMed and InPSYght cohorts have different distributions of variants in specific repeat categories and could be associated with artifactual associations in burden-type analyses. Such differences can be partially controlled for with the inclusion of sequencing metadata PCs.

Genome-wide rare and low-frequency variant sliding window burden tests for BD and SZ samples versus controls

To identify contiguous genomic regions that might harbor an excess of rare and low-frequency SNVs that predispose to or protect from BD and SZ, we conducted genome-wide 5- to 50-kb sliding window analyses within the TOPMed and InPSYght control groups and 6 case-control groups (Figure 1) using the WGScan framework.[64] Within each genomic window we performed allele frequency weighted burden test, adjusting for covariates used in the chromatin and conservation state analysis (with and without the metadata PCs).

To identify windows that might be affected by differences in sequencing between the TOPMed and InPSYght study, we first compared InPSYght controls and TOPMed controls (Figure 5). The $\lambda_{GC} = 1.01$ was consistent, with no inflation of the test statistics (Table 1). However, we identified a 50-kb region on chromosome 5 (chr5:58,500,010–58,550,007) with 12 windows with genome-wide significant associations, with the strongest association being chr5:58,510,332–58,515,331 ($p = 1.29 \times 10^{-9}$, significant when accounting for multiple analysis sets; Figure 5B). This region showed an elevated burden of rare and low-frequency SNVs for InPSYght controls (mean weighted burden = 156) compared to TOPMed controls (mean weighted burden = 145). To test the robustness of this association, we repeated the analysis without including the sequencing metadata PCs as covariates and found that none of the windows in this region remained significant (minimum $p = 2.16 \times 10^{-3}$) (Figures 5A, 5C, and 5D), leaving open the question of whether this was a chance association or if the metadata PCs induced a false positive association in this region.

In the six case-control analyses we observed no inflation of the test statistics (λ_{GC} = (0.98–1.01)) with the inclusion of metadata PCs (Table 1). We did not identify any window where the burden of rare and low-frequency SNVs was significantly associated with BD and/or SZ. The significant chromosome 5 region from the control-control analysis did not show association signal in any of

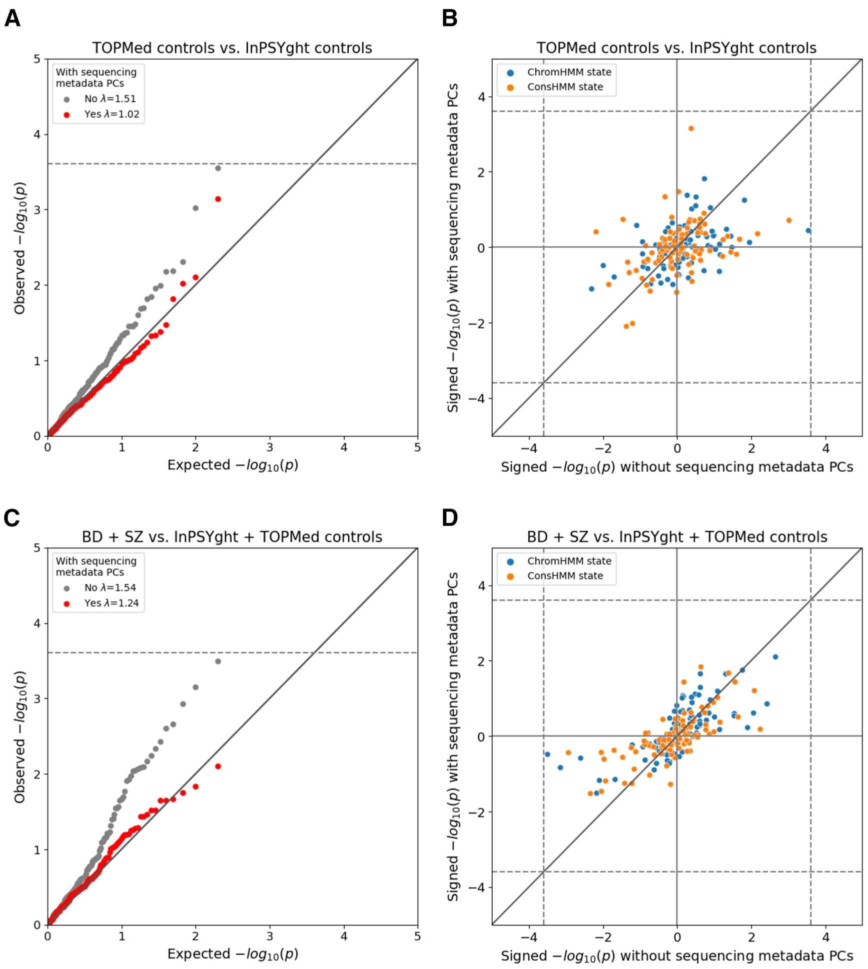

Figure 3. Chromatin and conservation state burden test results

Results for InPSYght controls versus TOPMed controls (A and B) and InPSYght cases versus InPSYght and TOPMed controls (C and D), with each point representing a ChromHMM or a ConsHMM state. Dashed lines show Bonferroni-based p value thresholds ($p = 0.05/200$).
(A and C) Diagonal lines show the unit slope Q-Q plots and genomic inflation factors (λ) before and after inclusion of sequencing metadata PCs covariates.
(B and D) Signed (by direction of enrichment coefficient) $-\log_{10}$ p values before and after inclusion of sequencing metadata PCs covariates. Sign direction: case enrichment values are positive, and control enrichment values are negative.

the case-control analyses ($p \geq 0.01$ for every case-control analysis).

Interestingly, when we compared the sliding window results to BD and SZ GWAS results, we noticed that the most significant window across all case-control tests was located in a 6-Mb region of chromosome 11 containing multiple independent Psychiatric Genomics Consortium common variant BD associations (Table S10).[4] This 10-kb window overlaps the *EHD1* gene (chr11:64,859,972–64,869,939; GRCh38 ($p = 4.06 \times 10^{-8}$ for InPSYght BD versus InPSYght controls and has a higher burden in controls than in cases). This window is the tenth most significant window for the InPSYght BD versus combined InPSYght and TOPMed controls analysis ($p = 1.37 \times 10^{-6}$).

We assessed whether removing repetitive regions might strengthen the association signal because we could more accurately genotype non-repeat variants. In the non-repetitive region analysis of InPSYght BD versus InPSYght controls including sequencing metadata PCs, this window reached genome-wide significance ($p = 1.12 \times 10^{-9}$) and was the most strongly associated window across all case-control comparisons; we found similar results when the analysis was run without metadata PCs ($p = 1.09 \times 10^{-9}$). We identified a specific subset of control-enriched variants belonging to distinct haplotypes (Figure S13). Across cell and tissue types, this 10-kb window has an average of 44.5% of base pairs in the TxReg chromatin state (defined by a high presence of transcription, enhancer, and promoter chromatin marks) from a chromatin state model providing per-cell and tissue annotations[67]; only 0.02% of the 10-kb windows in the genome had a higher percentage of base pairs annotated by this state, suggesting high regulatory potential (Figure S14; see supplemental text for further analysis). These analyses point to a potential convergence of BD-related associations in this region that awaits replication in larger samples.

Discussion

We investigated the genomic basis of SZ and BD across the allele frequency spectrum for coding and non-coding variants in a cohort of African American individuals. We identified three low-frequency BD-associated variants (two SNVs and one indel) on chromosome 18. These variants are 200 kb from a BD association identified in a GWAS study of 1,461 BD cases (bipolar 1 disorder) and 2,008 controls[37]; larger studies have not identified associations in this region.[4,12] In addition to the single-variant results, we identified a significantly associated window on chromosome 11 when performing a secondary BD analysis excluding genomic repeat regions. The associated window is located in a 6-Mb cluster of five common variant GWAS associations for BD.[4] This region has a significantly lower burden of rare and low-frequency and rare variants in BD cases compared to InPSYght controls. Given the number of GWAS and sliding window

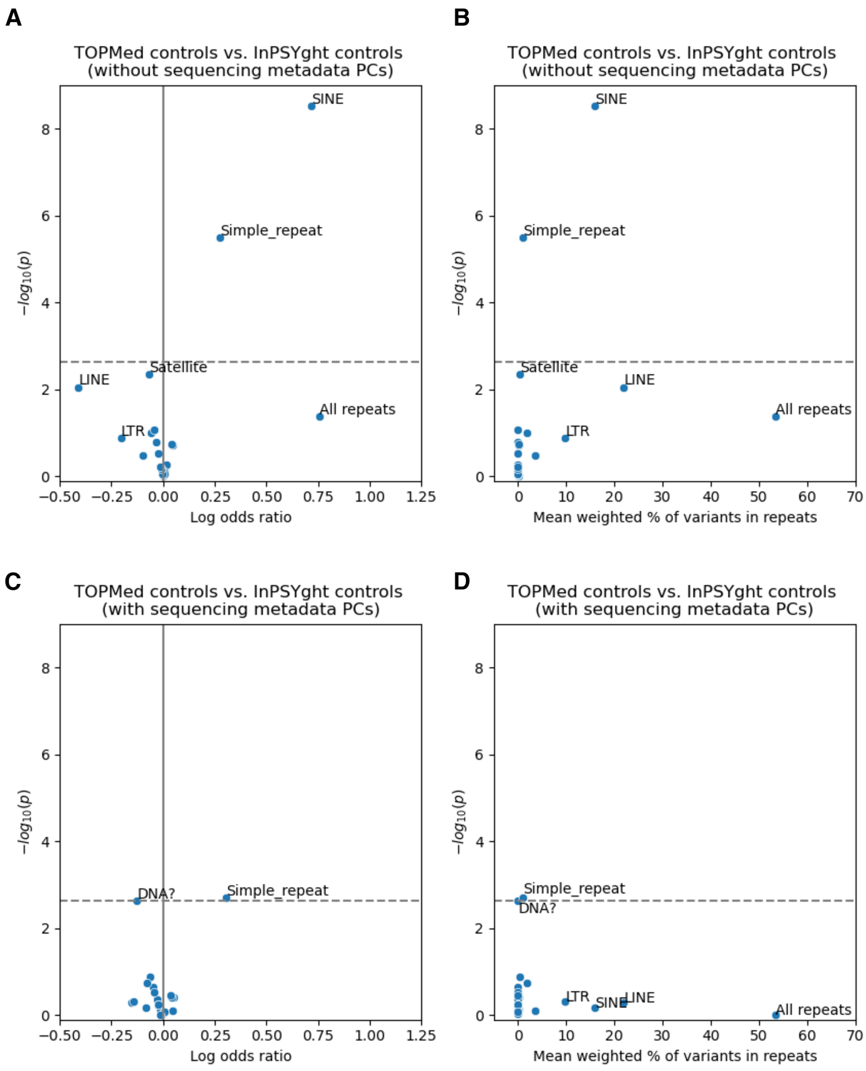

Figure 4. Test of repeat categories for enrichment of rare and low-frequency variants in TOPMed controls versus InPSYght controls

Test of repeat categories without (A and B) or with (C and D) sequencing metadata PCs covariates.

"DNA?" represents elements with uncertain category classification to the DNA repeat element category. Horizontal dashed lines show Bonferroni-based p value thresholds ($p = 0.05/22$).

(A and C) Volcano plots with log odds ratio on the x axis and $-\log_{10} p$ values on the y axis.

(B and D) Mean weighted percentage of variants overlapping each repeat category on the x axis (mean of (per person weighted allele count used in repeat test divided by total weighted alleles count)) see subjects and methods), and $-\log_{10} p$ values on the y axis.

tests we performed (albeit with overlapping sets of cases and controls), these findings would likely not survive a more stringent correction for the effective number of tests performed in our study. Thus, we consider these chromosome 18 and 11 results as potentially associated loci that would need to be assessed in larger WGS studies, including those with African American individuals.

Although we worked to minimize genotype calling differences by calling InPSYght and TOPMed samples together, one potential limitation of our study is that the TOPMed controls were more deeply sequenced and were sequenced separately from InPSYght cases and controls. Across our primary analyses, the only tests that appeared to be sensitive to potential differences in sequencing were the ConsHMM conservation states and of universal ChromHMM chromatin states tests (based on an inflated λ_{GC}). These tests combine large numbers of rare and low-frequency variants from regions that may be more or less difficult to sequence or prone to sequencing artifacts. These states are known to have different levels of repetitive regions,[60,61] and we found that particular classes of repeats, SINE and simple repeat regions, were enriched for significant differences between the InPSYght and TOPMed controls. These findings suggest that sensitivity analyses should be performed for tests that aggregate variants across any class of genomic regions. For example, gene set analysis of non-coding variants may be susceptible to false positives given that different repeat classes are enriched near biologically distinct sets of genes; SINE are enriched in housekeeping genes.[68]

In our initial analyses of genomic regions, we attempted to control for sequencing differences and variant density, in a manner analogous to that of tests of rare singletons, by inclusion of the total weighted rare and low-frequency allele count as a covariate. This covariate was not effective in controlling the false positive rate in the ConsHMM conservation and ChromHMM chromatin states tests. We found, however, that inclusion of sequencing metadata PCs reduced or even eliminated the λ_{GC} inflation. This suggests that for tests that aggregate rare and low-frequency variants (and potentially tests that aggregate singletons), inclusion of more extensive sequence metrics may be necessary to control the false positive rate.

In contrast to their ability to control for p value inflation in the InPSYght versus TOPMed control chromatin and conservation state tests, we observed that the inclusion of the sequencing metadata PCs appeared to induce a false positive association in the sliding window control-control (TOPMed versus InPSYght) analysis. When we included sequencing metadata PCs as covariates, we identified a genome-wide significant region on chromosome 5 that was 6 orders of magnitude more significant than without sequence data metadata PCs. These findings suggest that

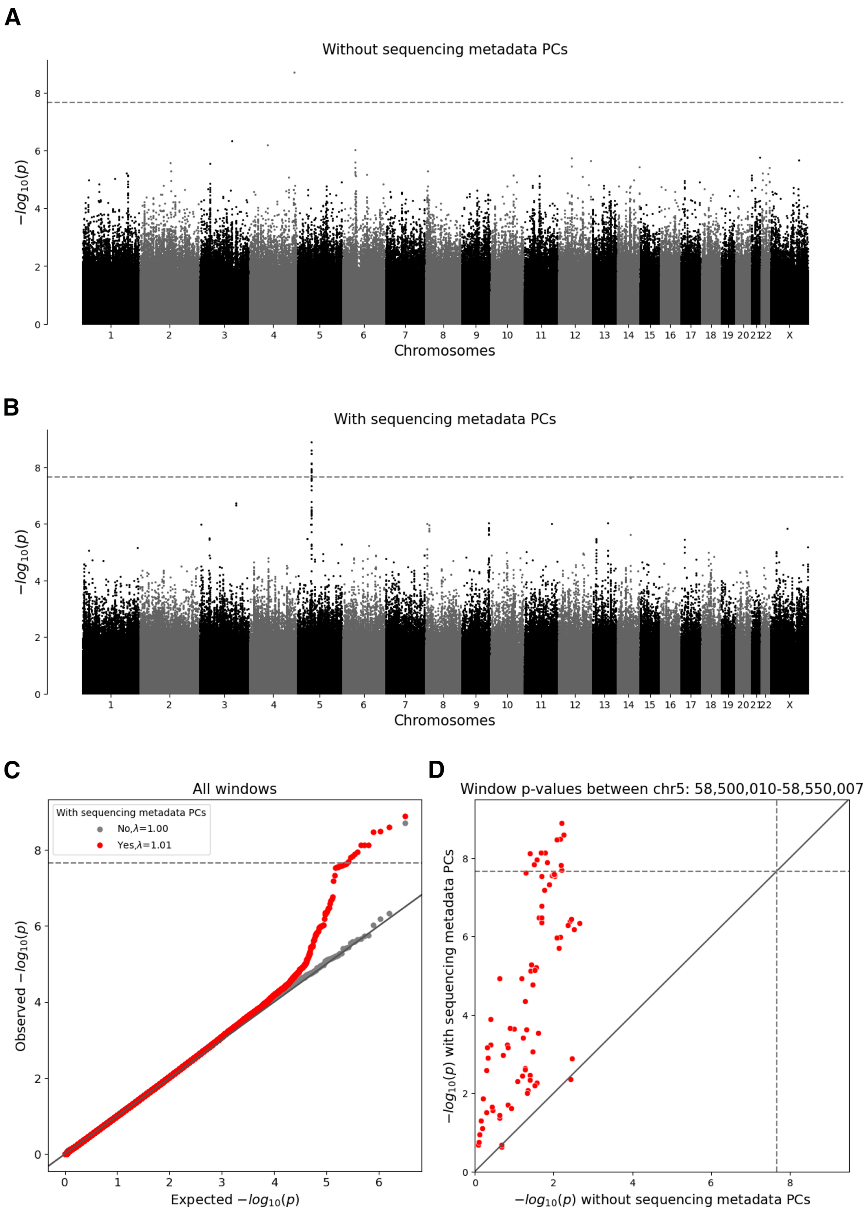

Figure 5. WGScan genome-wide sliding window burden test for rare and low-frequency variants for InPSYght controls versus TOPMed controls

Horizontal lines show genome-wide p value significance thresholds ($p = 2.18 \times 10^{-8}$); diagonal line shows the unit slope.

(A and B) Manhattan plots of sliding window p values without sequencing metadata PCs (A) and with sequencing metadata PCs (B).

(C) Q-Q plot for the window p values, before and after inclusion of sequencing metadata PCs covariates.

(D) Comparison between sliding window p values before and after inclusion of sequencing metadata PCs covariates, showing windows in the chr5 region 58,500,010–58,550,007 (red).

significant associations should be subject to sensitivity analyses with and without adjustment for sequencing-related covariates. The findings also highlight the challenges associated with controlling for batch/sequencing effect differences and variant quality in WGS studies.[69]

There are additional limitations in the present study and analysis. First, we have a very small effective sample size relative to genotype array-based GWASs. We expect that larger WGS case-control samples will be essential to identification of rarer non-coding variants. Second, to have a focused study we only considered SNVs and short indels, and we did not analyze large structural variations including repeating expansions. Third, there was a substantially greater proportion of males in InPSYght cases than in TOPMed control individuals. We corrected for sex in our association testing, which would reduce any (unlikely) differences in allele frequency by sex.

Fourth, the TOPMed controls were not screened for psychiatric disorders, and thus the presence of individuals with BD or SZ in the control group could have decreased our power to detect BD and SZ associations. We expect misclassification in the control group to have a minimal effect[70] because (1) the combined prevalence of BD and SZ is 2%–4% in the general population[71] and (2) individuals with severe mental illness are less likely than individuals without severe mental illness to participate in non-psychiatric disorder-based studies due to exclusion criteria and/or a decreased likelihood of enrollment.[72,73] As improved methods for analyzing rare non-coding variation are developed,[64,70] and the field comes to a clearer consensus on which analytical strategies and annotations are most effective for WGS analysis, more disease-relevant information will be extracted from WGS data.

In summary, WGS allowed us to test variants for association with SZ and/or BD across the allele frequency spectrum in both coding and non-coding regions, in particular, rare non-coding variants that are missed by GWASs or exome sequencing studies. Our study highlights the need to perform sensitivity analysis when conducting WGS analyses, particularly for aggregation tests of rare and low-frequency non-coding variants that use data from multiple studies, and the need for larger WGS studies of African American individuals. Inclusion of African American individuals and other historically under-represented populations in psychiatric studies is crucial to gaining a more comprehensive and equitable

understanding of the contribution of genetic variation to psychiatric disorders and may uncover associations previously unobserved in a more limited set of ancestries.[74] We expect our data and additional African American WGS studies will contribute to the growing understanding of rare non-coding variants and complex psychiatric diseases in individuals with different ancestries.

Data and code availability

- WGS data generated for this study's samples are available in AnVIL: WGSPD Project 1: Whole Genome Sequencing for Schizophrenia and Bipolar Disorder (WGSPD1). The AnVIL Terra Workspaces containing data (AnVIL: https://anvilproject.org/data/studies/phs002041/workspaces) are AnVIL_NIMH_Broad_WGSPD1_McCarroll_Pato_GRU_WGS, ANVIL_NIMH_Broad_WGSPD_1_McCarroll_Braff_DS_WGS, and ANVIL_NIMH_Broad_WGSPD1_McCarroll_COGS_DS_WGS. Phenotype data are available at dbGaP: phs002041.v2.p1. GPC data used in the preparation of this study (genotype data, variables used in analysis, and case-control status), as well as selected results, are available in the NIMH Data Archive (NDA). NDA is a collaborative informatics system created by the National Institutes of Health to provide a national resource to support and accelerate research in mental health. Dataset identifier: https://doi.org/10.15154/1z0s-af20). This manuscript reflects the views of the authors and may not reflect the opinions or views of the NIH or of the Submitters submitting original data to NDA.
- The Freeze 9 TOPMed WGS genotype data used in this study are in dbGaP. See TOPMed projects (above) or Table S2 for the accession numbers.
- The code for ChromHMM and ConsHMM state-based analysis is available at https://github.com/ernstlab/inpsyght_states.

Acknowledgments

This work was supported by the University of Michigan Precision Health Scholar Award (https://precisionhealth.umich.edu/) (S.A.G.T.); R01 HG009976 (M.B.); U01MH105653 (M.B., C.N.P., and S.A.M.); salary support from the Fonds de Recherche du Québec–Santé: a Junior 1 Award, and currently a Junior 2 Award (S.A.G.T.); R01 MH115676 (R.A.O.); and DP1DA044371 and U01HG012079 (J.E.). This work used computational and storage services associated with the Hoffman2 Cluster, which is operated by the University of California, Los Angeles Office of Advanced Research Computing's Research Technology Group. Biosamples and data for the InPSYght study were obtained from the NIMH Repository & Genomics Resource (U24MH068457), a centralized national biorepository for genetic studies of psychiatric disorders (https://www.nimhgenetics.org/). Contributing studies include the GPC, the Consortium of the Genetics of Schizophrenia, the Bipolar Genome Study, the LiTMUS, and the Systematic Treatment Enhancement Program for Bipolar Disorder. We gratefully acknowledge the participants who provided biological samples and data for these studies. We gratefully acknowledge the studies and participants who provided biological samples and data for TOPMed. Molecular data for the TOPMed program were supported by the NHLBI. See the supplemental information for TOPMed study and TOPMed Neurocognitive Working Group members. Core support, including centralized genomic read mapping and genotype calling, variant quality metrics, and filtering, was provided by the TOPMed Informatics Research Center (3R01HL-117626-02S1, contract HHSN268201800002I). Core support, including phenotype harmonization, data management, sample-identity quality control, and program coordination, was provided by the TOPMed Data Coordinating Center (R01HL-120393 and U01HL-120393; contract HHSN268201800001I).

Declaration of interests

A.E.L. is a shareholder of Regeneron Pharmaceuticals. K.C.B. is an employee of Oxford Nanopore Technologies, Ltd. G.A. is a shareholder of Regeneron Pharmaceuticals.

Supplemental information

Supplemental information can be found online at https://doi.org/10.1016/j.xhgg.2025.100499.

Received: January 16, 2025
Accepted: August 22, 2025

References

1. Plana-Ripoll, O., Pedersen, C.B., Agerbo, E., Holtz, Y., Erlangsen, A., Canudas-Romo, V., Andersen, P.K., Charlson, F.J., Christensen, M.K., Erskine, H.E., et al. (2019). A comprehensive analysis of mortality-related health metrics associated with mental disorders: a nationwide, register-based cohort study. Lancet *394*, 1827–1835. https://doi.org/10.1016/S0140-6736(19)32316-5.
2. Song, J., Bergen, S.E., Kuja-Halkola, R., Larsson, H., Landén, M., and Lichtenstein, P. (2015). Bipolar disorder and its relation to major psychiatric disorders: a family-based study in the Swedish population. Bipolar Disord. *17*, 184–193. https://doi.org/10.1111/bdi.12242.
3. Owen, M.J., Sawa, A., and Mortensen, P.B. (2016). Schizophrenia. Lancet *388*, 86–97. https://doi.org/10.1016/S0140-6736(15)01121-6.
4. Mullins, N., Forstner, A.J., O'Connell, K.S., Coombes, B., Coleman, J.R.I., Qiao, Z., Als, T.D., Bigdeli, T.B., Børte, S., Bryois, J., et al. (2021). Genome-wide association study of more than 40,000 bipolar disorder cases provides new insights into the underlying biology. Nat. Genet. *53*, 817–829. https://doi.org/10.1038/s41588-021-00857-4.
5. Gibson, G. (2012). Rare and common variants: twenty arguments. Nat. Rev. Genet. *13*, 135–145. https://doi.org/10.1038/nrg3118.
6. Schizophrenia Psychiatric Genome-Wide Association Study GWAS Consortium (2011). Genome-wide association study identifies five new schizophrenia loci. Nat. Genet. *43*, 969–976. https://doi.org/10.1038/ng.940.
7. Ripke, S., O'Dushlaine, C., Chambert, K., Moran, J.L., Kähler, A.K., Akterin, S., Bergen, S.E., Collins, A.L., Crowley, J.J., Fromer, M., et al. (2013). Genome-wide association analysis identifies 13 new risk loci for schizophrenia. Nat. Genet. *45*, 1150–1159. https://doi.org/10.1038/ng.2742.

8. Schizophrenia Working Group of the Psychiatric Genomics Consortium (2014). Biological insights from 108 schizophrenia-associated genetic loci. Nature *511*, 421–427. https://doi.org/10.1038/nature13595.
9. Pardiñas, A.F., Holmans, P., Pocklington, A.J., Escott-Price, V., Ripke, S., Carrera, N., Legge, S.E., Bishop, S., Cameron, D., Hamshere, M.L., et al. (2018). Common schizophrenia alleles are enriched in mutation-intolerant genes and in regions under strong background selection. Nat. Genet. *50*, 381–389. https://doi.org/10.1038/s41588-018-0059-2.
10. Lam, M., Chen, C.-Y., Li, Z., Martin, A.R., Bryois, J., Ma, X., Gaspar, H., Ikeda, M., Benyamin, B., Brown, B.C., et al. (2019). Comparative genetic architectures of schizophrenia in East Asian and European populations. Nat. Genet. *51*, 1670–1678. https://doi.org/10.1038/s41588-019-0512-x.
11. Sklar, P., Ripke, S., Scott, L.J., Andreassen, O.A., Cichon, S., Craddock, N., Edenberg, H.J., Nurnberger, J.I., Rietschel, M., Blackwood, D., et al. (2011). Large-scale genome-wide association analysis of bipolar disorder identifies a new susceptibility locus near ODZ4. Nat. Genet. *43*, 977–983. https://doi.org/10.1038/ng.943.
12. Stahl, E.A., Breen, G., Forstner, A.J., McQuillin, A., Ripke, S., Trubetskoy, V., Mattheisen, M., Wang, Y., Coleman, J.R.I., Gaspar, H.A., et al. (2019). Genome-wide association study identifies 30 loci associated with bipolar disorder. Nat. Genet. *51*, 793–803. https://doi.org/10.1038/s41588-019-0397-8.
13. Trubetskoy, V., Pardiñas, A.F., Qi, T., Panagiotaropoulou, G., Awasthi, S., Bigdeli, T.B., Bryois, J., Chen, C.-Y., Dennison, C.A., Hall, L.S., et al. (2022). Mapping genomic loci implicates genes and synaptic biology in schizophrenia. Nature *604*, 502–508. https://doi.org/10.1038/s41586-022-04434-5.
14. Zeng, J., Xue, A., Jiang, L., Lloyd-Jones, L.R., Wu, Y., Wang, H., Zheng, Z., Yengo, L., Kemper, K.E., Goddard, M.E., et al. (2021). Widespread signatures of natural selection across human complex traits and functional genomic categories. Nat. Commun. *12*, 1164. https://doi.org/10.1038/s41467-021-21446-3.
15. Singh, T., Poterba, T., Curtis, D., Akil, H., Al Eissa, M., Barchas, J.D., Bass, N., Bigdeli, T.B., Breen, G., Bromet, E.J., et al. (2022). Rare coding variants in ten genes confer substantial risk for schizophrenia. Nature *604*, 509–516. https://doi.org/10.1038/s41586-022-04556-w.
16. Palmer, D.S., Howrigan, D.P., Chapman, S.B., Adolfsson, R., Bass, N., Blackwood, D., Boks, M.P.M., Chen, C.-Y., Churchhouse, C., Corvin, A.P., et al. (2022). Exome sequencing in bipolar disorder identifies AKAP11 as a risk gene shared with schizophrenia. Nat. Genet. *54*, 541–547. https://doi.org/10.1038/s41588-022-01034-x.
17. Homann, O.R., Misura, K., Lamas, E., Sandrock, R.W., Nelson, P., McDonough, S.I., and DeLisi, L.E. (2016). Whole-genome sequencing in multiplex families with psychoses reveals mutations in the SHANK2 and SMARCA1 genes segregating with illness. Mol. Psychiatr. *21*, 1690–1695. https://doi.org/10.1038/mp.2016.24.
18. Khan, F.F., Melton, P.E., McCarthy, N.S., Morar, B., Blangero, J., Moses, E.K., and Jablensky, A. (2018). Whole genome sequencing of 91 multiplex schizophrenia families reveals increased burden of rare, exonic copy number variation in schizophrenia probands and genetic heterogeneity. Schizophr. Res. *197*, 337–345. https://doi.org/10.1016/j.schres.2018.02.034.
19. Sul, J.H., Service, S.K., Huang, A.Y., Ramensky, V., Hwang, S.-G., Teshiba, T.M., Park, Y., Ori, A.P.S., Zhang, Z., Mullins, N., et al. (2020). Contribution of common and rare variants to bipolar disorder susceptibility in extended pedigrees from population isolates. Transl. Psychiatry *10*, 74. https://doi.org/10.1038/s41398-020-0758-1.
20. Alkelai, A., Greenbaum, L., Docherty, A.R., Shabalin, A.A., Povysil, G., Malakar, A., Hughes, D., Delaney, S.L., Peabody, E.P., McNamara, J., et al. (2022). The benefit of diagnostic whole genome sequencing in schizophrenia and other psychotic disorders. Mol. Psychiatr. *27*, 1435–1447. https://doi.org/10.1038/s41380-021-01383-9.
21. Georgi, B., Craig, D., Kember, R.L., Liu, W., Lindquist, I., Nasser, S., Brown, C., Egeland, J.A., Paul, S.M., and Bućan, M. (2014). Genomic View of Bipolar Disorder Revealed by Whole Genome Sequencing in a Genetic Isolate. PLoS Genet. *10*, e1004229. https://doi.org/10.1371/journal.pgen.1004229.
22. Mojarad, B.A., Yin, Y., Manshaei, R., Backstrom, I., Costain, G., Heung, T., Merico, D., Marshall, C.R., Bassett, A.S., and Yuen, R.K.C. (2021). Genome sequencing broadens the range of contributing variants with clinical implications in schizophrenia. Transl. Psychiatry *11*, 84. https://doi.org/10.1038/s41398-021-01211-2.
23. Biernacka, J., Jenkins, G., McDonnell, S., Batzler, A., Sicotte, H., Fogarty, Z., Welkie, B., Baheti, S., Coombes, B., McElroy, S., and Frye, M. (2019). A whole genome sequencing study identifies a rare variant in ANK3 that may contribute to bipolar disorder. Eur. Neuropsychopharmacol. *29*, S901.
24. Halvorsen, M., Huh, R., Oskolkov, N., Wen, J., Netotea, S., Giusti-Rodriguez, P., Karlsson, R., Bryois, J., Nystedt, B., Ameur, A., et al. (2020). Increased burden of ultra-rare structural variants localizing to boundaries of topologically associated domains in schizophrenia. Nat. Commun. *11*, 1842. https://doi.org/10.1038/s41467-020-15707-w.
25. Popejoy, A.B., and Fullerton, S.M. (2016). Genomics is failing on diversity. Nature *538*, 161–164. https://doi.org/10.1038/538161a.
26. Sirugo, G., Williams, S.M., and Tishkoff, S.A. (2019). The Missing Diversity in Human Genetic Studies. Cell *177*, 26–31. https://doi.org/10.1016/j.cell.2019.02.048.
27. Martin, A.R., Kanai, M., Kamatani, Y., Okada, Y., Neale, B.M., and Daly, M.J. (2019). Clinical use of current polygenic risk scores may exacerbate health disparities. Nat. Genet. *51*, 584–591. https://doi.org/10.1038/s41588-019-0379-x.
28. Mahajan, A., Wessel, J., Willems, S.M., Zhao, W., Robertson, N.R., Chu, A.Y., Gan, W., Kitajima, H., Taliun, D., Rayner, N.W., et al. (2018). Refining the accuracy of validated target identification through coding variant fine-mapping in type 2 diabetes. Nat. Genet. *50*, 559–571. https://doi.org/10.1038/s41588-018-0084-1.
29. Spracklen, C.N., Horikoshi, M., Kim, Y.J., Lin, K., Bragg, F., Moon, S., Suzuki, K., Tam, C.H.T., Tabara, Y., Kwak, S.-H., et al. (2020). Identification of type 2 diabetes loci in 433,540 East Asian individuals. Nature *582*, 240–245. https://doi.org/10.1038/s41586-020-2263-3.
30. Taliun, D., Harris, D.N., Kessler, M.D., Carlson, J., Szpiech, Z.A., Torres, R., Taliun, S.A.G., Corvelo, A., Gogarten, S.M., Kang, H.M., et al. (2021). Sequencing of 53,831 diverse genomes from the NHLBI TOPMed Program. Nature *590*, 290–299. https://doi.org/10.1038/s41586-021-03205-y.
31. Sanders, S.J., Neale, B.M., Huang, H., Werling, D.M., An, J.-Y., Dong, S., Whole Genome Sequencing for Psychiatric Disorders WGSPD, Abecasis, G., Arguello, P.A., Blangero, J., et al.

(2017). Whole genome sequencing in psychiatric disorders: the WGSPD consortium. Nat. Neurosci. *20*, 1661–1668. https://doi.org/10.1038/s41593-017-0017-9.

32. Pato, M.T., Sobell, J.L., Medeiros, H., Abbott, C., Sklar, B.M., Buckley, P.F., Bromet, E.J., Escamilla, M.A., Fanous, A.H., Lehrer, D.S., et al. (2013). The genomic psychiatry cohort: Partners in discovery. Am. J. Med. Genet. B Neuropsychiatr. Genet. *162B*, 306–312. https://doi.org/10.1002/ajmg.b.32160.
33. Bigdeli, T.B., Genovese, G., Georgakopoulos, P., Meyers, J.L., Peterson, R.E., Iyegbe, C.O., Medeiros, H., Valderrama, J., Achtyes, E.D., Kotov, R., et al. (2020). Contributions of common genetic variants to risk of schizophrenia among individuals of African and Latino ancestry. Mol. Psychiatr. *25*, 2455–2467. https://doi.org/10.1038/s41380-019-0517-y.
34. Swerdlow, N.R., Gur, R.E., and Braff, D.L. (2015). Consortium on the Genetics of Schizophrenia (COGS) assessment of endophenotypes for schizophrenia: An introduction to this Special Issue of schizophrenia research. Schizophr. Res. *163*, 9–16. https://doi.org/10.1016/j.schres.2014.09.047.
35. Smith, E.N., Bloss, C.S., Badner, J.A., Barrett, T., Belmonte, P.L., Berrettini, W., Byerley, W., Coryell, W., Craig, D., Edenberg, H.J., et al. (2009). Genome-wide association study of bipolar disorder in European American and African American individuals. Mol. Psychiatr. *14*, 755–763. https://doi.org/10.1038/mp.2009.43.
36. Nierenberg, A.A., Friedman, E.S., Bowden, C.L., Sylvia, L.G., Thase, M.E., Ketter, T., Ostacher, M.J., Leon, A.C., Reilly-Harrington, N., Iosifescu, D.V., et al. (2013). Lithium Treatment Moderate-Dose Use Study (LiTMUS) for Bipolar Disorder: A Randomized Comparative Effectiveness Trial of Optimized Personalized Treatment With and Without Lithium. Am. J. Psychiatr.
37. Sklar, P., Smoller, J.W., Fan, J., Ferreira, M.A.R., Perlis, R.H., Chambert, K., Nimgaonkar, V.L., McQueen, M.B., Faraone, S.V., Kirby, A., et al. (2008). Whole-genome association study of bipolar disorder. Mol. Psychiatr. *13*, 558–569. https://doi.org/10.1038/sj.mp.4002151.
38. Jun, G., Wing, M.K., Abecasis, G.R., and Kang, H.M. (2015). An efficient and scalable analysis framework for variant extraction and refinement from population-scale DNA sequence data. Genome Res. *25*, 918–925. https://doi.org/10.1101/gr.176552.114.
39. Center for Statistical Genetics (2020). Statgen: Topmed Variant Calling (GitHub). https://github.com/statgen/topmed_variant_calling.
40. Zhang, F., Flickinger, M., Taliun, S.A.G., InPSYght Psychiatric Genetics Consortium, Abecasis, G.R., Scott, L.J., McCaroll, S.A., Pato, C.N., Boehnke, M., and Kang, H.M. (2020). Ancestry-agnostic estimation of DNA sample contamination from sequence reads. Genome Res. *30*, 185–194. https://doi.org/10.1101/gr.246934.118.
41. Price, A.L., Weale, M.E., Patterson, N., Myers, S.R., Need, A.C., Shianna, K.V., Ge, D., Rotter, J.I., Torres, E., Taylor, K.D., et al. (2008). Long-Range LD Can Confound Genome Scans in Admixed Populations. Am. J. Hum. Genet. *83*, 132–139. https://doi.org/10.1016/j.ajhg.2008.06.005.
42. Purcell, S., Neale, B., Todd-Brown, K., Thomas, L., Ferreira, M.A.R., Bender, D., Maller, J., Sklar, P., De Bakker, P.I.W., Daly, M.J., and Sham, P.C. (2007). PLINK: A Tool Set for Whole-Genome Association and Population-Based Linkage Analyses. Am. J. Hum. Genet. *81*, 559–575. https://doi.org/10.1086/519795.
43. Rosenberg, N.A., Pritchard, J.K., Weber, J.L., Cann, H.M., Kidd, K.K., Zhivotovsky, L.A., and Feldman, M.W. (2002). Genetic Structure of Human Populations. Science *298*, 2381–2385.
44. Manichaikul, A., Mychaleckyj, J.C., Rich, S.S., Daly, K., Sale, M., and Chen, W.-M. (2010). Robust relationship inference in genome-wide association studies. Bioinformatics *26*, 2867–2873. https://doi.org/10.1093/bioinformatics/btq559.
45. Alexander, D.H., Novembre, J., and Lange, K. (2009). Fast model-based estimation of ancestry in unrelated individuals. Genome Res. *19*, 1655–1664. https://doi.org/10.1101/gr.094052.109.
46. 1000 Genomes Project Consortium, Auton, A., Brooks, L.D., Durbin, R.M., Garrison, E.P., Kang, H.M., Korbel, J.O., Marchini, J.L., McCarthy, S., McVean, G.A., and Abecasis, G.R. (2015). A global reference for human genetic variation. Nature *526*, 68–74. https://doi.org/10.1038/nature15393.
47. Maples, B.K., Gravel, S., Kenny, E.E., and Bustamante, C.D. (2013). RFMix: A Discriminative Modeling Approach for Rapid and Robust Local-Ancestry Inference. Am. J. Hum. Genet. *93*, 278–288. https://doi.org/10.1016/j.ajhg.2013.06.020.
48. Fan, S., Spence, J.P., Feng, Y., Hansen, M.E.B., Terhorst, J., Beltrame, M.H., Ranciaro, A., Hirbo, J., Beggs, W., Thomas, N., et al. (2023). Whole-genome sequencing reveals a complex African population demographic history and signatures of local adaptation. Cell *186*, 923–939.e14. https://doi.org/10.1016/j.cell.2023.01.042.
49. Cann, H.M., de Toma, C., Cazes, L., Legrand, M.-F., Morel, V., Piouffre, L., Bodmer, J., Bodmer, W.F., Bonne-Tamir, B., Cambon-Thomsen, A., et al. (2002). A Human Genome Diversity Cell Line Panel. Science *296*, 261–262. https://doi.org/10.1126/science.296.5566.261b.
50. Li, J.Z., Absher, D.M., Tang, H., Southwick, A.M., Casto, A.M., Ramachandran, S., Cann, H.M., Barsh, G.S., Feldman, M., Cavalli-Sforza, L.L., and Myers, R.M. (2008). Worldwide Human Relationships Inferred from Genome-Wide Patterns of Variation. Science *319*, 1100–1104. https://doi.org/10.1126/science.1153717.
51. Wang, C., Zhan, X., Bragg-Gresham, J., Kang, H.M., Stambolian, D., Chew, E.Y., Branham, K.E., Heckenlively, J., FUSION Study, Fulton, R., et al. (2014). Ancestry estimation and control of population stratification for sequence-based association studies. Nat. Genet. *46*, 409–415. https://doi.org/10.1038/ng.2924.
52. Purcell, S., Cherny, S.S., and Sham, P.C. (2003). Genetic Power Calculator: design of linkage and association genetic mapping studies of complex traits. Bioinformatics *19*, 149–150. https://doi.org/10.1093/bioinformatics/19.1.149.
53. Zhou, W., Nielsen, J.B., Fritsche, L.G., Dey, R., Gabrielsen, M.E., Wolford, B.N., LeFaive, J., VandeHaar, P., Gagliano, S.A., Gifford, A., et al. (2018). Efficiently controlling for case-control imbalance and sample relatedness in large-scale genetic association studies. Nat. Genet. *50*, 1335–1341. https://doi.org/10.1038/s41588-018-0184-y.
54. Cross-Disorder Group of the Psychiatric Genomics Consortium (2013). Identification of risk loci with shared effects on five major psychiatric disorders: a genome-wide analysis. Lancet *381*, 1371–1379. https://doi.org/10.1016/S0140-6736(12)62129-1.

55. Zhan, X., Hu, Y., Li, B., Abecasis, G.R., and Liu, D.J. (2016). RVTESTS: an efficient and comprehensive tool for rare variant association analysis using sequence data. Bioinformatics *32*, 1423–1426. https://doi.org/10.1093/bioinformatics/btw079.
56. Purcell, S.M., Moran, J.L., Fromer, M., Ruderfer, D., Solovieff, N., Roussos, P., O'Dushlaine, C., Chambert, K., Bergen, S.E., Kähler, A., et al. (2014). A polygenic burden of rare disruptive mutations in schizophrenia. Nature *506*, 185–190. https://doi.org/10.1038/nature12975.
57. Genovese, G., Fromer, M., Stahl, E.A., Ruderfer, D.M., Chambert, K., Landén, M., Moran, J.L., Purcell, S.M., Sklar, P., Sullivan, P.F., et al. (2016). Increased burden of ultra-rare protein-altering variants among 4,877 individuals with schizophrenia. Nat. Neurosci. *19*, 1433–1441. https://doi.org/10.1038/nn.4402.
58. Fromer, M., Pocklington, A.J., Kavanagh, D.H., Williams, H.J., Dwyer, S., Gormley, P., Georgieva, L., Rees, E., Palta, P., Ruderfer, D.M., et al. (2014). De novo mutations in schizophrenia implicate synaptic networks. Nature *506*, 179–184. https://doi.org/10.1038/nature12929.
59. Singh, T., Walters, J.T.R., Johnstone, M., Curtis, D., Suvisaari, J., Torniainen, M., Rees, E., Iyegbe, C., Blackwood, D., McIntosh, A.M., et al. (2017). The contribution of rare variants to risk of schizophrenia in individuals with and without intellectual disability. Nat. Genet. *49*, 1167–1173. https://doi.org/10.1038/ng.3903.
60. Vu, H., and Ernst, J. (2022). Universal annotation of the human genome through integration of over a thousand epigenomic datasets. Genome Biol. *23*, 9. https://doi.org/10.1186/s13059-021-02572-z.
61. Arneson, A., and Ernst, J. (2019). Systematic discovery of conservation states for single-nucleotide annotation of the human genome. Commun. Biol. *2*, 248. https://doi.org/10.1038/s42003-019-0488-1.
62. Arneson, A., Felsheim, B., Chien, J., and Ernst, J. (2020). ConsHMM Atlas: conservation state annotations for major genomes and human genetic variation. NAR Genom. Bioinform. *2*, lqaa104. https://doi.org/10.1093/nargab/lqaa104.
63. Amemiya, H.M., Kundaje, A., and Boyle, A.P. (2019). The ENCODE Blacklist: Identification of Problematic Regions of the Genome. Sci. Rep. *9*, 9354. https://doi.org/10.1038/s41598-019-45839-z.
64. He, Z., Xu, B., Buxbaum, J., and Ionita-Laza, I. (2019). A genome-wide scan statistic framework for whole-genome sequence data analysis. Nat. Commun. *10*, 3018. https://doi.org/10.1038/s41467-019-11023-0.
65. Karolchik, D., Baertsch, R., Diekhans, M., Furey, T.S., Hinrichs, A., Lu, Y.T., Roskin, K.M., Schwartz, M., Sugnet, C.W., Thomas, D.J., et al. (2003). The UCSC Genome Browser Database. Nucleic Acids Res. *31*, 51–54. https://doi.org/10.1093/nar/gkg129.
66. Benson, G. (1999). Tandem repeats finder: a program to analyze DNA sequences. Nucleic Acids Res. *27*, 573–580. https://doi.org/10.1093/nar/27.2.573.
67. Ernst, J., and Kellis, M. (2015). Large-scale imputation of epigenomic datasets for systematic annotation of diverse human tissues. Nat. Biotechnol. *33*, 364–376. https://doi.org/10.1038/nbt.3157.
68. Lu, J.Y., Shao, W., Chang, L., Yin, Y., Li, T., Zhang, H., Hong, Y., Percharde, M., Guo, L., Wu, Z., et al. (2020). Genomic Repeats Categorize Genes with Distinct Functions for Orchestrated Regulation. Cell Rep. *30*, 3296–3311.e5. https://doi.org/10.1016/j.celrep.2020.02.048.
69. Tom, J.A., Reeder, J., Forrest, W.F., Graham, R.R., Hunkapiller, J., Behrens, T.W., and Bhangale, T.R. (2017). Identifying and mitigating batch effects in whole genome sequencing data. BMC Bioinf. *18*, 351. https://doi.org/10.1186/s12859-017-1756-z.
70. Werling, D.M., Brand, H., An, J.-Y., Stone, M.R., Zhu, L., Glessner, J.T., Collins, R.L., Dong, S., Layer, R.M., Markenscoff-Papadimitriou, E., et al. (2018). An analytical framework for whole-genome sequence association studies and its implications for autism spectrum disorder. Nat. Genet. *50*, 727–736. https://doi.org/10.1038/s41588-018-0107-y.
71. Robinson, N., and Bergen, S.E. (2021). Environmental Risk Factors for Schizophrenia and Bipolar Disorder and Their Relationship to Genetic Risk: Current Knowledge and Future Directions. Front. Genet. *12*, 686666. https://doi.org/10.3389/fgene.2021.686666.
72. Legge, S.E., Pardiñas, A.F., Woolway, G., Rees, E., Cardno, A.G., Escott-Price, V., Holmans, P., Kirov, G., Owen, M.J., O'Donovan, M.C., and Walters, J.T.R. (2024). Genetic and Phenotypic Features of Schizophrenia in the UK Biobank. JAMA Psychiatry *81*, 681–690. https://doi.org/10.1001/jamapsychiatry.2024.0200.
73. Moskvina, V., Holmans, P., Schmidt, K.M., and Craddock, N. (2005). Design of Case-controls Studies with Unscreened Controls. Ann. Hum. Genet. *69*, 566–576. https://doi.org/10.1111/j.1529-8817.2005.00175.x.
74. Ragsdale, A.P., Weaver, T.D., Atkinson, E.G., Hoal, E.G., Möller, M., Henn, B.M., and Gravel, S. (2023). A weakly structured stem for human origins in Africa. Nature *617*, 755–763. https://doi.org/10.1038/s41586-023-06055-y.

HGGA, Volume 7

Supplemental information

Whole-genome sequence-based association analysis

of African American individuals

with bipolar disorder and schizophrenia

Runjia Li, Sarah A. Gagliano Taliun, Kevin Liao, Matthew Flickinger, Janet L. Sobell, Giulio Genovese, Adam E. Locke, Rebeca Rothwell Chiu, Jonathon LeFaive, Jiongming Wang, Taylor Martins, Sinéad Chapman, Anna Neumann, Robert E. Handsaker, Donna K. Arnett, Kathleen C. Barnes, Eric Boerwinkle, David Braff, Brian E. Cade, Myriam Fornage, Richard A. Gibbs, Karin F. Hoth, Lifang Hou, Charles Kooperberg, Ruth J.F. Loos, Ginger A. Metcalf, Courtney G. Montgomery, Alanna C. Morrison, Zhaohui S. Qin, Susan Redline, Alexander P. Reiner, Stephen S. Rich, Jerome I. Rotter, Kent D. Taylor, Karine A. Viaud-Martinez, National Heart, Lung, and Blood Institute Trans-Omics for Precision Medicine (TOPMed) Consortium, Genomic Psychiatry Cohort Investigators, Tim B. Bigdeli, Stacey Gabriel, Sebastian Zollner, Albert V. Smith, Goncalo Abecasis, Steve A. McCarroll, Michele T. Pato, Carlos N. Pato, Michael Boehnke, James Knowles, Hyun Min Kang, Roel A. Ophoff, Jason Ernst, and Laura J. Scott

Supplemental Information

Table of Contents

TOPMed Banner Authors ... 2
The Genomic Psychiatry Cohort: Partners in Discovery authorships ... 3
Supplemental Text ... 4
Supplemental Tables ... 7
Supplemental Figures ... 17
TOPMed Study Short Acknowledgements with dbGaP accession numbers ... 33
TOPMed Study-specific Acknowledgements ... 35
TOPMed Neurocognitive Working Group ... 40
Supplemental References ... 45

TOPMed Banner Authors

(Full list of authors can be found in Table S11.)

The Genomic Psychiatry Cohort: Partners in Discovery authorships

Michele T. Pato[1], Janet L. Sobell[1], Helena Medeiros[1], Colony Abbott[1], Brooke Skar[1], Peter F. Buckley[2], Evelyn J. Bromet[3], Michael A. Escamilla[4], Ayman H. Fanous[5], Douglas S. Lehrer[6], Fabio Macciardi[7], Dolores Malaspina[8], Steve A. McCarroll[9,10], Stephen R. Marder[11], Jennifer Moran[9], Christopher P. Morley[12], Humberto Nicolini[13], Diana O. Perkins[14], Shaun M. Purcell[15], Mark H. Rapaport[16], Pamela Sklar[17], Jordan W. Smoller[18], James A. Knowles[1], Genomic Psychiatry Cohort Consortium*, and Carlos N. Pato[1]

1 Department of Psychiatry and the Behavioral Sciences, University of Southern California, Los Angeles, CA, USA
2 Department of Psychiatry, Georgia Regents University, Augusta, GA, USA
3 Department of Psychiatry and Behavioral Science, State University of New York, Stony Brook, NY, USA
4 Department of Psychiatry, Texas Tech University Health Sciences Center, El Paso, TX, USA 5 Department of Psychiatry, Veterans Administration Medical Center, Washington, DC, USA
6 Department of Psychiatry, Wright State University, Dayton, OH, USA
7 Department of Psychiatry, University of California, Irvine, CA, USA
8 Department of Psychiatry, New York University, New York, NY, USA
9 Stanley Center for Psychiatric Research, Broad Institute of MIT and Harvard, Cambridge, MA, USA
10 Department of Genetics, Harvard Medical School, Boston, MA, USA
11 Department of Psychiatry, University of California, Los Angeles, CA, USA
12 Departments of Family Medicine, Public Health and Preventive Medicine, and Psychiatry and Behavioral Science, State University of New York, Upstate Medical University, Syracuse, NY, USA 13 Carracci Medical Group, Mexico City, MX
14 Department of Psychiatry, University of North Carolina, Chapel Hill, NC, USA
15 Center for Human Genome Research, Massachusetts General Hospital, Boston, MA, USA
16 Department of Psychiatry and Behavioral Sciences, Emory University, Atlanta, GA, USA
17 Department of Psychiatry, Mt. Sinai School of Medicine, New York, NY, USA
18 Department of Psychiatry, Harvard University, Boston, MA, USA

Supplemental Text

Follow-up analyses for the top window across all case-control test combinations

We conducted follow-up analyses for the top window across all six case-control test combinations, that is chr11:64,859,972-64,869,939 ($p=4.06 \times 10^{-8}$ with repeats and $p=1.12 \times 10^{-9}$ without repeats for InPSYght BD vs. InPSYght controls) (**Figure S13A, B**).

We first analyzed the extent to which the most significant variants could explain the overall association signal. To do this we first performed single-variant two-sided Fisher's exact test (Methods) on each of the 197 variants not overlapping repeats in the region for InPSYght BD cases versus controls. The 12 most significant variants in this region had p-values within range [1.5×10^{-4}, 1.5×10^{-2}] while no other individual variant was marginally significant (p-value <0.05). 11 out of these 12 variants are noncoding, with chr11:64859972G,A being a synonymous coding variant. When we restricted the burden test to only these 12 variants, we observed a p-value of 9.0×10^{-8} whereas performing the same test on all other variants in the window only yields a p-value of 4.8×10^{-3}, indicating a major contribution from the 12 nominally significant variants. These variants constitute two distinct haplotypes, both of which being abundant among samples African American individuals (**Figure S13C**).

The *EHD1* gene which overlaps the significant window was previously shown to play potential roles in neuropsychiatric disorders. Earlier studies established EHD1 as mediators of synaptic exo- and endocytosis[1–3]. A small-sample WES study for BD trios identified a *de novo* frame-shift mutation within the *EHD1* gene[3] and a follow-up knock-in mice study suggested that this mutation induces BD-like behavior in mouse models[4].

In addition, the significant window was notable in that it sits in a region of the genome that is dense for common variants based on the most recent PGC BD GWAS study of common variants[5]. Three distinct common variants associated within BD are within 2 Mb and five are within 6 Mb (**Table S9**). Relative to other 10kb windows in the genome this window was in the top 0.037% and 0.097% in terms of the maximum distance to the third and fifth closest variants respectively. The nearest lead variant at genome-wide significant loci for BD was 622kb away. Interestingly the window overlaps a near-significant loci (p-value ~7.0×10^{-8}) in the most recent PGC SZ GWAS study[6], while the closest genome-wide significant loci for SZ (p-value =1.8×10^{-9}) is 0.8 Mb away (**Figure S14B**).

We also investigated the chromatin state context of the window. We used chromatin state annotations of the genome for 127 cell and tissue types based on a 25-state ChromHMM model previously learned from 12 marks based on imputed data[7,8]. This region was notable in that the prevalence of the TxReg (transcribed and regulatory) state, which is associated with concurrently transcribed, promoter and enhancer chromatin marks, broadly across the 10kb window and across cell and tissue types (**Figure S14A, S14B**). On average 44.5% of bases in the 10kb window were assigned to the TxReg state in a cell or tissue type. The next most prevalent state in the window was TxEnh5' which covered 29.6% of bases on average and shared high levels of enhancer marks and some transcription marks with the TxReg state. To quantify how extreme the prevalence of these states are in the window relative to the rest of the genome, for each of the 10kb windows throughout the genome we computed the percentage of 200 base pair bins across all cell and tissue types that are assigned to

each state. The significant window ranked in the top 0.02% of all 10kb windows in the genome based on state TxReg and the top 0.08% based on TxEnh5' (**Figure S14C**). These results motivated us to inspect the most extreme locations in the genome in terms of the density of TxReg. Notably, the top 1st and 3rd ranking windows that represent unique loci are only less than 0.6 Mb away and overlap with the transcriptional sites of the long noncoding RNAs (lncRNAs) NEAT1 (chr11: 65,422,774-65,445,540) and MALAT1 (chr11: 65,497,688-65,506,516) respectively, both of which are distinguished among lncRNAs in their broad binding across the genome[9]. Notably, as indicated by GRID-seq (capturing of global RNA interactions with DNA by deep sequencing) data on the MM.1S cell line[9], the significant window resides in a broader region enriched for both *NEAT1* and *MALAT1* binding sites (**Figure S14B**). There has been emerging evidence linking lncRNAs to various brain disorders[10,11]. Specifically, studies have shown that NEAT1 is down-regulated in SZ[12] while *MALAT1* is down-regulated in BD[13].

Supplemental Tables

Table S1. InPSYght study samples by sub-cohort and case/control status

Study of origin	BD (N)	SZ (N)	Control (N)
GPC	1475	2855	2338
COGS	0	432	313
NIMH Repository: (STEP-BD, BIGS, Litmus)	123	8	0

Table S2. Source of TOPMed controls from 11 TOPMed studies

TOPMed study	Samples used as controls (N)	Sequencing Center
Cleveland Family Study (sleep apnea)	2	University of Washington (UW)
Barbados Asthma Genetics Study (BAGS)	997	Illumina
Genetic Epidemiology of COPD	3171	Broad
Multi-Ethnic Study of Atherosclerosis (MESA)	1207	Broad
African American Coronary Artery Calcification (AACAC); substudy of MESA	127	Broad
Atherosclerosis Risk in Communities (ARIC+VTE)	295	Baylor
Women's Health Initiative (WHI)	1455	Broad
Hypertension Genetic Epidemiology Network (HyperGen/GENOA)	2988	University of Washington (UW)
Sarcoidosis in African Americans	632	Baylor
BioMe Biobank	1360	Washington University (WashU)
CARDIA	1578	Baylor

Table S3. InPSYght sample demographic and sequencing wave characteristics

	SZ	BD	SZ + BD	Control	TOTAL
**N**	3295	1598	4893	2651	7544
**Females N (%)**	1036 (31%)	771 (48%)	1807 (34%)	1352 (51%)	3159 (42%)
**Age Years (SD) (N non-missing)**	43.7 (12.2) (3236)	43.1 (11.7) (1521)	43.5 (12.0) (4757)	40.6 (13.6) (2631)	42.5 (12.7) (7388)
**Sequencing Wave 0**	112	5	117	114	231
**1**	165	165	330	161	491
**2**	161	165	326	165	491
**3**	599	118	717	344	1061
**4**	1019	223	1242	516	1758
**5**	795	512	1307	744	2051
**6**	444	410	854	607	1461

Table S4. InPSYght sample quality control exclusions

Quality control	Individuals removed (N)
Duplicates	366
Sex mismatches	20
Non-XX/XY sex karyotypes	17
DNA contamination >5%	4
<98% of sites not at sequencing depth of 10	14
Unqualified controls	10

Table S5. Descriptions of the sequencing quality metrics and their loadings in the first four sequencing metadata PCs

Metric	PC1 Loading	PC2 Loading	PC3 Loading	PC4 Loading	Description
VB_DEPTH	0.70	0.72	0.02	0.03	Average depth estimated by verifyBamID2 (in array genotyped sites)
FREEMIX	0.00	0.00	0.00	-0.01	Contamination estimates by verifyBamID2
CONTAM_PC1	0.00	0.00	0.00	0.01	Estimated ancestry PC1,2,3,4 of contaminating samples estimated by verifyBamID2
CONTAM_PC2	0.00	0.00	0.01	-0.04	
CONTAM_PC3	0.00	0.00	0.00	0.00	
CONTAM_PC4	0.00	0.00	0.00	0.00	
FRAC_DP1	0.00	0.00	0.00	0.00	Fraction of bases covered by depths 1,5,10,15,20,30
FRAC_DP5	0.00	0.00	0.00	0.00	
FRAC_DP10	0.00	0.00	0.00	-0.01	
FRAC_DP15	0.00	0.00	0.00	-0.11	
FRAC_DP20	0.01	0.00	0.01	-0.52	
FRAC_DP30	0.03	0.01	0.03	-0.84	
RDP_X	0.00	-0.02	0.77	0.02	Relative depth of X (compared to autosomes)
RDP_Y	0.00	0.02	-0.64	-0.02	Relative depth of Y (compared to autosomes)
AUTO_DP	0.72	-0.70	-0.02	0.01	Autosome depth

Table S6. Summary of single-variant GWAS results. Significant threshold set to p<5E-9. Variant called on GRCh38.

Sample group 1	Sample group 2	Genomic inflation factor (λ)	Number of significant loci (variant(s)).	Notes
InPSYght controls	TOPMed controls	1.02	1 (chr13:79615934:CT:C)	Variant failed SVM filtering pipeline in TOPMed previous freeze; just passed filters in freeze9 (freeze used in current paper)
InPSYght BD + SZ	InPSYght + TOPMed controls	1.01	0 (0)	
	InPSYght controls	0.996	0 (0)	
InPSYght BD	InPSYght + TOPMed controls	1.02	1 (chr18:49738979:G:T; chr18:49741194:A:G; chr18:49744269:GGT:G)	No longer significant with inclusion of meta-PCs
	InPSYght controls	0.996	0 (0)	
InPSYght SZ	InPSYght + TOPMed controls	1.00	0 (0)	
	InPSYght controls	0.997	0 (0)	

Table S7. Non-reference allele frequencies of the 3 significant chromosome 18 variants (InPSYght bipolar versus InPSYght + TOMed controls single-variant association analysis) in cases, controls and the total TOPMed study *Obtained from files from the download page of https://imputationserver.sph.umich.edu/rsq-browser/

chr:bp:ref:alt (GRCh38)	rsID	Alternate allele frequency						
		InPSYght			TOPMed	InPSYght +TOPMed	Total TOPMed	
		BD	SZ	Controls	Controls	Controls	AFR*	EUR*
Chr18: 49738979:G:T	rs796645723	0.0069	0.0014	0.00057	0.0012	0.0011	0.0033	n/a
Chr18: 49741194:A:G	rs566405039	0.0069	0.0014	0.00075	0.0012	0.0011	0.0033	n/a
Chr18: 49744269:GGT:G	rs796880023	0.0069	0.0014	0.00057	0.0012	0.0011	0.0031	n/a

Table S8: Top 10 genes from burden testing of PTV variants with MAF < 1% in SZ + BD cases vs controls. * denotes a pseudogene or RNA gene.

Gene ID	Gene Symbol	OR	P-Value
ENSG00000186106	*ANKRD46*	0.4334	3.261e-06
ENSG00000133243	*BTBD2*	1.8568	6.980e-06
ENSG00000032444	*PNPLA6*	0.3177	1.193e-05
ENSG00000105278	*ZFR2*	2.9034	2.176e-04
ENSG00000250100	*RP13-644M16.3**	69.1349	6.465e-04
ENSG00000004534	*RBM6*	0.5863	9.170e-04
ENSG00000197093	*GAL3ST4*	0.5332	1.023e-03
ENSG00000137501	*SYTL2*	1.7598	1.090e-03
ENSG00000167604	*NFKBID*	0.5842	1.405e-03
ENSG00000232347	*RP11-488L18.8**	0.3703	1.532e-03

Table S9. Comparison of singleton PTV burden test results from top ten implicated schizophrenia genes identified in SCHEMA.

Gene	InPSYght (P-value)	SCHEMA OR (95% CI)	SCHEMA (P-value)
SETD1A	0.86	20.1 (5.68-108)	2.00e-12
CUL1	*NA*	36.1 (5.01-1570)	2.01e-09
XPO7	0.86	52.2 (7.84-2190)	7.18e-09
TRIO	0.89	5.02 (2.47-10.4)	6.35e-08
CACNA1G	0.85	3.09 (1.21-7.63)	4.57e-07
SP4	*NA*	9.37 (3.38-29.7)	5.08e-07
GRIA3	*NA*	Inf (4.73-Inf)	5.98e-07
GRIN2A	*NA*	18.1 (3.74-172)	7.37e-07
HERC1	0.31	3.51 (2.04-6.03)	1.26e-06
RB1CC1	0.53	10 (2.89-43.9)	2.00e-06

Table S10. Distances from the significant chromosome 11 window (chr11:64,859,972-64,869,939) to the closest GWAS loci reported in a PGC BD GWAS study[5], and the percentage of all 10kb genomic windows with smaller distance to their closest GWAS loci.

Nth closest GWAS locus	Distance to GWAS locus (bp)	Percentage of 10kb genomic windows with smaller distance to its Nth closest GWAS loci
1	622,550	2.59%
2	1,216,310	0.47%
3	1,692,155	0.037%
4	3,013,821	0.046%
5	5,806,865	0.097%
6	14,516,526	0.40%

Supplemental Figures

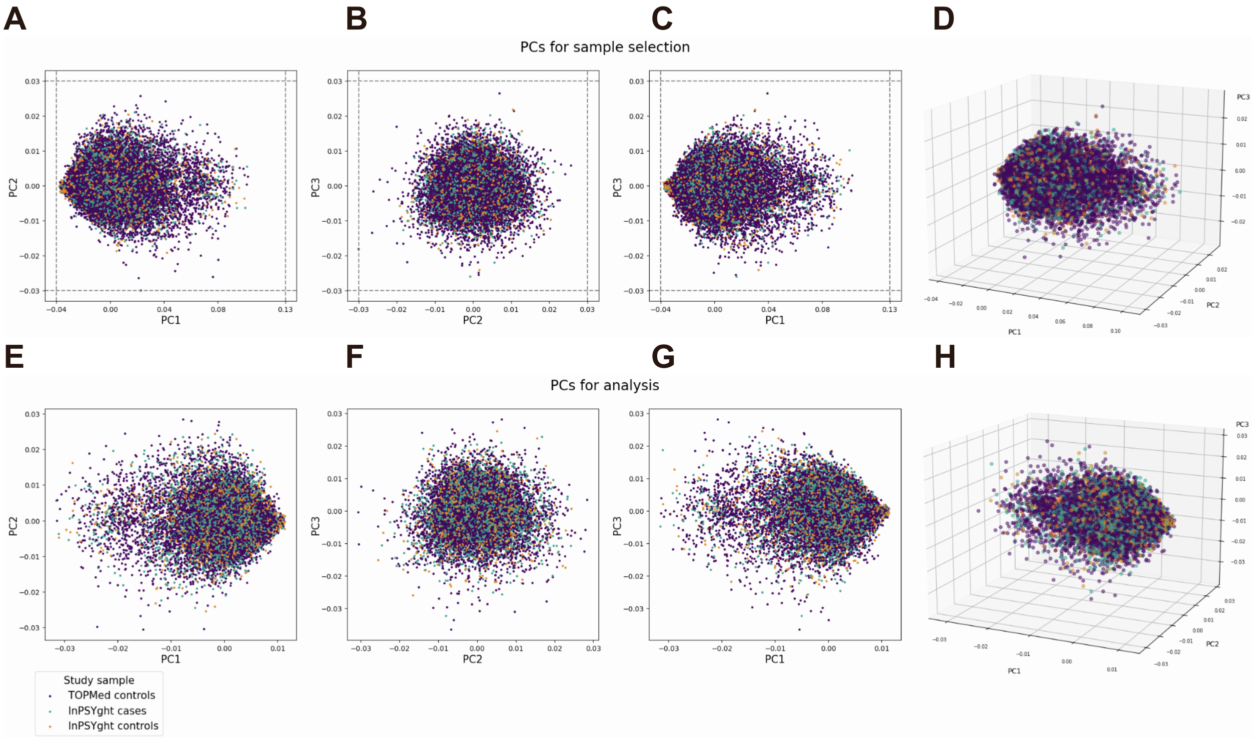

Figure S1. The first three genetic principal components (PCs) for the InPSYght and TOPMed samples. (A-D) PCs created using WGS'd samples, to further define WGS samples for inclusion. Only included samples are shown in the plots. Dashed lines indicate PC thresholds used for selection of included samples. (A) PC1 vs. PC2, (B) PC2 vs. PC3, (C) PC1 vs. PC3, and (D) PCs 1-3 in the 3D space. (E-H) PCs for association analysis created using WGS data from samples included in analysis. (E) PC1 vs. PC2, (F) PC2 vs. PC3, (G) PC1 vs. PC3 and (H) PCs 1-3 in the 3D space.

A

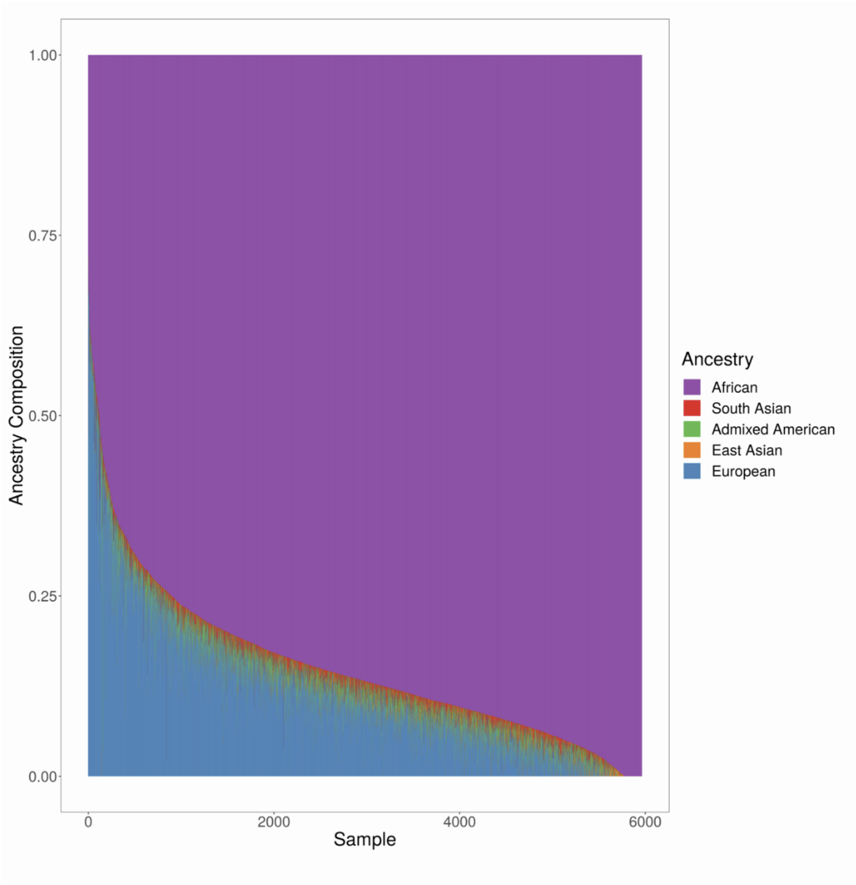

B

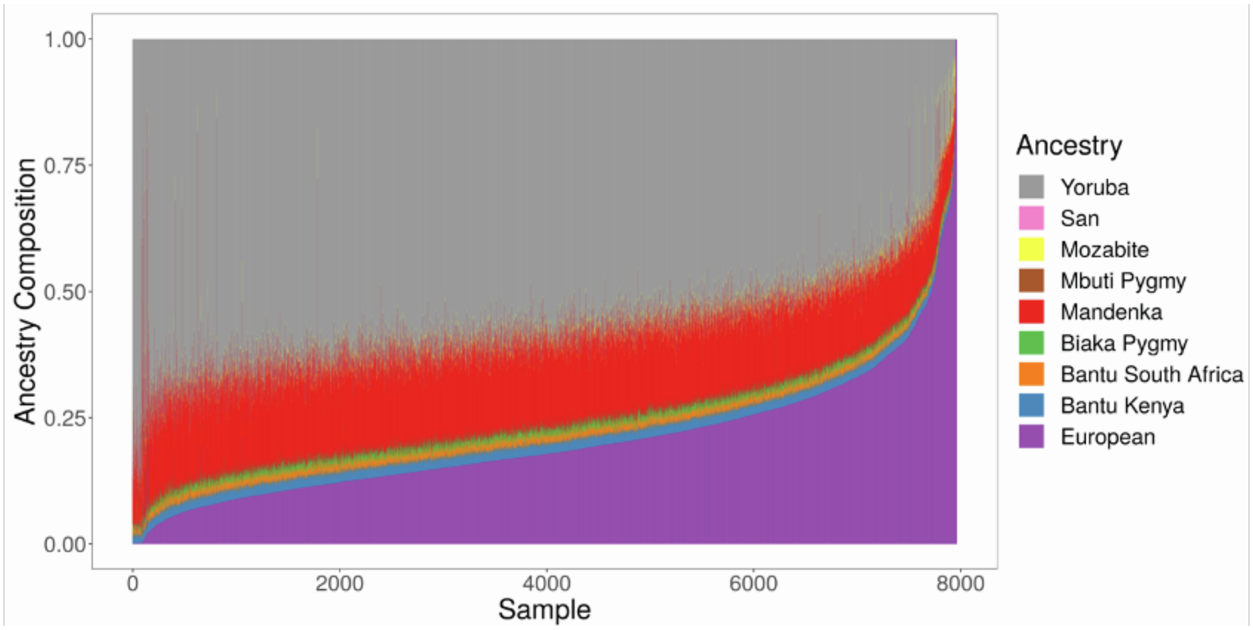

Figure S2. Estimated African and European ancestry composition of African American individuals within the InPSYght study. Global ancestry composition of African American individuals within the InPSYght study using 1000 Genomes super-populations (A) and

within African sub-populations (B) Reference populations include European as a super population and African sub populations from the HGDP reference panel. Note this ancestry analysis differs from the 1000 Genomes Project phase 3 ancestry super-populations ancestry estimation analysis from which proportion European ancestry was estimated for InPSYght study inclusion. The 1000 Genomes Project based analysis including Admixed Americans (AMR), South Asian (SAS), and East Asian (EAS) individuals.

A

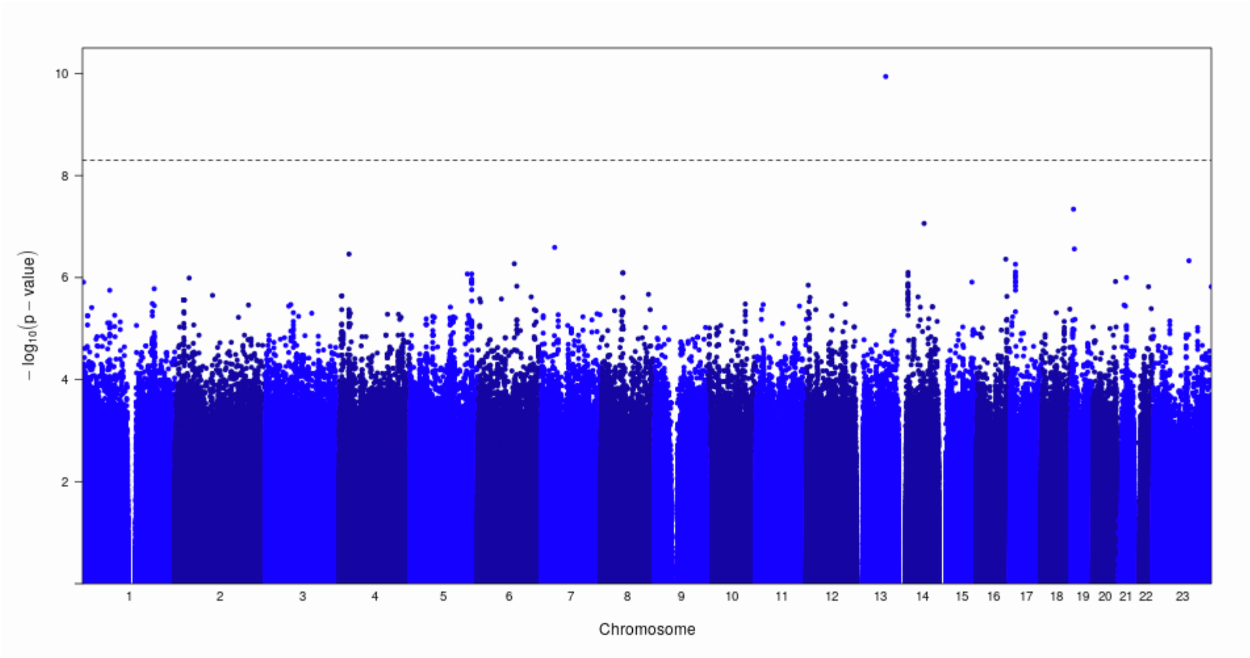

B

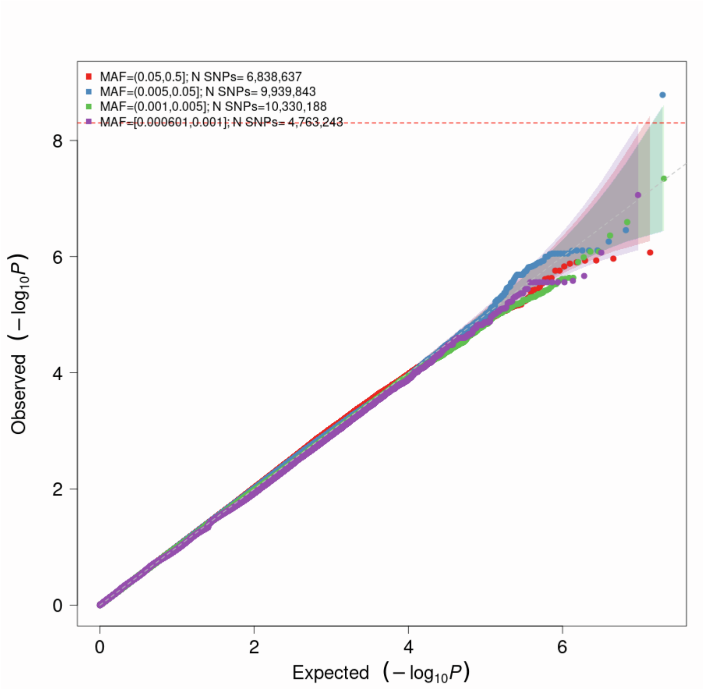

Figure S3. InPSYght controls versus TOPMed controls with sex and 10 PCs as covariates stratified by MAF of variants: Manhattan (A) and Quantile-Quantile Plots (B). Lambda GC: 1.02. Horizontal line shows genome-wide significance threshold of 5 x 10^{-9}.

A

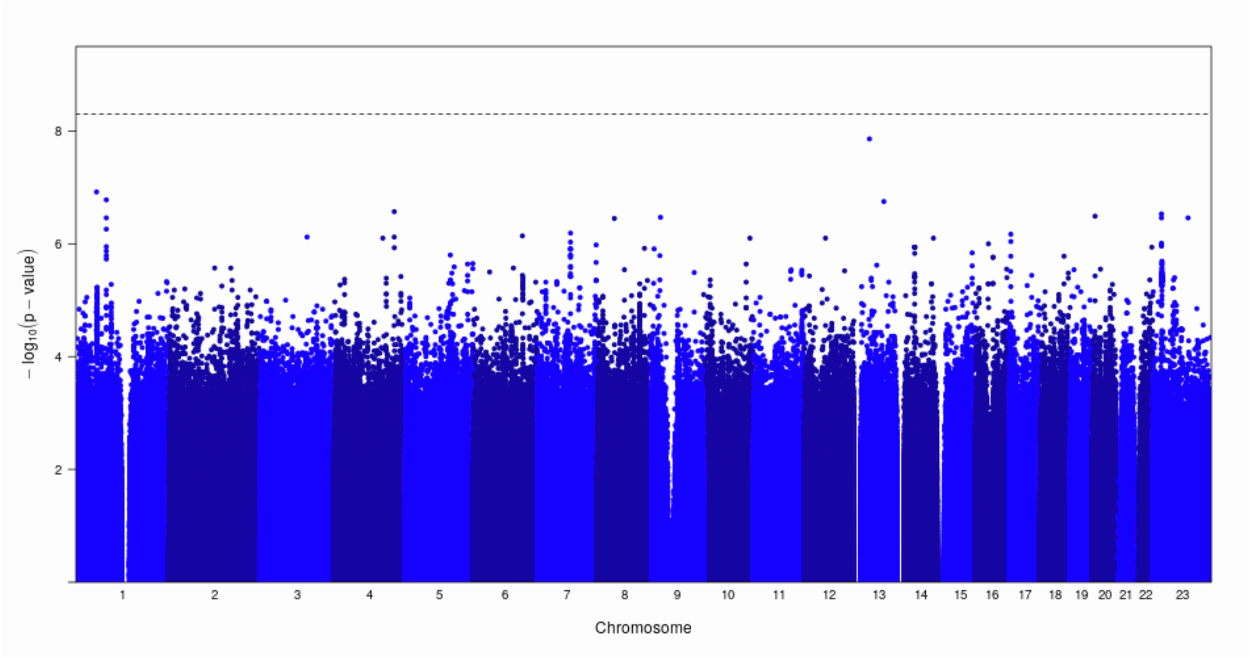

B

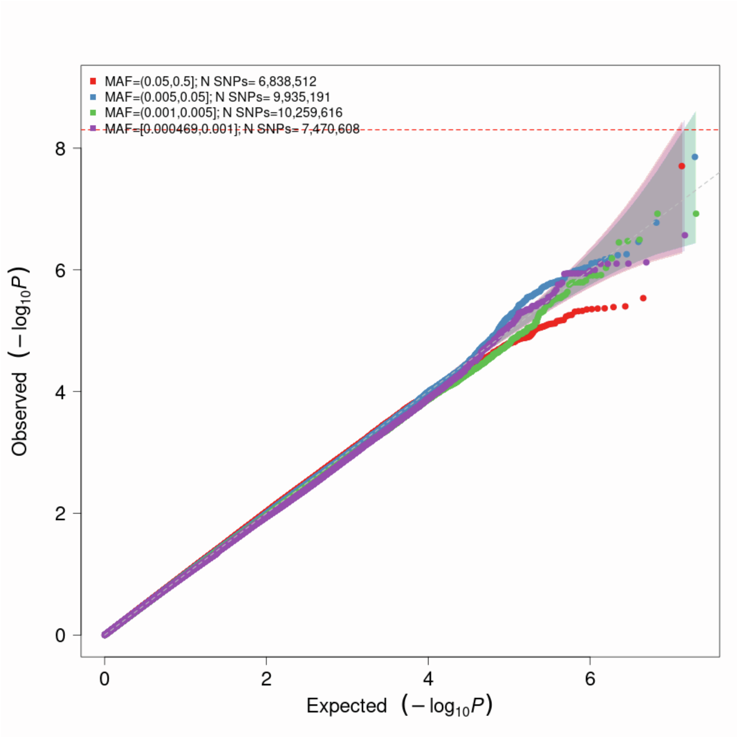

Figure S4. InPSYght cases with InPSYght + TOPMed controls with sex and 10 PCs as covariates stratified by MAF of variants: Manhattan (A) and Quantile-Quantile Plots (B). Lambda GC: 1.01. Horizontal line shows genome-wide significance threshold of 5 x 10^{-9}.

A

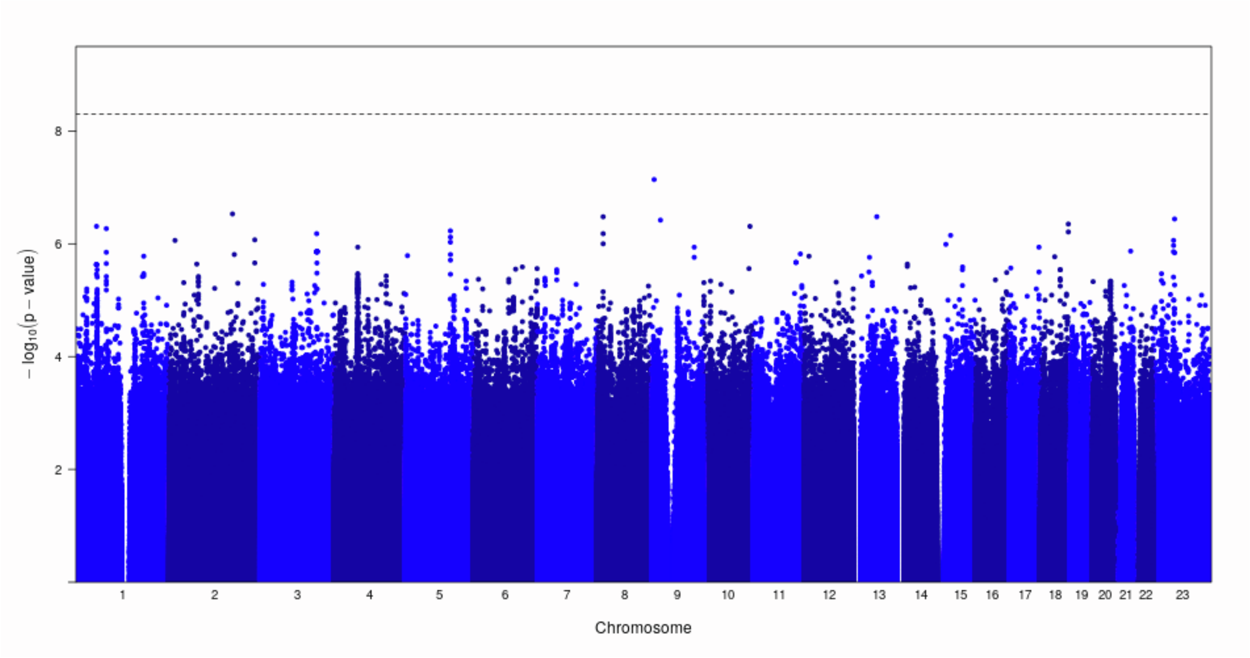

B

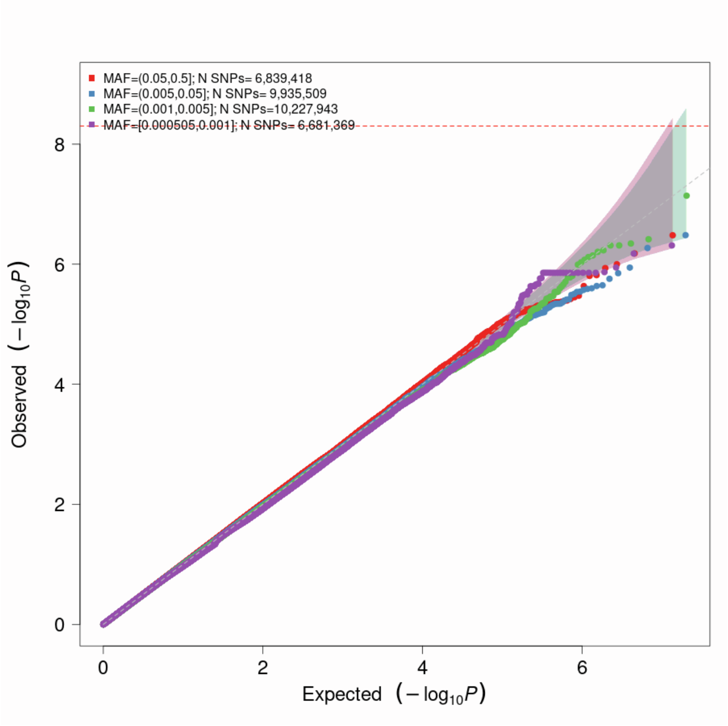

Figure S5. InPSYght schizophrenia cases versus InPSYght + TOPMed controls with sex and 10 PCs as covariates stratified by MAF of variants: Manhattan (A) and Quantile-Quantile Plots (B). Lambda GC: 1.00. Horizontal line shows genome-wide significance threshold of 5 x 10^{-9}.

A

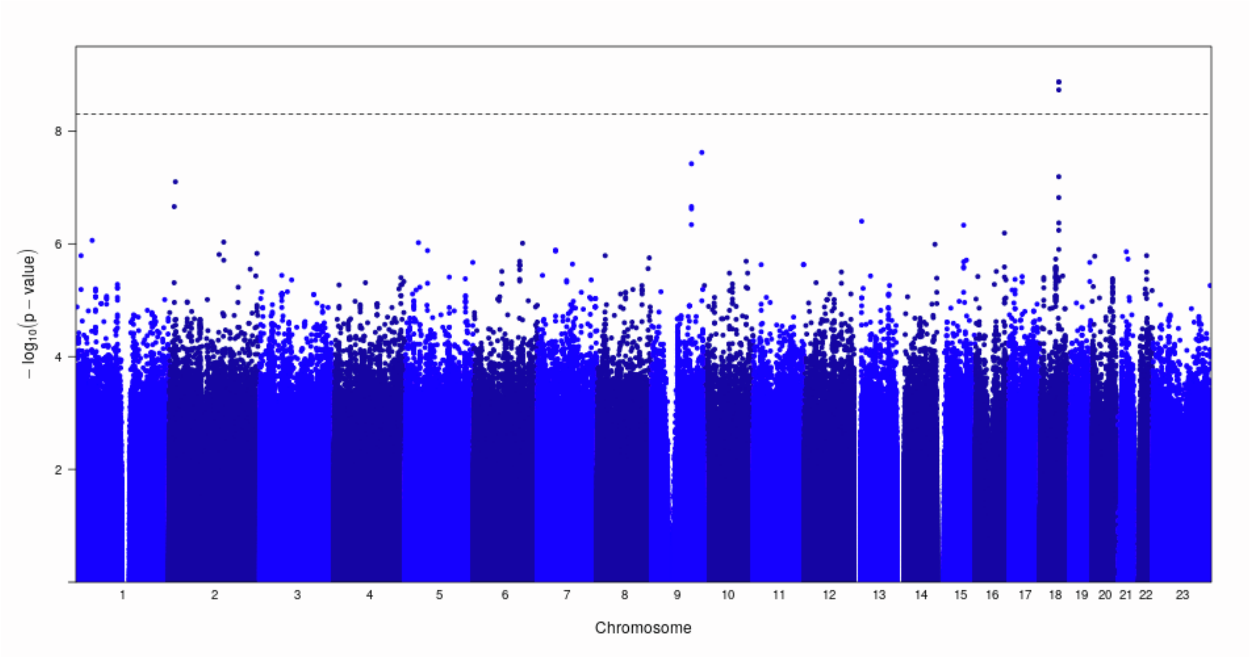

B

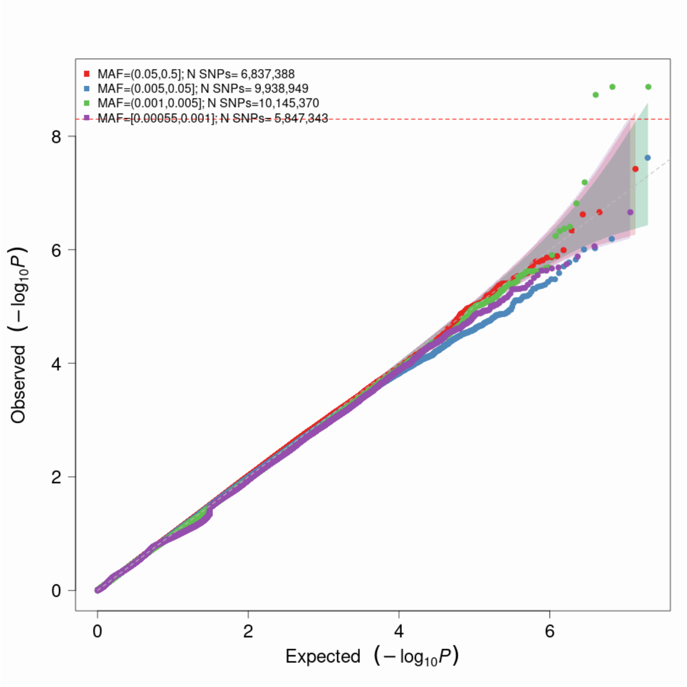

Figure S6. InPSYght bipolar cases versus InPSYght + TOPMed controls with sex and 10 PCs as covariates stratified by MAF of variants: Manhattan (A) and Quantile-Quantile Plots (B). Lambda GC: 1.02. Note: the Manhattan (Figure S6a is also presented as a main figure.) Horizontal line shows genome-wide significance threshold of 5 x 10^{-9}.

A

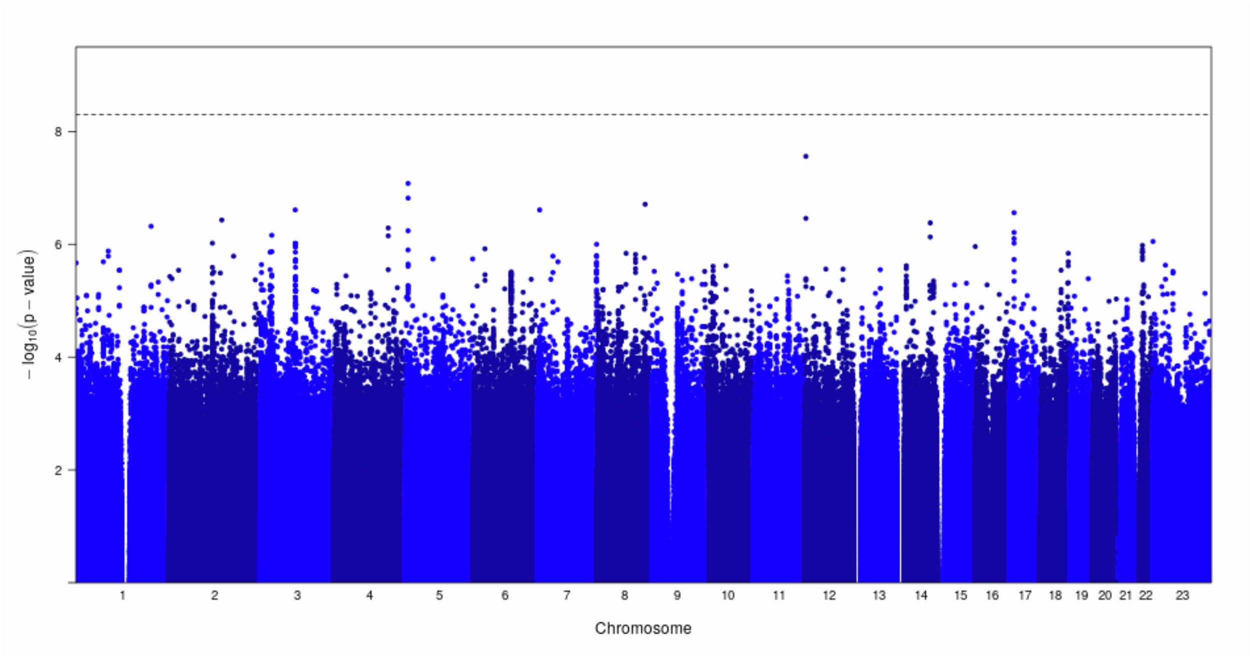

B

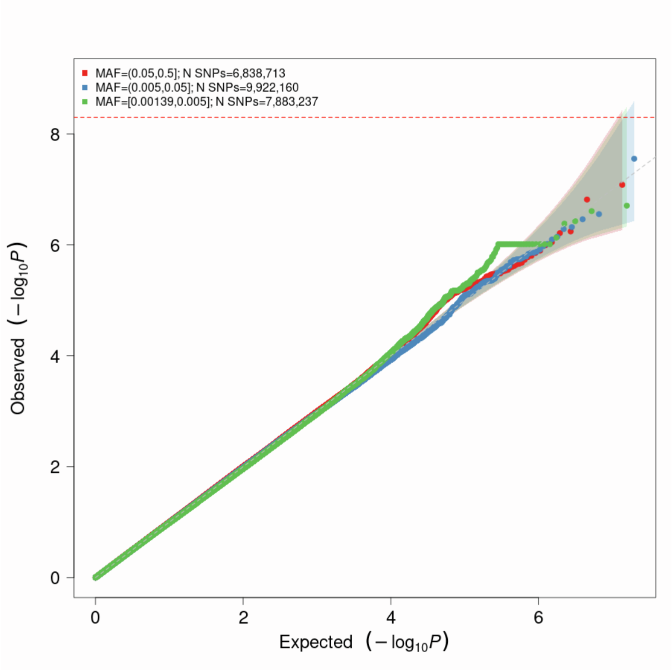

Figure S7. InPSYght-only cases versus controls with sex, batch and 10 PCs as covariates stratified by MAF of variants: Manhattan (A) and Quantile-Quantile Plots (B). Lamb GC: 1.00. Horizontal line shows genome-wide significance threshold of 5 x 10^{-9}.

A

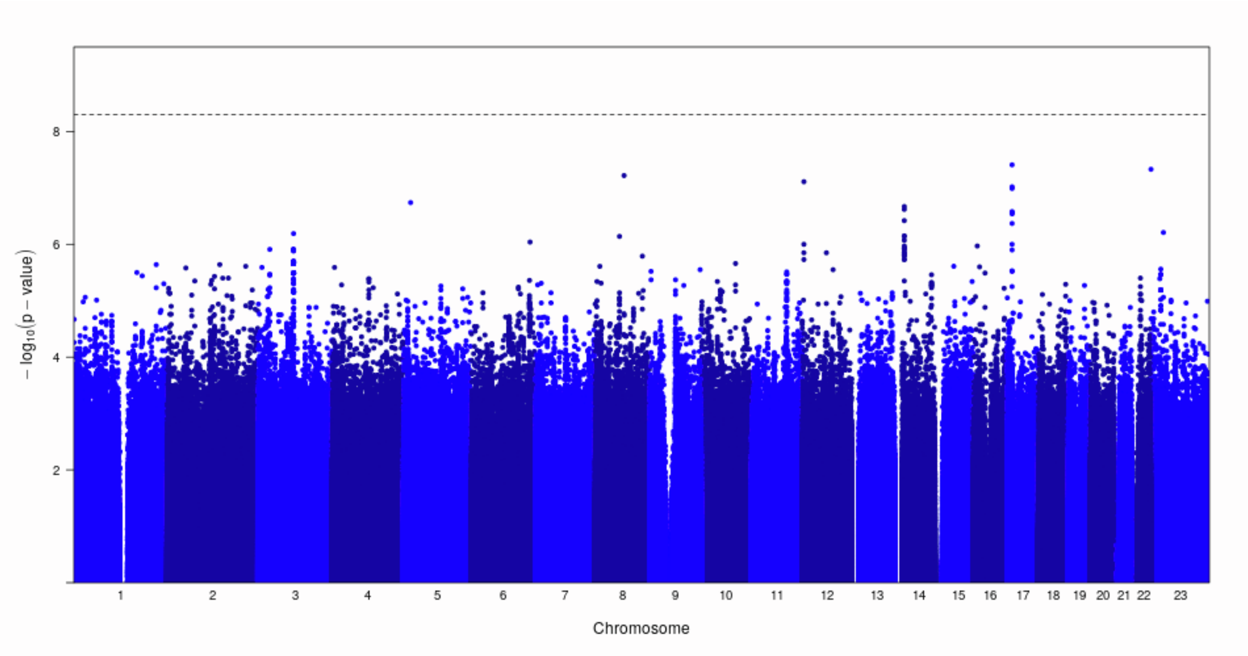

B

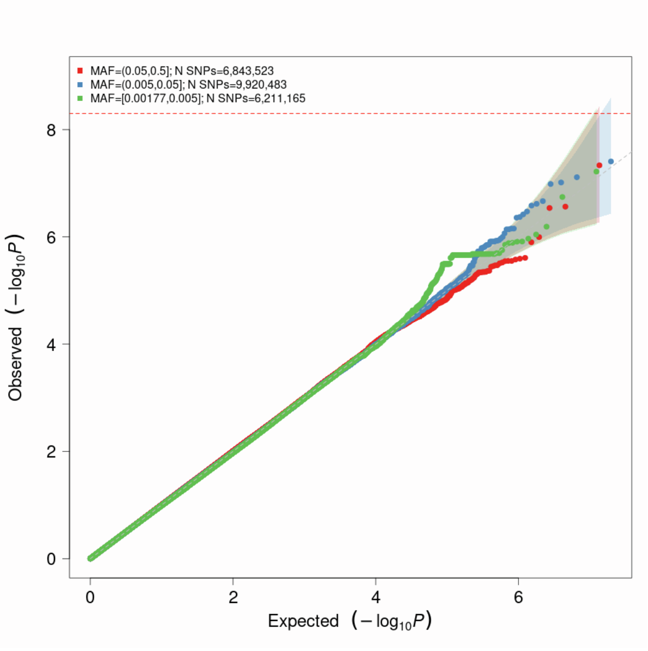

Figure S8. InPSYght-only schizophrenia cases versus controls with sex, batch and 10 PCs as covariates stratified by MAF of variants: Manhattan (A) and Quantile-Quantile Plots (B). Lambda GC: 1.00. Horizontal line shows genome-wide significance threshold of 5 x 10^{-9}.

A

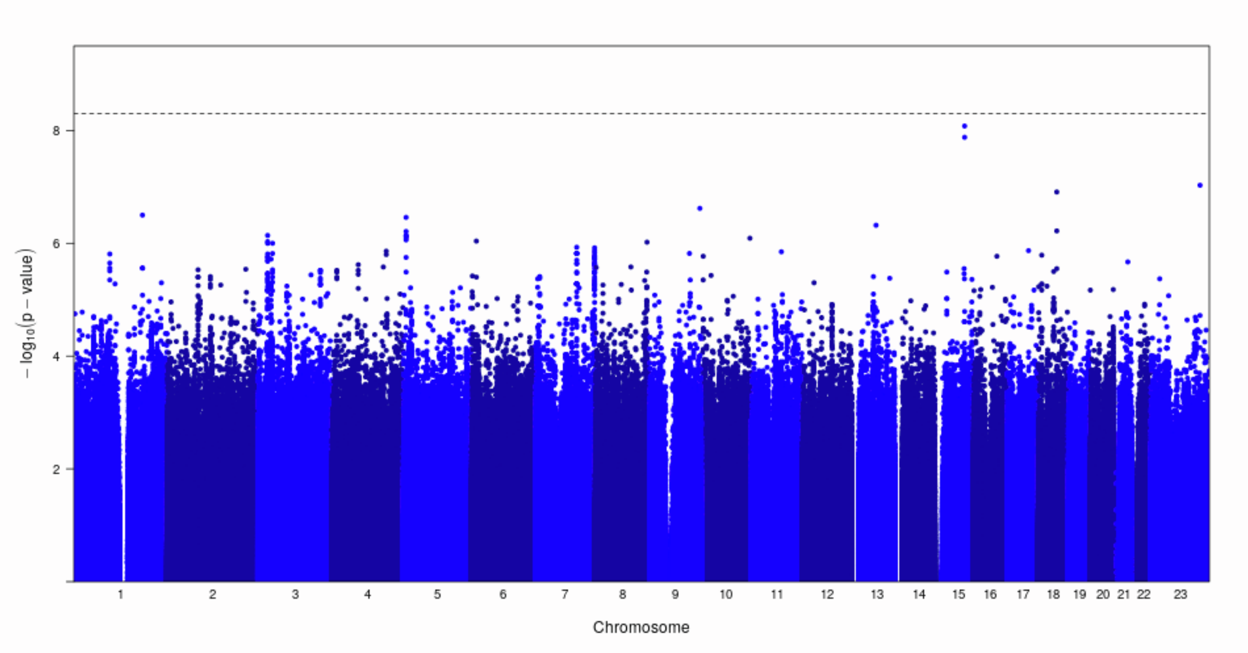

B

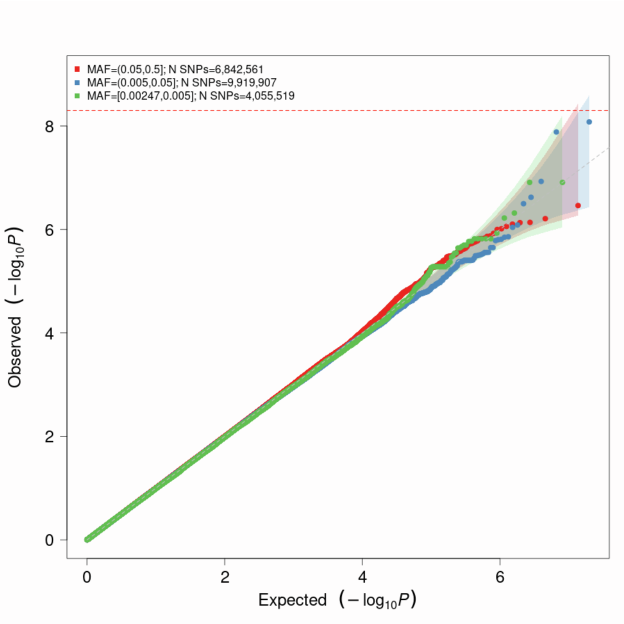

Figure S9. InPSYght-only bipolar cases versus controls with sex, batch and 10 PCs as covariates stratified by MAF of variants: Manhattan (A) and Quantile-Quantile Plots (B). Lambda GC: 1.00. Horizontal line shows genome-wide significance threshold of 5×10^{-9}.

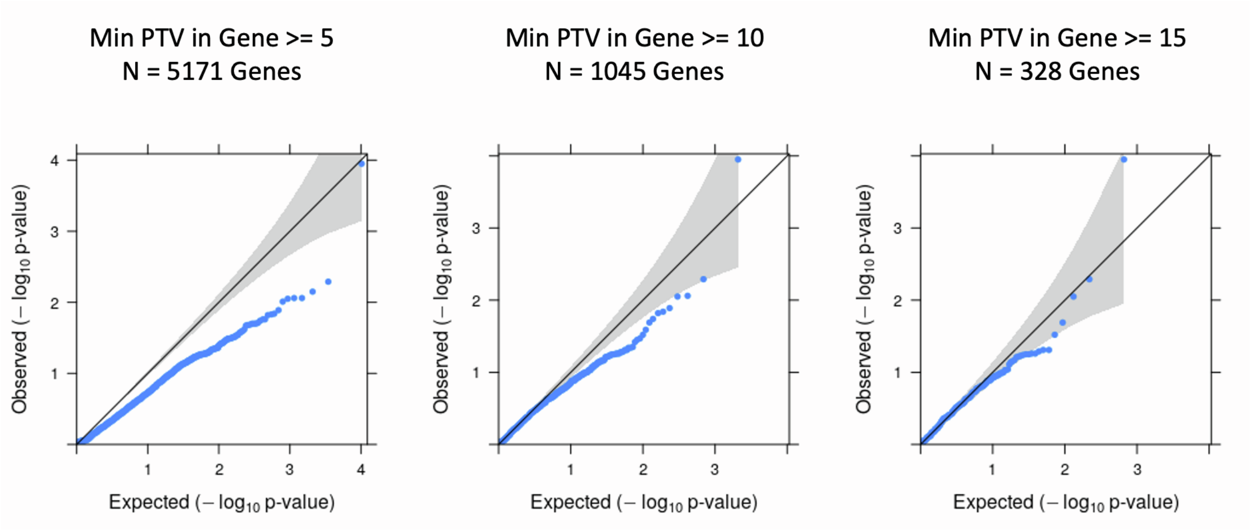

Figure S10. QQ plot of genome wide gene-level singleton PTV burden tests in unrelated individuals by minimum number of PTV criteria for gene inclusion. Covariates adjusted for include genetic PC1-10, sex, and total number of singletons.

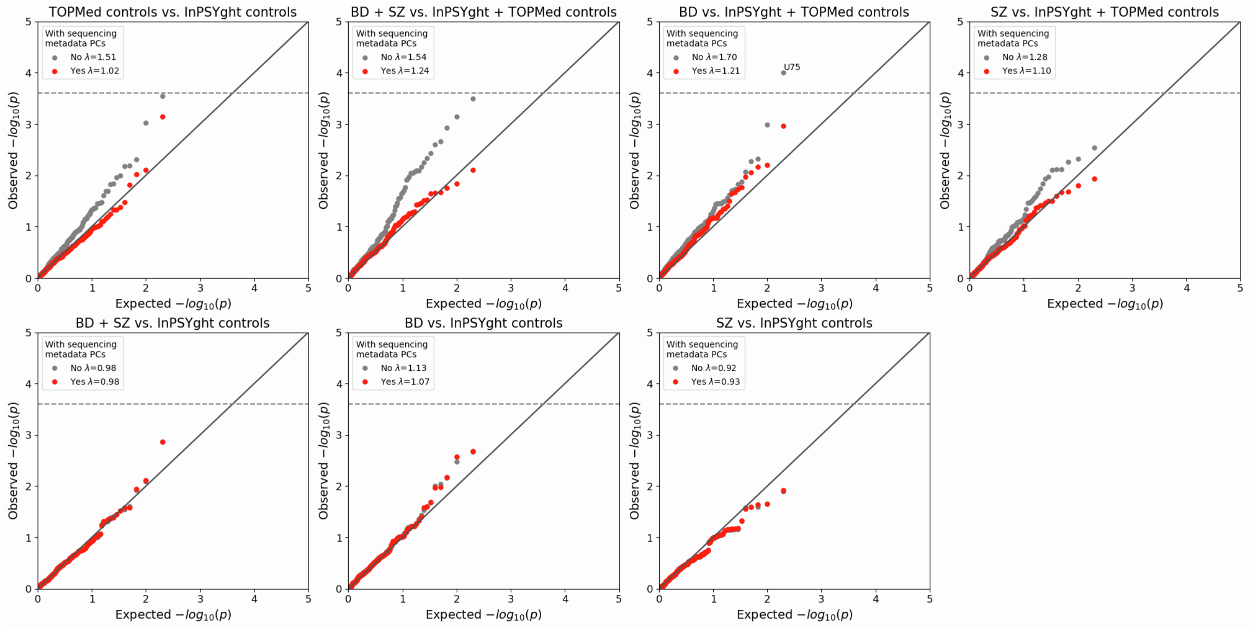

Figure S11. QQ-plots of chromatin and conservation state burden test results for different combinations of samples, showing their genomic inflation factors (λ) before and after including sequencing metadata PCs in the covariates. Horizontal lines show Bonferroni-based p-value thresholds (p=0.05/200). Diagonal lines show the unit slope.

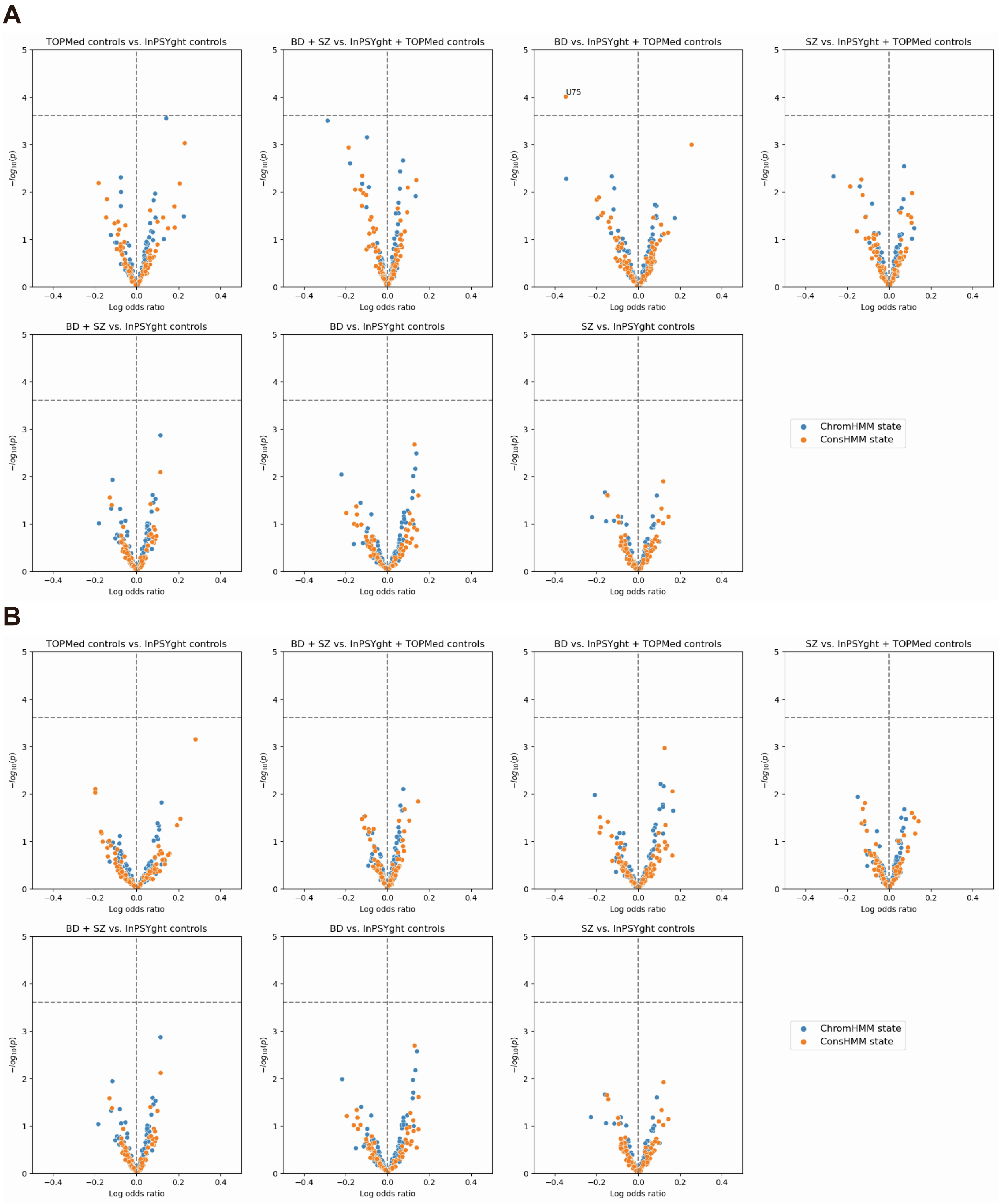

Figure S12. Volcano plots of chromatin and conservation state burden test results for different combinations of samples, before (A) and after (B) using sequencing metadata PCs as covariates, with log odds ratio on the x-axis and -log10 p-values on the y-axis. Vertical lines show x=0. Horizontal lines show Bonferroni-based p-value thresholds (p=0.05/200).

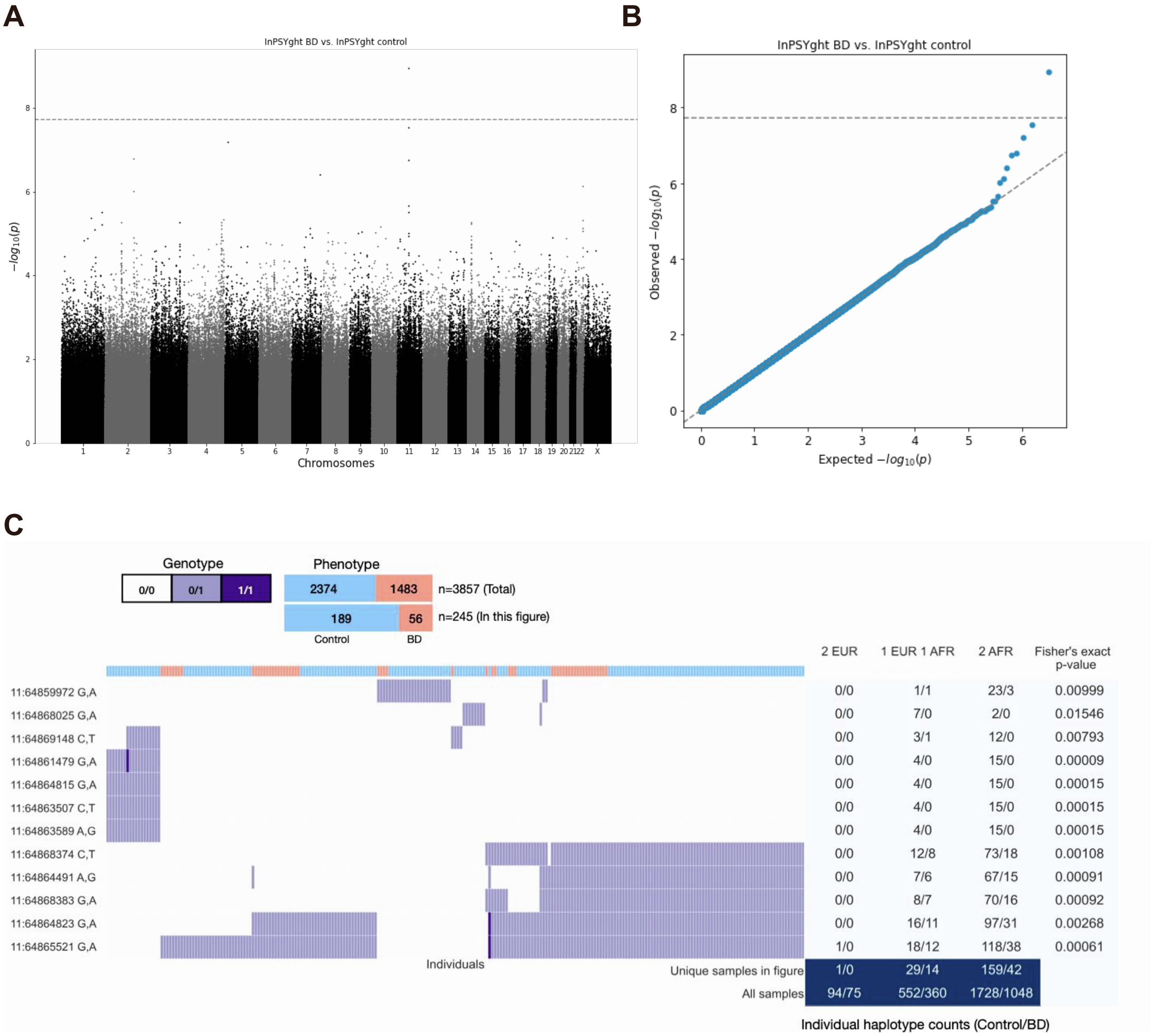

Figure S13. Summary of WGScan burden test results for InPSYght BD cases versus controls, with genomic repeat regions removed. BD: bipolar disorder. (**A**) Manhattan plot of all windows being tested; dashed line shows the Bonferroni-corrected p-value threshold based on WGScan's estimated number of effective tests (0.05 / 2,463,399 = 2.0×10^{-8}). (**B**) Quantile-quantile plot for burden p-values of all windows being tested. (**C**) Sample genotypes for the significant window chr11:64,859,972-64,859,939. The sites with nominally significant Fisher's exact p-values and the samples with at least 1 alternative allele observation among these sites are shown in the heatmap. The table on the right shows the ancestry haplotype distributions for samples having each variant and the Fisher's exact p-values for each variant.

A

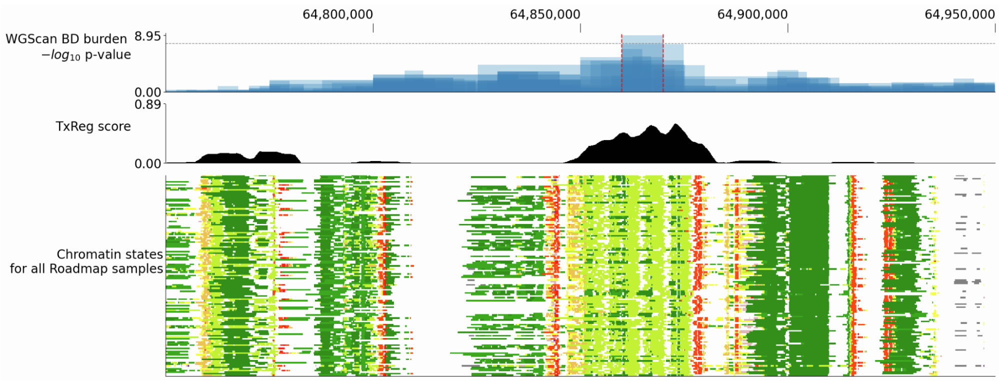

B

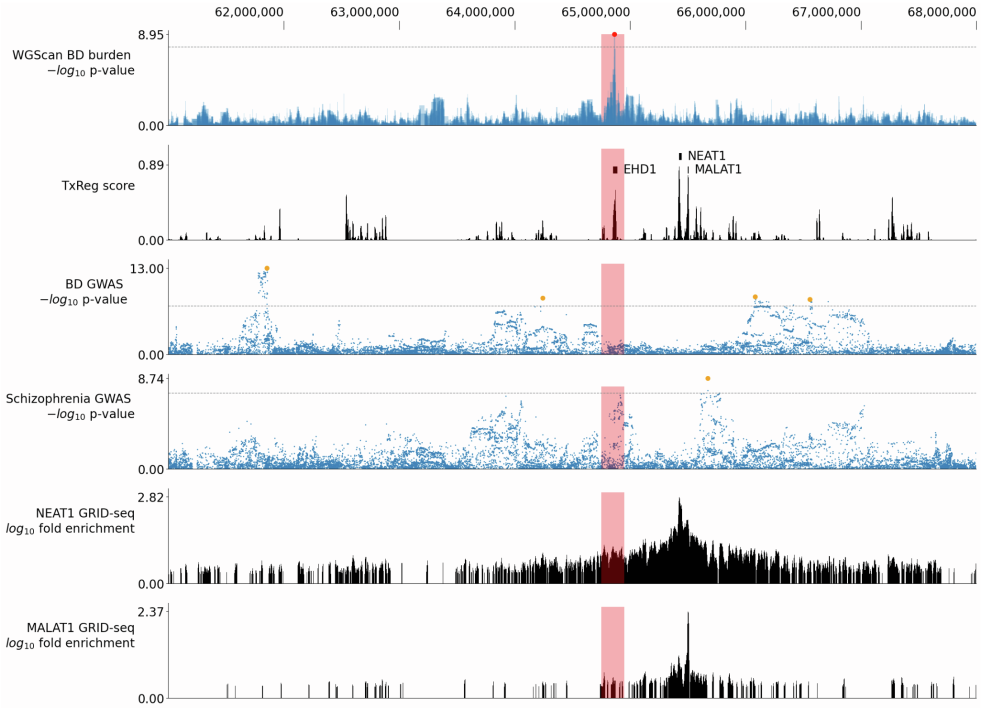

C

State	% of assignments in lead 10kb window	% 10kb windows >= frequency than lead
TxReg	**44.54**	**0.02**
TxEnh5'	29.61	0.08
TxEnhW	5.45	3.38
Tx5'	16.03	4.32
Tx	2.24	4.50
PromD2	0.09	6.89
Tx3'	0.13	17.40
EnhAF	0.02	23.17
TxWk	1.15	27.46
EnhAc	0.09	34.86
EnhA1	0.02	35.99
EnhA2	0.08	37.48
EnhWk2	0.19	50.73

Figure S14. Genomic region surrounding the significant window from WGScan BD burden analysis. (**A**) A smaller region surrounding the significant window. Top: windows tested by WGScan shown as bars with height corresponding to their -$\log_{10}$ p-values.

The significant window (chr11: 64,859,972-64,869,939) is indicated between the red dashed lines. Grey dashed line indicates the Bonferroni-corrected p-value threshold based on WGScan's estimated number of effective tests (0.05 / 2,463,399 = 2.0×10^{-8}). Middle: average TxReg score (computed as the percentage among all 127 Roadmap cell & tissue types that were assigned to the TxReg state) across 200bp bins of a 10kb window around the position. Bottom: chromatin state assignments using the 12-mark 25-state imputation-based ChromHMM annotations[8]. (**B**) A larger region surrounding the significant window (indicated as red boxes). From top to bottom: WGScan windows (same as in (**A**)), TxReg score (same as in (**A**)), GWAS summary statistics from the PGC BD study[5] and the PGC SZ study[6] with genome-wide significant loci shown as orange markers and genome-wide significance threshold shown as grey dashed lines, GRID-seq signal tracks for NEAT1 and MALAT1 on the MM.1S cell line[9]. (**C**) Percentages of 200bp bins assigned to each of the chromatin states (12-mark, 25-state ChromHMM model)[7,8] within the 10kb significant window, aggregated over 127 cell and tissue types, and the percentages of all 10kb windows throughout the genome with greater assignments.

TOPMed Study Short Acknowledgements with dbGaP accession numbers

Genome Sequencing for "NHLBI TOPMed: Cleveland Family Study - WGS Collaboration" (phs000954) was performed at NWGC (3R01HL098433-05S1).

Genome Sequencing for "NHLBI TOPMed: New Approaches for Empowering Studies of Asthma in Populations of African Descent - Barbados Asthma Genetics Study (BAGS)" (phs001143) was performed at Illumina (3R01HL104608-04S1).

Genome Sequencing for "NHLBI TOPMed: Genetic Epidemiology of COPD Study" (phs000951) was performed at NWGC and Broad Genomics (3R01HL089856-08S1, HHSN268201500014C, HHSN268201500014C).

Genome Sequencing for "NHLBI TOPMed: Multi-Ethnic Study of Atherosclerosis (MESA and MESA AA_CAC) "(phs001416.v1.p1) was performed at the Broad Institute of MIT and Harvard (3U54HG003067-13S1).

Genome Sequencing for "NHLBI TOPMed: Atherosclerosis Risk in Communities Study VTE cohort" (phs001211) was performed at Baylor (3U54HG003273-12S2 / HHSN268201500015C).

Genome Sequencing for "NHLBI TOPMed: Women's Health Initiative" (phs001237) was performed at Broad Genomics (HHSN268201500014C).

Genome Sequencing for "NHLBI TOPMed: Hypertension Genetic Epidemiology Network" (phs001293) was performed at NWGC (3R01HL055673-18S1).

Genome Sequencing for "NHLBI TOPMed: Sarcoidosis in African Americans" (phs001207) was performed at Baylor (3R01HL113326-04S1).

Genome Sequencing for "NHLBI TOPMed: Mount Sinai BioMe Biobank" (phs001644) was performed at Baylor (HHSN268201600033I).

Genome Sequencing for "NHLBI TOPMed: Coronary Artery Risk Development in Young Adults" (phs001612) was performed at Baylor (HHSN268201600033I).

TOPMed Study-specific Acknowledgements

Cleveland Family Study – WGS Collaboration (CFS)

The Cleveland Family Study has been supported in part by National Institutes of Health grants [R01-HL046380, KL2-RR024990, R35-HL135818, and R01-HL113338].

New Approaches for Empowering Studies of Asthma in Populations of African Descent - Barbados Asthma Genetics Study (BAGS)

We gratefully acknowledge the contributions of Pissamai and Trevor Maul, Paul Levett, Anselm Hennis, P. Michele Lashley, Raana Naidu, Malcolm Howitt and Timothy Roach, and the numerous health care providers, and community clinics and co-investigators who assisted in the phenotyping and collection of DNA samples, and the families and patients for generously donating DNA samples to the Barbados Asthma Genetics Study (BAGS). Funding for BAGS was provided by National Institutes of Health (NIH) R01HL104608, R01HL087699, and HL104608 S1.

Genetic Epidemiology of COPD Study (COPDGene)

The COPDGene study (NCT00608764) is supported by grants from the National Heart, Lung and Blood Institute (NHLBI; U01HL089897 and U01HL089856), by National Institutes of Health contract 75N92023D00011, and by the COPD Foundation through contributions made to an Industry Advisory Committee that has included AstraZeneca, Bayer Pharmaceuticals, Boehringer-Ingelheim, Genentech, GlaxoSmithKline, Novartis, Pfizer and Sunovion. A full listing of COPDGene investigators can be found at: http://www.copdgene.org/directory

Multi-Ethnic Study of Atherosclerosis (MESA & MESA AA_CAC)

Whole genome sequencing (WGS) for the Trans-Omics in Precision Medicine (TOPMed) program was supported by the National Heart, Lung and Blood Institute (NHLBI). WGS for “NHLBI TOPMed: Multi-Ethnic Study of Atherosclerosis (MESA)” (phs001416.v3.p1) was performed at the Broad Institute of MIT and Harvard (3U54HG003067-13S1). The MESA projects are conducted and supported by the National Heart, Lung, and Blood Institute (NHLBI) in collaboration with MESA investigators. Support for the Multi-Ethnic Study of Atherosclerosis (MESA) projects are conducted and supported by the National Heart, Lung, and Blood Institute (NHLBI) in collaboration with MESA investigators. Support for MESA is provided by contracts 75N92020D00001, HHSN268201500003I, N01-HC-95159, 75N92020D00005, N01-HC-95160, 75N92020D00002, N01-HC-95161, 75N92020D00003, N01-HC-95162, 75N92020D00006, N01-HC-95163, 75N92020D00004, N01-HC-95164, 75N92020D00007, N01-HC-95165, N01-HC-95166, N01-HC-95167, N01-HC-95168, N01-HC-95169, UL1-TR-000040, UL1-TR-001079, UL1-TR-001420, UL1TR001881, DK063491, and R01HL105756. The authors thank the other investigators, the staff, and the participants of the MESA study for their valuable contributions. A full list of participating MESA investigators and institutes can be found at http://www.mesa-nhlbi.org.

Atherosclerosis Risk in Communities study (ARIC VTE)

The Atherosclerosis Risk in Communities study has been funded in whole or in part with Federal funds from the National Heart, Lung, and Blood Institute, National Institutes of Health, Department of Health and Human Services, under Contract nos. (75N92022D00001, 75N92022D00002, 75N92022D00003, 75N92022D00004, 75N92022D00005). The authors thank the staff and participants of the ARIC study for their important contributions.

Whole genome sequencing (WGS) for the Trans-Omics in Precision Medicine (TOPMed) program was supported by the National Heart, Lung and Blood Institute (NHLBI). WGS for “NHLBI TOPMed: Atherosclerosis Risk in Communities (ARIC)” (phs001211) was performed at the Baylor College of Medicine Human Genome Sequencing Center (HHSN268201500015C and 3U54HG003273-12S2) and the Broad Institute for MIT and Harvard (3R01HL092577- 06S1).

Women’s Health Initiative (WHI)

The WHI program is funded by the National Heart, Lung, and Blood Institute, National Institutes of Health, U.S. Department of Health and Human Services through contracts 75N92021D00001, 75N92021D00002, 75N92021D00003, 75N92021D00004, 75N92021D00005.

Hypertension Genetic Epidemiology Network (HyperGen)

The HyperGEN Study is part of the National Heart, Lung, and Blood Institute (NHLBI)

Family Blood Pressure Program; collection of the data represented here was supported by grants U01 HL054472 (MN Lab), U01 HL054473 (DCC), U01 HL054495 (AL FC), and U01 HL054509 (NC FC). The HyperGEN: Genetics of Left Ventricular Hypertrophy Study was supported by NHLBI grant R01 HL055673 with whole-genome sequencing made possible by supplement -18S1.

Genetics of Sarcoidosis in African Americans (Sarcoidosis)

National Institutes of Health (R01HL113326, P30 GM110766-01)

Mount Sinai BioMe Biobank (BioMe)

The Mount Sinai BioMe Biobank has been supported by The Andrea and Charles Bronfman Philanthropies and in part by Federal funds from the NHLBI and NHGRI (U01HG00638001; U01HG007417; X01HL134588). We thank all participants in the Mount Sinai Biobank. We also thank all our recruiters who have assisted and continue to assist in data collection and management and are grateful for the computational resources and staff expertise provided by Scientific Computing at the Icahn School of Medicine at Mount Sinai.

Coronary Artery Risk Development in Young Adults (CARDIA)

The Coronary Artery Risk Development in Young Adults Study (CARDIA) is conducted and supported by the National Heart, Lung, and Blood Institute (NHLBI) in collaboration with the University of Alabama at Birmingham (75N92023D00002 & 75N92023D00005), Northwestern University (75N92023D00004), University of Minnesota

(75N92023D00006), and Kaiser Foundation Research Institute (75N92023D00003). CARDIA was also partially supported by the Intramural Research Program of the National Institute on Aging (NIA) and an intra-agency agreement between NIA and NHLBI (AG0005).

TOPMed Neurocognitive Working Group

Last Name	First Name	Affiliation
Almeida	Marcio	University of Texas Rio Grande Valley School of Medicine
Ament	Seth	University of Maryland
Ammous	Farah	University of Michigan
Arnett	Donna	University of South Carolina
Becker	Diane	Johns Hopkins University
Bis	Joshua	University of Washington
Blue	Elizabeth	University of Washington
Boerwinkle	Eric	University of Texas Health at Houston
Bressler	Jan	University of Texas Health at Houston
Cammann	Davis	University of Nevada Las Vegas
Chaar	Dima	University of Michigan
Chen	Jingchun	University of Nevada Las Vegas
Clarkson-Townsend	Danielle	Brigham & Women's Hospital
Coresh	Josef	Johns Hopkins University

DeStefano	Anita	Boston University
Deters	Kacie	University of California, Los Angeles
Ding	Jingzhong	Wake Forest Baptist Health
Fardo	David	University of Kentucky
Fitzpatrick	Annette	University of Washington
Fornage	Myriam	University of Texas Health at Houston
French	Jennifer	University of Maryland
Glahn	David	Boston Children's Hospital, Harvard Medical School
Gonzalez	Hector	University of California, San Diego
Granot-Hershkovitz	Einat	Brigham & Women's Hospital
Hanly	Patrick	University of Calgary
Hayden	Kathleen	Wake Forest Baptist Health
Heckbert	Susan	University of Washington
Horvath	Steve	University of California, Los Angeles
Hoth	Karin	University of Iowa
Hughes	Timothy	Wake Forest Baptist Health

Jaiswal	Sidd	Stanford University
Jian	Xueqiu	University of Texas Health at San Antonio
Katsumata	Yuriko	University of Kentucky
Kho	Minjung	University of Michigan
Kooperberg	Charles	Fred Hutchinson Cancer Research Center
Launer	Lenore	National Institute on Aging
Lin	Honghuang	Boston University
Litkowski	Elizabeth	University of Colorado Anschutz Medical Campus
Longstreth	Will	University of Washington
Mayeux	Richard	Columbia University
Mikulla	Julie	National Heart, Lung, and Blood Institute
Misra	Biswapriya	Wake Forest Baptist Health
Mosley	Thomas	University of Mississippi
Nyquist	Paul	Johns Hopkins University
O'Connell	Jeff	University of Maryland
Olivier	Michael	Wake Forest Baptist Health
Panyard	Daniel	Stanford University

Peloso	Gina	Boston University
Perry	James	University of Maryland
Psaty	Bruce	University of Washington
Purcell	Shaun	Mass General Brigham
Raffield	Laura	University of North Carolina
Reiner	Alex	Fred Hutchinson Cancer Research Center, University of Washington
Rotter	Jerome	Lundquist Institute
Sargurupremraj	Muralidharan	University of Texas Health at San Antonio
Sarnowski	Chloé	Boston University, University of Texas Health at Houston
Satizabal	Claudia	Boston University
Schellenberg	Gerard	University of Pennsylvania
Seo	Jungkyun	University of North Carolina
Seshadri	Sudha	University of Texas Health at San Antonio
Shade	Lincoln	University of Kentucky
Short	Meghan	Boston University
Simino	Jeannette	University of Mississippi

Smith	Jennifer	University of Michigan
Smoller	Sylvia	Albert Einstein College of Medicine
Snively	Beverly	Wake Forest Baptist Health
Soemedi	Rachel	Brigham & Women's Hospital
Sokolow	Sophie	University of California, Los Angeles
Thornton	Timothy	University of Washington
Vivek	Sithara	University of Minnesota
Wood	Alexis	Baylor College of Medicine
Yanek	Lisa	Johns Hopkins University
Yang	Qiong	Boston University
Yu	Miao	University of Michigan
Zare	Habil	University of Texas Health at San Antonio
Zhao	Wei	University of Michigan

Supplemental References

1. Wei, S., Xu, Y., Shi, H., Wong, S.-H., Han, W., Talbot, K., Hong, W., and Ong, W.-Y. (2010). EHD1 is a synaptic protein that modulates exocytosis through binding to snapin. Mol. Cell. Neurosci. *45*, 418–429. https://doi.org/10.1016/j.mcn.2010.07.014.
2. Yap, C.C.C., Lasiecka, Z.M., Caplan, S., and Winckler, B. (2010). Alterations of EHD1/EHD4 Protein Levels Interfere with L1/NgCAM Endocytosis in Neurons and Disrupt Axonal Targeting. J. Neurosci. *30*, 6646–6657. https://doi.org/10.1523/JNEUROSCI.5428-09.2010.
3. Kataoka, M., Matoba, N., Sawada, T., Kazuno, A.-A., Ishiwata, M., Fujii, K., Matsuo, K., Takata, A., and Kato, T. (2016). Exome sequencing for bipolar disorder points to roles of de novo loss-of-function and protein-altering mutations. Mol. Psychiatry *21*, 885–893. https://doi.org/10.1038/mp.2016.69.
4. Nakamura, T., Nakajima, K., Kobayashi, Y., Itohara, S., Kasahara, T., Tsuboi, T., and Kato, T. (2021). Functional and behavioral effects of de novo mutations in calcium-related genes in patients with bipolar disorder. Hum. Mol. Genet. *30*, 1851–1862. https://doi.org/10.1093/hmg/ddab152.
5. Mullins, N., Forstner, A.J., O'Connell, K.S., Coombes, B., Coleman, J.R.I., Qiao, Z., Als, T.D., Bigdeli, T.B., Børte, S., Bryois, J., et al. (2021). Genome-wide association study of more than 40,000 bipolar disorder cases provides new insights into the underlying biology. Nat. Genet. *53*, 817–829. https://doi.org/10.1038/s41588-021-00857-4.
6. Trubetskoy, V., Pardiñas, A.F., Qi, T., Panagiotaropoulou, G., Awasthi, S., Bigdeli, T.B., Bryois, J., Chen, C.-Y., Dennison, C.A., Hall, L.S., et al. (2022). Mapping genomic loci implicates genes and synaptic biology in schizophrenia. Nature *604*, 502–508. https://doi.org/10.1038/s41586-022-04434-5.
7. Ernst, J., and Kellis, M. (2012). ChromHMM: automating chromatin-state discovery and characterization. Nat. Methods *9*, 215–216. https://doi.org/10.1038/nmeth.1906.
8. Ernst, J., and Kellis, M. (2015). Large-scale imputation of epigenomic datasets for systematic annotation of diverse human tissues. Nat. Biotechnol. *33*, 364–376. https://doi.org/10.1038/nbt.3157.
9. Li, X., Zhou, B., Chen, L., Gou, L.-T., Li, H., and Fu, X.-D. (2017). GRID-seq reveals the global RNA–chromatin interactome. Nat. Biotechnol. *35*, 940–950. https://doi.org/10.1038/nbt.3968.
10. Yang, S., Lim, K.-H., Kim, S.-H., and Joo, J.-Y. (2021). Molecular landscape of long noncoding RNAs in brain disorders. Mol. Psychiatry *26*, 1060–1074. https://doi.org/10.1038/s41380-020-00947-5.
11. Srinivas, T., Mathias, C., Oliveira-Mateos, C., and Guil, S. (2023). Roles of lncRNAs in brain development and pathogenesis: Emerging therapeutic opportunities. Mol. Ther. *31*, 1550–1561. https://doi.org/10.1016/j.ymthe.2023.02.008.
12. Katsel, P., Roussos, P., Fam, P., Khan, S., Tan, W., Hirose, T., Nakagawa, S., Pletnikov, M.V., and Haroutunian, V. (2019). The expression of long noncoding RNA NEAT1 is reduced in schizophrenia and modulates oligodendrocytes transcription. Npj Schizophr. *5*, 3. https://doi.org/10.1038/s41537-019-0071-2.
13. Shirvani Farsani, Z., Zahirodin, A., Ghaderian, S.M.H., Shams, J., and Naghavi Gargari, B. (2020). The role of long non-coding RNA MALAT1 in patients with bipolar disorder. Metab. Brain Dis. *35*, 1077–1083. https://doi.org/10.1007/s11011-020-00580-9.
